# Construction and Characterization of Paired Patient-Derived Cell Models for Solitary Fibrous Tumors (SFTs)

**DOI:** 10.3390/cancers18152385

**Published:** 2026-07-24

**Authors:** Farhan Vahdat Azad, Paulina I. Trevino, Aishwarya Kappagantu, Maliha Mehjabin, Eleni Balla, Clark A. Meyer, Leonidas Bleris, Yi Li

**Affiliations:** 1 Department of Bioengineering, University of Texas at Dallas, Richardson, TX 75080, USA; farhan.vahdatazad@utdallas.edu (F.V.A.); paulina.trevino@utdallas.edu (P.I.T.);; 2 Department of Biological Sciences, University of Texas at Dallas, Richardson, TX 75080, USA; 3 Center for Systems Biology, University of Texas at Dallas, Richardson, TX 75080, USA

**Keywords:** solitary fibrous tumor (SFT), NAB2::STAT6, short hairpin RNA (shRNA)

## Abstract

**Introduction:** Solitary fibrous tumor (SFT) is a rare mesenchymal neoplasm that can arise across a wide range of anatomical locations, most commonly within the thoracic cavity, including the pleura. Surgical resection and radiotherapy are often first-line treatments for SFT. However, tumors that are not amenable to these conventional approaches require systemic therapy, for which effective options remain limited. In 2013, the NAB2::STAT6 fusion gene was discovered as the critical oncogenic driver of SFT. Nevertheless, the underlying molecular mechanisms by which the NAB2::STAT6 fusion event drives cancer progression remain largely unknown, primarily due to the lack of proper SFT and control cell models. **Aims:** To address this challenge, we used the SFT patient-derived cell line, INT-SFT, and performed short hairpin RNA (shRNA)-mediated downregulation to interrogate fusion-associated effects. **Results:** Phenotypic assays confirmed the oncogenic effects of the NAB2::STAT6 fusion transcript. Next, using transcriptomic and proteomic analyses, we identified genes and proteins regulated by the NAB2::STAT6 fusion and delineated its effects on key signaling pathways. **Conclusions:** Taken together, our SFT cell models, derived from the same genetic and clinical background but with different expression levels of the fusion transcript, represent a valuable resource for studying etiology and exploring novel therapeutics for SFTs.

## Simple Summary

Solitary fibrous tumor is a rare type of cancer that develops in connective tissues and can occur in many parts of the body. Although surgery and radiation therapy are often effective, some tumors cannot be treated with these approaches, and better treatment options are urgently needed. A distinct fusion gene (NAB2::STAT6) is known to drive the development of this cancer, but how it promotes tumor growth is still poorly understood. In this study, we developed cell models from a patient with solitary fibrous tumor and reduced the expression of the fusion gene to study its effects. By analyzing changes in gene and protein expression patterns, we identified biological pathways controlled by the fusion gene that may contribute to tumor growth. These new cell models provide an important resource for understanding how this rare cancer develops and for identifying potential targets for future therapies.

## 1. Introduction

Solitary fibrous tumor (SFT) is a rare mesenchymal neoplasm that can arise across a wide range of anatomical locations, including most commonly the pleura, but also extrapleural sites such as the abdomen, head and neck, and central nervous system [1,2]. The clinical manifestations of SFTs are highly variable; although many cases are considered benign, local or distant recurrences have been reported in approximately 10–30% of patients, and metastasis can occur in up to 35–45% of histologically aggressive tumors [3,4,5]. Currently, most SFTs are primarily managed with surgical resection. Tumors that are not amenable to curative surgery require systemic therapy, for which effective treatments remain limited [6].

Genetically, SFT is characterized by a somatic NAB2::STAT6 gene fusion on chromosome 12q13, which serves as a specific molecular hallmark for diagnosis [7,8,9]. Different fusion variants have been reported and are associated with distinct tumor locations and clinical behaviors [10]. The NAB2::STAT6 fusion protein is hypothesized to function as an oncogenic transcriptional factor. Current models suggest that the fusion protein localizes to the nucleus and drives EGR1 activation, leading to increased expression of downstream targets such as IGF2 and FGFR1 [11,12,13]. However, it should be noted that these mechanistic studies often relied on engineered cell models not derived from SFTs, including human embryonic kidney cells 293T [8], human osteosarcoma cells U2OS [14], human colorectal carcinoma cells HCT116 [15], and human mesenchymal stem cells [16]. Additionally, in reports where primary SFT cells or tissues were used, normal immortalized fibroblast cells were often included as negative controls [17,18], despite clinical evidence of high morphological variability in SFTs [19].

To address these challenges, in this study, we adopted the patient-derived cell line INT-SFT [18] and performed short hairpin RNA (shRNA)-mediated knockdown to create a pair of SFT cell models with identical clinical and molecular backgrounds, differing primarily in fusion transcript expression. Next, phenotypic and genomic assays were performed on these cell lines to elucidate fusion-associated signaling pathways.

## 2. Materials and Methods

### 2.1. Mammalian Cell Culture

The INT-SFT cell line was established by Dr. Roberta Maestro’s group at the Oncology Referral Center (Centro di Riferimento Oncologico) using SV40 Large T Antigen-mediated immortalization. The INT-SFT, INT-SFT-H, and INT-SFT-L cell lines were cultured in RPMI-1640 medium (Thermo Fisher Scientific, Waltham, MA, USA, catalog no. 11875085) supplemented with 10% fetal bovine serum (Invitrogen, Waltham, MA, USA, catalog no. 26140), MEM non-essential amino acids (Invitrogen, Waltham, MA, USA, catalog no. 11140050), 100 U/mL penicillin, and 100 μg/mL streptomycin (Invitrogen, Waltham, MA, USA, catalog no. 15140).

The Lf-C2 and Lf-4-2 cell lines were maintained in Fibroblast Basal Medium (ATCC, Manassas, VA, USA, catalog no. PCS-201-030) supplemented with the Fibroblast Growth Kit-Low Serum (ATCC, Manassas, VA, USA, catalog no. PCS-201-041) and 0.3 μg/mL puromycin (Gibco, Evansville, IN, USA, catalog no. A1113803).

HEK293 cells were purchased from Agilent (Santa Clara, CA, USA, catalog no. 240073) and cultured in DMEM (Thermo Fisher Scientific, Waltham, MA, USA, catalog no. 11965118) supplemented with 10% fetal bovine serum, MEM non-essential amino acids, 100 U/mL penicillin, and 100 μg/mL streptomycin.

All adherent cell lines were maintained at 37 °C in a humidified incubator with 5% carbon dioxide. Cells were passaged by washing once with Dulbecco’s phosphate-buffered saline (Thermo Fisher Scientific, Waltham, MA, USA, catalog no. 21-030-CM), dissociating with 0.25% trypsin-EDTA (Invitrogen, Waltham, MA, USA, catalog no. 25200), and resuspending in fresh complete culture medium.

### 2.2. Recombinant Lentiviral Vector Production

For lentiviral vector production, the HEK293 cells were seeded at 70–80% confluency. The cells were then transfected with fusion-targeting shRNA or control shRNA constructs, psPAX2 (in-house plasmid), and VSV-G (in-house plasmid) plasmids using JetPRIME (Polyplus Transfection, Illkirch, France, catalog no. 101000046) reagent. Following overnight incubation, the transfection medium was replaced with complete DMEM growth medium. Lentiviral vectors were collected at 48, 72, and 96 h after transfection. The collected viral supernatants were pooled and centrifuged at 1250 rpm for 5 min to remove cellular debris. The clarified supernatants were transferred into new Eppendorf tubes and stored at −80 °C until further use. Lentiviral vector titers were determined using the qPCR Lentivirus Titration Kit (Applied Biological Materials, Richmond, BC, Canada; catalog no. LV900) according to the manufacturer’s instructions.

### 2.3. Fluorescence Microscopy

Cells in complete RPMI-1640 medium were imaged at 10× magnification using an EVOS M5000 Cell Imaging System (Thermo Fisher Scientific, Waltham, MA, USA). Images were acquired using brightfield and TX Red filter sets (Chroma Technology, Bellows Falls, VT, USA). The filter settings for mKate fluorescence imaging were 579 nm excitation and 603 nm emission. Image acquisition and data processing were performed using the SlideBook 5.0 software package. All images within the same experimental group were acquired with identical exposure settings and processed with the same parameters in ImageJ software/Fiji (National Institutes of Health, Bethesda, MD, USA) to ensure consistent image analysis.

### 2.4. Flow Cytometry

For flow cytometry, cells were harvested by trypsin-EDTA dissociation, neutralized with complete medium, and centrifuged at 1000× *g* for 5 min. Cell pellets were washed once with phosphate-buffered saline (PBS) and resuspended in 0.5 mL of PBS. The cells were then analyzed on a BD LSRFortessa flow analyzer (Franklin Lakes, NJ, USA). The mKate fluorescence protein was measured with a 561 nm laser, a 610 nm emission filter, and a 610/20 nm band-pass filter. For data analysis, 100,000 events were collected, and the FlowJo software (v10.8.1; BD Biosciences, Franklin Lakes, NJ, USA) was used. An FSC (forward scatter)/SSC (side scatter) gate was generated using an untransduced INT-SFT sample and applied to all cell samples.

### 2.5. RNA Extraction and qRT-PCR Analysis

For real-time reverse transcription quantitative polymerase chain reaction (RT-qPCR) analysis, total RNA was extracted using the RNeasy Mini Kit (Qiagen, Venlo, The Netherlands, catalog no. 74104). First-strand complementary DNA (cDNA) was synthesized using the QuantiTect Reverse Transcription Kit (Qiagen, Venlo, The Netherlands, catalog no. 205311). Quantitative PCR was performed using the KAPA SYBR FAST Universal qPCR Kit (Kapa Biosystems, Wilmington, MA, USA, catalog no. KK4601), with glyceraldehyde-3-phosphate dehydrogenase (GAPDH) used as the internal reference control (Table S10). The primer sequences used for human GAPDH were: forward primer P7, 5′-AATCCCATCACCATCTTCCA-3′, and reverse primer P8, 5′-TGGACTCCACGACGTACTCA-3′. The primer sequences used for human STAT6 were: forward primer P9, 5′-AACCTTTCTCCTCCGCTTCA-3′, and reverse primer P10, 5′-TGGCTGGATGTTCTCTATCTGT-3′. The forward primer P11 for the NAB2::STAT6 fusion transcript was 5′-CGAAGCCACCTCTCGCAG-3′, and the reverse primer P12 for the NAB2::STAT6 fusion transcript was 5′-CTTGTAGTGGCTCCGGAAAG-3′. The forward primer P13 for the LAMC1 fusion transcript was 5′-CTGTGAGGTCAACCACTTTGGG-3′, and the reverse primer P14 for the LAMC1 fusion transcript was 5′-AGCCTTCTCTGCATTCACAGCG-3′. Quantitative analysis was performed using the 2^−ΔΔCt^ method. Fold-change values were reported as means with SDs.

### 2.6. CellTiter-Glo Luminescent Cell Viability Assay

The CellTiter-Glo Luminescent Cell Viability kit was purchased from Promega (Madison, WI, USA, catalog no. G7573). The cell viability assays were performed according to the manufacturer’s recommendations. INT-SFT-H and INT-SFT-L were seeded into a 96-well plate (Thermo Scientific, Waltham, MA, USA, catalog no. 237105) at 10,000 cells per well. The cells were then incubated at 37 °C for 72 h. Next, CellTiter-Glo reagent was added to each well, and the plate was incubated at room temperature for 10 min. The luminescence was then measured using an FLUOstar Omega microplate reader (BMG Labtech, Ortenberg, Germany; catalog no. 0415-0003). Data were presented as mean ± standard deviation (SD). All experiments were performed in triplicate.

### 2.7. MTT Cell Proliferation Assay

The CellTiter 96 Non-Radioactive Cell Proliferation Assay (MTT) was purchased from Promega (catalog no. G4000). The cell viability assays were performed according to the manufacturer’s recommendations. INT-SFT-H and INT-SFT-L were seeded into a 96-well plate (Thermo Scientific; catalog no. 237105) at 10,000 cells per well. The cells were then incubated at 37 °C for 72 h. After 72 h, the dye was added to each well, and the plate was incubated at 37 °C for 4 h. Next, the solubilization solution/stop mix was added to each well, and the plate was incubated at 37 °C for 1 h. The absorbance was then measured using an Azure Biosystems Ao Microplate Reader (Azure Biosystems, Dublin, CA, USA; catalog no. AC3000) at 570 nm. Data were presented as mean ± standard deviation (SD). All experiments were performed in four replicates.

### 2.8. Wound Healing Assay

The INT-SFT-H and INT-SFT-L cells were seeded onto a 6-well plate (Sigma-Aldrich, Burlington, MA, USA, catalog no. CLS3516). When cells reached confluence, the culture medium was removed, and a scratch wound was generated by vertically scraping the cell monolayer from top to bottom using a 20 μL pipette tip. The cells were gently washed twice with phosphate-buffered saline (PBS) to remove detached cells and debris. Subsequently, 2 mL of RPMI-1640 medium supplemented with 0.1 mM MEM non-essential amino acids, 100 units/mL of penicillin, 100 µg/mL of streptomycin, and 100 mM HEPES (Thermo Fisher, catalog no. 15630080) was added to each well. The mKate channel images were then acquired at 0 h immediately after scratching and again at 24 h using an EVOS M5000 Imaging System with an EVOS Onstage Incubator (Thermo Fisher Scientific, catalog no. AMF5000SV and AMC20000) at 37 °C. Imaging was performed at 10× magnification.

For quantitative analysis, four image sets were analyzed for each cell line. The wound region was manually selected from each image using Inkscape 1.3.1, and pixel-based area measurements were extracted from the selected wound region. Wound closure was quantified by calculating the change in wound area between 0 h and 24 h:

(1) $$\Delta A_{normalized}=\left(A_{0h}-A_{24h}\right)$$

where $A_{0h}$ was the wound area at 0 h and $A_{24h}$ was the wound area after 24 h. Data were presented as mean ± standard deviation (SD). Statistical comparison between INT-SFT-H and INT-SFT-L was performed using a two-tailed paired *t*-test. A *p*-value of less than 0.05 was considered statistically significant.

Alternatively, for a customized Python 3.11.0-based image segmentation workflow, paired images acquired at 0 h and 24 h were cropped in Inkscape to isolate the central scratch region, followed by contrast adjustment to improve wound boundary detection. The cropped images were converted to grayscale and segmented using pixel-intensity thresholding. Next, each binary mask was divided into 40 equal vertical columns along the scratch region. The wound fraction in each column was calculated as the ratio of wound pixels to total pixels. Local wound closure was then calculated by comparing the wound fraction at 0 h and 24 h using:

(2) $$\mathrm{Local}\;\mathrm{wound}\;\mathrm{closure}\left(\%\right)=\frac{{\mathrm{Wound}\;\mathrm{fraction}}_{0h}-{\mathrm{Wound}\;\mathrm{fraction}}_{24h}}{{\mathrm{Wound}\;\mathrm{fraction}}_{0h}}\times 100$$

Negative closure values were set to 0%, and the mean closure was calculated as the average closure across all 40 columns. Data were presented as mean ± standard deviation (SD). Finally, a statistical comparison between INT-SFT-H and INT-SFT-L was performed using a two-tailed paired *t*-test. A *p*-value of less than 0.05 was considered statistically significant.

### 2.9. RNA Sequencing (RNA-Seq)

For RNA sequencing (RNA-seq) analysis, total RNA was extracted using the RNeasy Mini Kit (Qiagen, catalog no. 74104). RNA purity was assessed based on the OD260/OD280 ratio, with acceptable values ranging from 1.8 to 2.2. Three biological replicates were analyzed for each experimental condition. RNA sequencing was performed using the Genewiz Standard RNA-Seq service (South Plainfield, NJ, USA), which required 1–2 μg of total RNA diluted to 10 μL in nuclease-free water. Libraries were sequenced on an Illumina HiSeq platform (San Diego, CA, USA) using a paired-end 2 × 150 bp configuration with single indexing, generating approximately 20 million reads per sample. All sequencing libraries achieved mean quality scores above 35. For transcriptomic analysis, reads were aligned to the human reference genome (GRCh38; https://genome-idx.s3.amazonaws.com/hisat/grch38_tran.tar.gz, accessed on 11 March 2026) using HISAT2. Transcript assembly and quantification were performed using StringTie 2.1.4, and differentially expressed genes were identified using DESeq2 1.44.0. Differentially expressed genes were defined using the following criteria: adjusted *p*-value < 0.05 and absolute log2 fold change > 1 [20].

### 2.10. Whole-Cell Lysate Preparation and Mass Spectrometry

Whole-cell lysates were prepared from INT-SFT-H and INT-SFT-L cells. Briefly, cell pellets were washed with ice-cold PBS and resuspended in 1XRIPA buffer (Cell Signaling Technology, Danvers, MA, USA, catalog no. 9806) supplemented with protease and phosphatase inhibitors (Cell Signaling Technology, catalog no. 5872). The cell suspensions were homogenized by passing 10 times through a 1 mL syringe fitted with a 30G needle (VWR, Leicestershire, UK, catalog no. 328411), followed by incubation on ice for 30 min. Samples were centrifuged at 16,000 rpm for 10 min at 4 °C, and the supernatants were collected. Protein concentrations were quantified using the Pierce BCA Protein Assay Kit (Thermo Fisher Scientific, catalog no. 23227). For downstream mass spectrometry analyses, 500 μg of total protein per sample was used, and three biological samples were included for each cell line. Mass spectrometry (MS) acquisition and primary data analysis were performed at the University of Texas Southwestern Proteomics Core facility. Raw label-free data were processed with Proteome Discoverer 3.0 and searched against the UniProt-reviewed human protein database. Subsequent bioinformatic analysis was conducted using Perseus software (version 2.0.11) [21]. Data were log2-transformed, and missing values were imputed from a normal distribution with a width of 0.3 relative to the overall data distribution and a downshift of 1.8 standard deviations. Next, statistical significance was determined using Student’s *t*-tests with a permutation-based false discovery rate (FDR) threshold of 0.05. For clustering analysis, protein abundance ratios across samples and proteins were normalized with Z-scores.

## 3. Results

### 3.1. Generation of Stable NAB2::STAT6 Knockdown Cell Lines Using shRNAs

**Figure 1:**
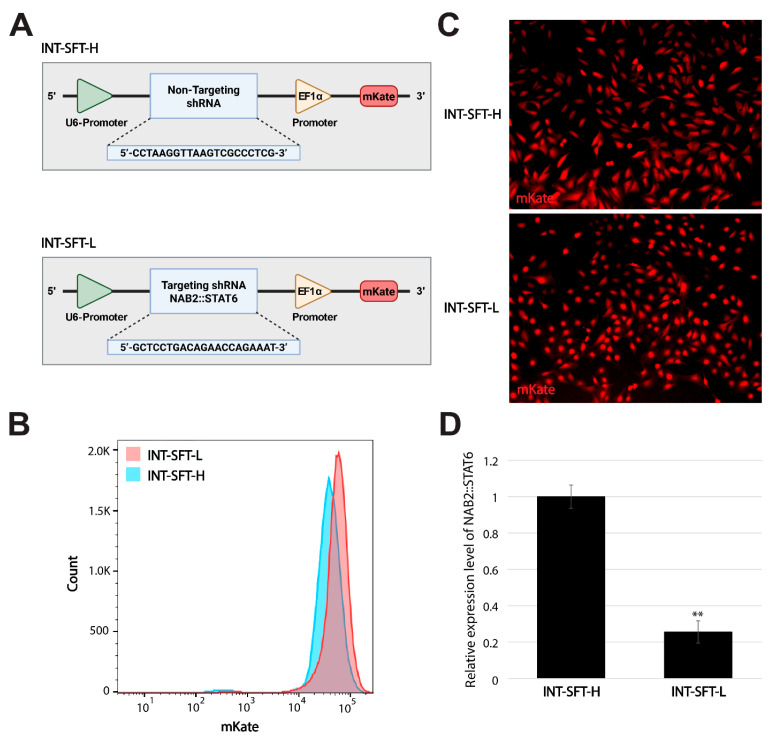
Construction of INT-SFT-H and INT-SFT-L cell lines. (**A**) Schematic representation of the lentiviral vectors for constructing INT-SFT-H and INT-SFT-L cells. (**B**) Flow cytometry assay confirmed ~100% transduction efficiencies in sorted INT-SFT-H and INT-SFT-L cells. (**C**) Fluorescence microscopy images confirmed ~100% transduction efficiencies in sorted INT-SFT-H and INT-SFT-L cells. (**D**) Quantitative RT-PCR confirmed that the relative expression of NAB2::STAT6 transcripts was 74.4% lower in INT-SFT-L compared to INT-SFT-H. (For each column, mean ± SD/Standard Deviation was displayed. ** indicated *p*-value < 0.01 using two-tailed, paired *t*-test).

We utilized the patient-derived solitary fibrous tumor cell line (INT-SFT) [17,18] as our parental cell line (NAB2::STAT6 fusion type: NAB2exon6-NAB2intron6-STAT6exon16). To investigate the functional role of the NAB2::STAT6 fusion oncoprotein, we generated two stable derivative cell lines via lentiviral transduction: one stably expressing a short hairpin RNA (shRNA) targeting the NAB2::STAT6 fusion transcript (INT-SFT-L, shRNA target sequence: 5′-GCTCCTGACAGAACCAGAAAT-3′), and a paired control line expressing a non-targeting shRNA with no homology to human transcripts (INT-SFT-H, shRNA target sequence: 5′-CCTAAGGTTAAGTCGCCCTCG-3′) (Figure 1A). Additionally, the lentiviral vector contained a mKate fluorescent reporter transcript to enable tracking of transduced cells. After two rounds of flow cytometry-based cell sorting for mKate-positive subpopulations (Figure 1B), fluorescence microscopy confirmed stable transgene integrations, with mKate signal observed in both INT-SFT-H and INT-SFT-L cells (Figure 1C). Finally, quantitative PCR (qPCR) analysis revealed a significant reduction in NAB2::STAT6 transcript levels in INT-SFT-L relative to INT-SFT-H (74.4% decrease; Figure 1D). In contrast, no significant reduction in wild-type STAT6 expression was observed (Figure S1). Taken together, our results showed that the NAB2::STAT6 transcript was efficiently and specifically suppressed in INT-SFT-L cells compared with INT-SFT-H cells.

### 3.2. Phenotypic Assays of INT-SFT-H and INT-SFT-L Cell Lines

**Figure 2:**
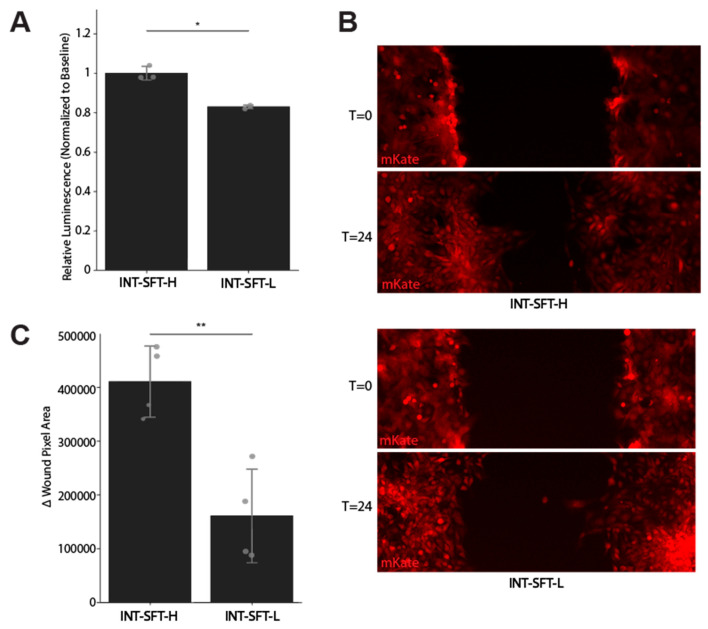
In vitro phenotypic characterization of the INT-SFT-H and INT-SFT-L cell lines. (**A**) Cell proliferation assay showed a 17.1% reduction in cell proliferation rate in INT-SFT-L compared to INT-SFT-H. (**B**) Representative images from the wound healing assay showed higher cell motility in INT-SFT-H cells than in INT-SFT-L cells. (**C**) The wound healing assay showed a 2.6-fold increase in the cell motility rate in INT-SFT-H cells compared to INT-SFT-L cells. For each column, mean ± SD/Standard Deviation was displayed. For statistical analysis, two-tailed, paired *t*-tests were used. ** indicated *p*-value < 0.01, * indicated *p*-value < 0.05.

To determine whether NAB2::STAT6 knockdown alters cellular growth and motility dynamics, we first performed a CellTiter-Glo (CTG) Luminescent Cell Viability assay across engineered cell lines. As shown in Figure 2A, the knockdown of the NAB2::STAT6 fusion transcript induced a 17.1% reduction in cell proliferation rate (*p* = 0.01). Next, to assess the fusion’s effects on cell motility, a wound healing assay was performed. The mKate channel images were used because (1) the mKate expression was detected in ~100% of both cell lines; and (2) compared to the bright field images, the wound borders in the mKate channel were more identifiable, thus minimizing downstream analysis bias using the Inkscape software (see Section 2.8, Equation (1)). As shown in Figure 2B, over the 24 h period, the extent of closure was greater in the INT-SFT-H group compared to the INT-SFT-L group. More specifically, during the experimental window, INT-SFT-H exhibited a mean wound area reduction of 410,369.48 ± 66,193.64-pixel area, whereas INT-SFT-L showed a mean reduction of 160,735.78 ± 86,936.62-pixel area (Figure 2C, ~2.6-fold greater in INT-SFT-H than that in INT-SFT-L, *p* = 0.001). Taken together, our results implied that NAB2::STAT6 fusion expression played an important role in both cell proliferation and cell motility in SFTs.

### 3.3. NAB2::STAT6 Fusion Expression Induced Extensive Transcriptional Remodeling

**Figure 3:**
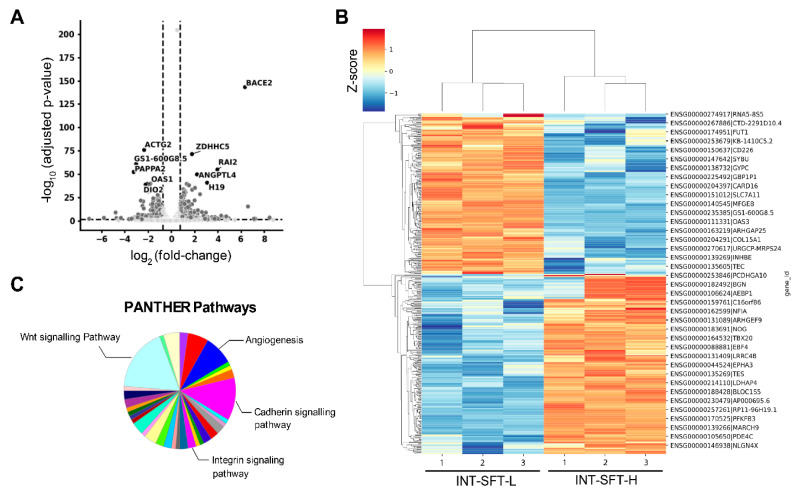
Transcriptome analysis of INT-SFT-H and INT-SFT-L cell lines. (**A**) Volcano plot of differentially expressed genes in INT-SFT-H vs. INT-SFT-L cells. The vertical dotted lines represent a 2-fold change. (**B**) Heatmap showing differentially expressed genes in INT-SFT-H vs. INT-SFT-L cells. (**C**) PANTHER analysis showed that multiple cancer-related signaling pathways were enriched in INT-SFT-H cells.

To systematically characterize the transcriptional programs induced by NAB2::STAT6 fusion expression, we performed RNA-sequencing (RNA-seq) on INT-SFT-H and INT-SFT-L cells using an Illumina HiSeq platform. Differential expression analysis identified 418 differentially expressed genes (DEGs) between the two cell lines (adjusted *p* < 0.05, |log_2_(fold-change)| > 1), demonstrating that NAB2::STAT6 fusion expression induces extensive transcriptional remodeling (Figure 3A) [15,20]. More specifically, 221 genes were significantly upregulated in INT-SFT-H cells (Table S1), including BACE2, ZDHHC5, RAI2, NLGN4X, and PDE4C, while 197 genes were downregulated (Table S2), including DIO2, OAS1, and FUT1 (Figure 3A,B). Next, since NAB2::STAT6 was hypothesized as a transcriptional activator, we performed pathway enrichment analysis using the PANTHER classification system on the subset of DEGs upregulated in INT-SFT-H (Table S1). As shown in Figure 3C and Table S3, multiple cancer-relevant pathways were significantly enriched, including integrin signaling, Wnt signaling, Cadherin signaling, and angiogenesis, highlighting the broad reprogramming of the oncogenic transcriptional landscape driven by the NAB2::STAT6 fusion in SFT cells.

### 3.4. Quantitative Proteomics Revealed NAB2::STAT6 Fusion-Induced Proteome Perturbation

**Figure 4:**
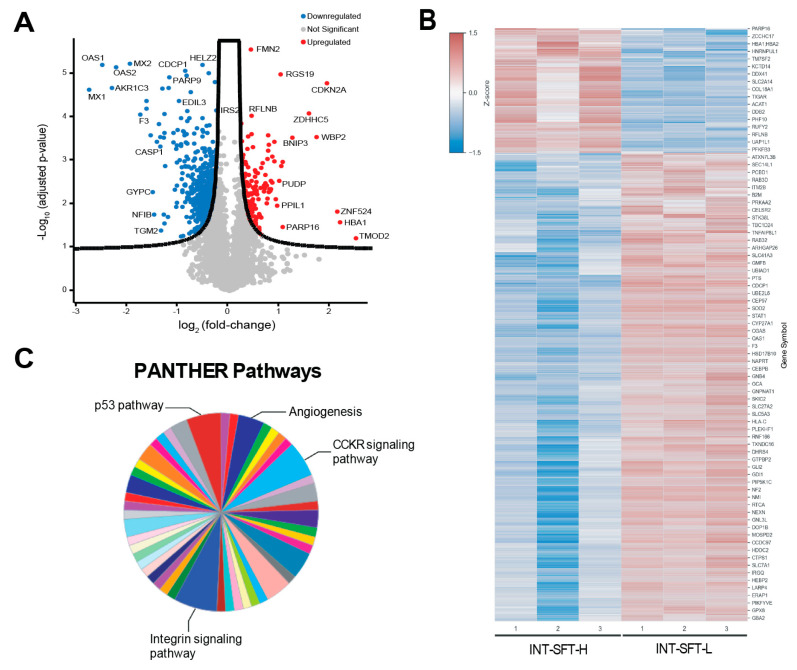
Proteomic profiling of INT-SFT-H and INT-SFT-L cells. (**A**) Volcano plot showing differentially expressed proteins in INT-SFT-L relative to the INT-SFT-H cell line. Proteins significantly upregulated in INT-SFT-H are shown in red, and proteins downregulated in INT-SFT-H are shown in blue. The black line separates the significant fold-change protein groups from the non-significant ones. (**B**) Heatmap showing differentially expressed proteins in INT-SFT-H vs. INT-SFT-L cells. (**C**) PANTHER pathway analysis showed that multiple cancer-related signaling pathways were enriched in INT-SFT-H cells.

To characterize proteome-level perturbations driven by NAB2::STAT6 fusion expression, we performed label-free quantitative mass spectrometry on whole-cell lysates from INT-SFT-H and INT-SFT-L cells. As shown in Figure 4A, 628 proteins were identified as significantly differentially expressed proteins (DEPs, permutation-based FDR < 0.05), of which 132 were upregulated (Table S4), and 496 were downregulated (Table S5) in INT-SFT-H relative to INT-SFT-L cells. More specifically, among the most prominently upregulated proteins in INT-SFT-H cells were PARP16, CDKN2A, RGS19, and ZDHHC5, whereas downregulated proteins included interferon-stimulated genes such as OAS1, OAS2, MX1, and MX2 (Figure 4A,B). Finally, as with RNA-seq, the PANTHER pathway analysis was performed using the upregulated protein targets in INT-SFT-H (Table S4), revealing enrichment of signaling pathways commonly observed in cancer tissues, including the p53 and CCKR pathways (Figure 4C, Table S6). Importantly, 15 candidate NAB2::STAT6-induced signaling pathways were observed in both RNA-seq and proteomic assays (Table S7), including the p53 pathway, angiogenesis, and the integrin signaling pathway. Taken together, our results implied that these paired, clinically relevant SFT cell lines (INT-SFT-H and INT-SFT-L) can be used to uncover potential molecular mechanisms of SFTs.

## 4. Discussion

The shRNA-mediated knockdown approach was used to construct our INT-SFT-L cell line, which showed decreased NAB2::STAT6 fusion transcript levels but not wild-type STAT6 levels (Figure 1). However, several limitations of this technique should be noted: (1) shRNA often produces incomplete gene silencing (the expression of NAB2::STAT6 fusion remained at 25.6% in INT-SFT-L compared to INT-SFT-H, Figure 1D) [22,23,24], which may leave sufficient residual gene expression to mask phenotypic effects. This may explain the relatively minor reduction in cell proliferation rate observed in INT-SFT-L (Figure 2A). (2) shRNA may induce off-target silencing, which can further confound experimental interpretation [25,26,27]. To overcome these limitations, alternative approaches such as CRISPR (clustered regularly interspaced short palindromic repeats)-based genome engineering [28,29,30] or antisense oligonucleotides (ASOs) [17] can be employed. Specifically, the CRISPR/SpCas9 system enables precise and complete NAB2::STAT6 fusion gene knockout, while ASOs provide transient, sequence-specific suppression of RNA transcripts without requiring permanent genomic modification. Together, these complementary technologies could offer more robust and flexible strategies for investigating the molecular mechanisms and therapeutic options for SFTs. Next, lentiviral transduction alone may alter gene expression and phenotypic features in cells. To address this challenge, rather than the untransduced parental INT-SFT line, we used a non-targeting control shRNA delivered via the same lentiviral vector and protocol as the negative control, ensuring that any effects of transduction itself were equally present in both conditions. In future studies, parental INT-SFT cells could be used to specifically assess the effects of the lentiviral transduction procedure.

The significant reduction in both cell proliferation and wound closure rates observed in INT-SFT-L cells (Figure 2) supported the hypothesis that NAB2::STAT6 fusion proteins play important roles in SFT cancer cell growth and invasiveness [14,31]. To confirm the observed differences in cell proliferation rates between INT-SFT-H and INT-SFT-L cells, we further performed MTT assays. As shown in Figure S2, the knockdown of NAB2::STAT6 fusion proteins induced significantly lower cell growth rates (relative cell proliferation rates: INT-SFT-H: 100.00% ± 2.74%, INT-SFT-L: 50.06% ± 4.59%). We note that the difference in observed growth rate reductions between the CTG luminescent cell viability assay (Figure 2A) and this MTT assay may be due to their distinct mechanisms: while the CTG assay quantifies ATP levels in living cells, the MTT assay measures cellular metabolic activity via mitochondrial dehydrogenases. Next, due in part to fibroblast cell morphology, the wound borders in the bright-field images of our wound-healing assays were less discernible. To ensure the accuracy of our quantification, we have developed an additional, customized Python-based image segmentation workflow (Equation (2)) to measure wound area. As shown in Figure S3, INT-SFT-H cells showed a mean wound closure of 42.3%, whereas INT-SFT-L cells showed a mean wound closure of 13.5% after 24 h. Consistent with Figure 2C, these results suggest that INT-SFT-H cells exhibit higher migratory capacity compared with INT-SFT-L cells (~3.1-fold greater in INT-SFT-H than in INT-SFT-L). Finally, additional SFT cancer-related phenotypes could be explored in future studies. For example, colony formation assays can be performed to study long-term, anchorage-independent cell growth and transformation potential. Similarly, it should be noted that both cell proliferation and wound closure assays were performed in two-dimensional (2D) platforms, which may not fully recapitulate the complexity of tumor growth and invasion within a three-dimensional (3D) microenvironment. Therefore, in future studies, more physiologically relevant assays, such as 2.5D/3D transwell and spheroid invasion assays [32,33,34], could also be included.

Our transcriptomic and proteomic analyses revealed multiple common signaling pathways (Table S7), particularly angiogenesis and integrin signaling. It is worth noting that angiogenesis plays a central role in the development and progression of SFTs, which are characterized by prominent vascular features (dense, branching “staghorn” vasculature) [35,36,37]. It was hypothesized that increased angiogenesis in SFTs supports oxygen and nutrient delivery, promotes tumor expansion, and may contribute to local invasion and metastasis in aggressive cases. Accordingly, several anti-angiogenic therapies have demonstrated clinical benefit in advanced or unresectable disease [38,39]. Similarly, LAMC1, a key component of the integrin signaling pathway [40], was identified in both RNA-seq (confirmed by quantitative RT-PCR, Figure S4) and proteomic assays. LAMC1 encodes the laminin subunit gamma-1, a key component of the extracellular matrix (ECM) that interacts with integrin receptors to regulate cell adhesion, migration, and mechanotransduction (Figure S5) [41]. It is worth noting that overexpression of LAMC1 has been reported in various cancers, suggesting alterations in cell–matrix communication that support proliferation (Figure 2A), cytoskeletal reorganization, and survival signaling in cancer cells [42,43,44,45,46,47].

Critically, we emphasize that SFTs exhibit substantial molecular, histological, and clinicopathological diversity. Genetically, at least 6 distinct fusion types have been reported, which, based on clinical outcomes, can be broadly categorized into two subtypes represented by NAB2exon4::STAT6exon2/4 and NAB2exon6::STAT6exon16/17 fusions (the fusion type of INT-SFT belongs to the NAB2exon6::STAT6exon16/17 category) [48]. Recently, we prepared human lung fibroblast-based engineered SFT cell lines (Lf-4-2: expressing the NAB2exon4::STAT6exon2 fusion transcript; Lf-C2: expressing a control NAB2exon2 transcript) and performed RNA-seq [49]. As shown in Figure S6, the transcriptomic landscapes differed significantly between these two fusion types (Tables S8 and S9). Of the 221 candidate genes upregulated in INT-SFT-H compared to INT-SFT-L, only 22 were also upregulated in Lf-4-2 compared to Lf-C2 (Figure S6A, Table S8). Similarly, of the 197 candidate genes downregulated in INT-SFT-H compared to INT-SFT-L, only 5 were also downregulated in Lf-4-2 compared to Lf-C2 (Figure S6B, Table S9). More specifically, while the LAMC1 expression was upregulated in INT-SFT-H vs. INT-SFT-L (Figure S2), its expression was suppressed by ~23% in Lf-4-2 vs. Lf-C2 (Figure S7). Taken together, our results implied that NAB2exon4::STAT6exon2/4 and NAB2exon6::STAT6exon16/17 fusions may function as distinct transcriptional activators and regulate the expression levels of different gene targets.

In summary, our paired patient-tissue-derived SFT cell models (INT-SFT-H and INT-SFT-L) provide a biologically matched comparative model and could serve as valuable research resources for studying etiology and exploring novel therapeutics for SFTs with NAB2exon6::STAT6exon16/17 fusion types.

## 5. Conclusions

Our study established genetically matched SFT cell models with differential expression of the NAB2::STAT6 fusion transcript, providing a robust platform for investigating the molecular mechanisms underlying SFT pathogenesis. These findings advance our understanding of SFT biology and provide a valuable preclinical resource for identifying and evaluating novel therapeutic targets for this rare mesenchymal malignancy.

## Data Availability

The original contributions presented in this study are included in the article and Supplementary Materials. Further inquiries can be directed to the corresponding author.

## Supplementary Materials

The following supporting information can be downloaded at: https://www.mdpi.com/article/10.3390/cancers18152385/s1.
