# Supplementary material for: Construction and Characterization of Paired Patient-Derived Cell Models for Solitary Fibrous Tumors (SFTs)
Source: Cancers (Basel). 2026 Jul 24;18(15):2385. doi: 10.3390/cancers18152385 (PMC13465001; doi:10.3390/cancers18152385)
Supplement: Supplementary file 1 [file cancers-18-02385-s001.zip › cancers-4403445-supplementary.pdf]

Supplemental Materials

General cloning protocols

The Q5 High-Fidelity 2X Master Mix (New England Biolabs, catalog number: M0492) was used for all polymerase chain reactions (PCR) according to the manufacturer's protocol. All oligonucleotides were ordered from Sigma-Aldrich and were listed in **Table S8**. The plasmids were constructed using PCR amplification, restriction enzyme digestion (all restriction enzymes were ordered from New England Biolabs), and ligation with T4 DNA ligase (New England Biolabs, catalog number: M0202). Gel purification and PCR purification were performed with QIAquick Gel Extraction kit (Qiagen, catalog number: 28707) and PCR Purification kit (Qiagen, catalog number: 28104). Transformations were performed using NEB 5-alpha electrocompetent *Escherichia Coli* cells (New England Biolabs, catalog number: C2987). The minipreps were performed using the QIAprep Spin Miniprep Kit (Qiagen, catalog number: 27106). The final plasmids were confirmed by restriction enzyme digestions and direct Sanger sequencing.

DNA constructs

EF1a-mKate: A 740 bp fragment was PCR amplified from the parental plasmid new_NS12.4 (unpublished data) using primers P1 and P2, introducing EcoRI and MluI restriction sites, respectively. The PCR product was digested with EcoRI and MluI and ligated into the EcoRI/MluI-digested new_NS12.4 backbone using T4 DNA ligase. The ligation mixture was transformed into chemically competent *Escherichia coli* DH5α cells, and transformants were selected on LB agar plates containing carbenicillin (100 μg/mL).

EF1a-mKate-U6-shRNA-negativeControl: A 330 bp non-targeting shRNA cassette was PCR amplified from the D15 plasmid (unpublished data) using primers P3 and P4, introducing SphI restriction sites. The PCR product was digested with SphI and ligated into the SphI-linearized EF1a-mKate backbone. Prior to ligation, the backbone was treated with calf intestinal phosphatase (CIP) to prevent self-ligation. Following transformation into DH5α competent cells, colonies were selected on carbenicillin-containing LB agar plates.

EF1a-mKate-U6-shRNA-NAB2STAT6: An shRNA targeting the INT::SFT fusion junction (target sequence: 5′-GCTCCTGACAGAACCAGAAAT-3′) was designed using VectorBuilder (VectorBuilder Inc.). A 450 bp fragment containing the NAB2::STAT6-specific shRNA cassette w as P CR a mplified f rom E F1a-mKate-U6-shRNA-negativeControl using primers P5 and P6, introducing ClaI and XbaI restriction sites. The PCR product was digested with ClaI and XbaI and ligated into the ClaI/XbaI-digested EF1a-mKate-U6-shRNA-negativeControl backbone using T4 DNA ligase. The ligation mixture was transformed into DH5α competent cells, and transformants were selected on carbenicillin-containing plates.

All intermediate and final constructs were screened by diagnostic restriction enzyme digestion and verified by whole-plasmid sequencing (GENEWIZ) to confirm sequence integrity, correct insert orientation, and absence of unintended mutations.

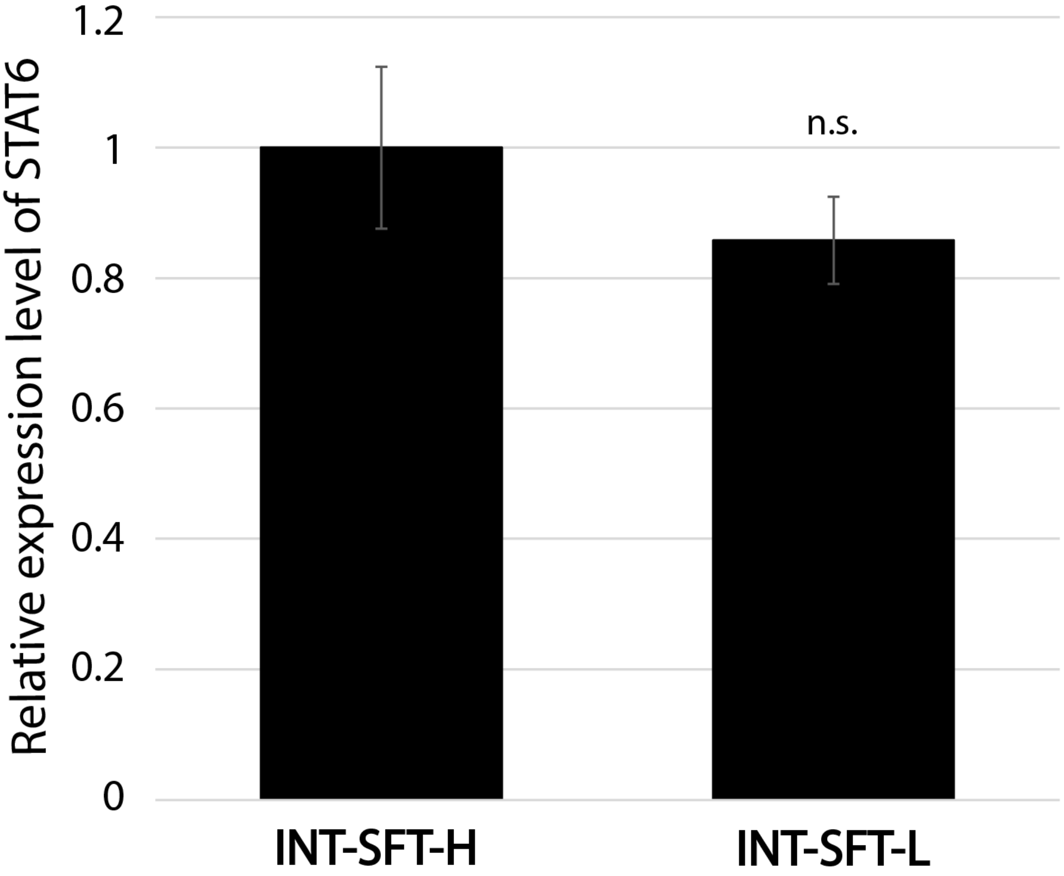

Figure S1. No significant reduction of the expression of wild-type STAT6 was observed in INT-SFT-L compared to INT-SFT-H. For each column, mean ± SD/Standard Deviation was displayed. For statistical analysis, a two-tailed, paired t-test was performed. n.s. denotes no statistically significant difference.

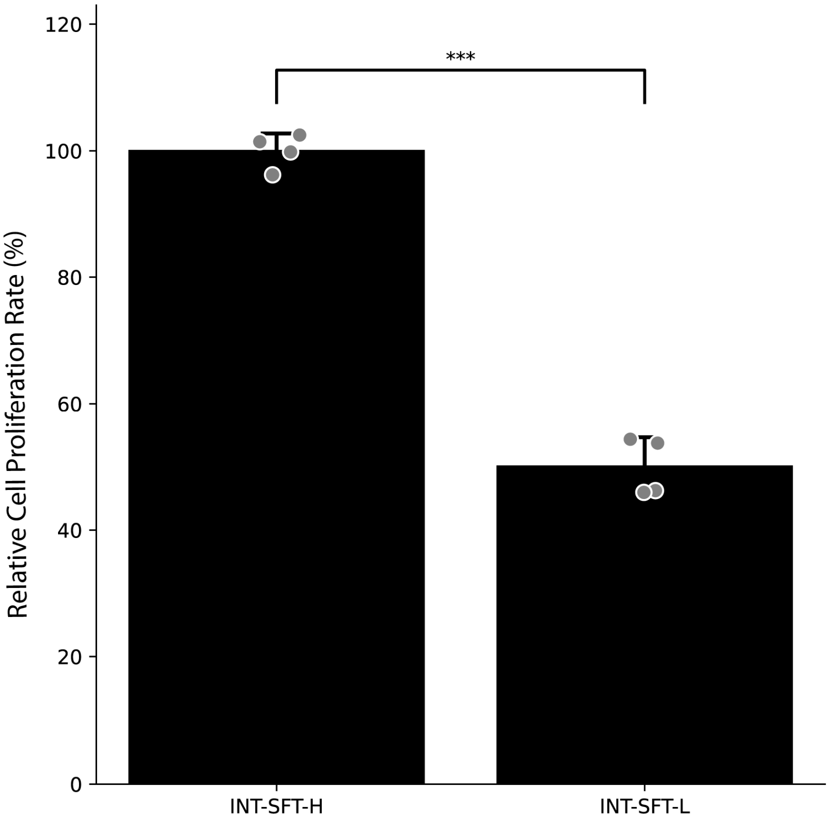

Figure S2. MTT cell proliferation assay of the INT-SFT-H and INT-SFT-L cell lines. The knockdown of NAB2::STAT6 fusion proteins resulted in significantly lower cell growth rates (relative cell proliferation rates: INT-SFT-H: 100.00% ± 2.74%, INT-SFT-L: 50.06% ± 4.59%). For each column, mean ± SD/Standard Deviation was displayed. For statistical analysis, two-tailed, paired t-tests were used. *** indicated p-value < 0.001.

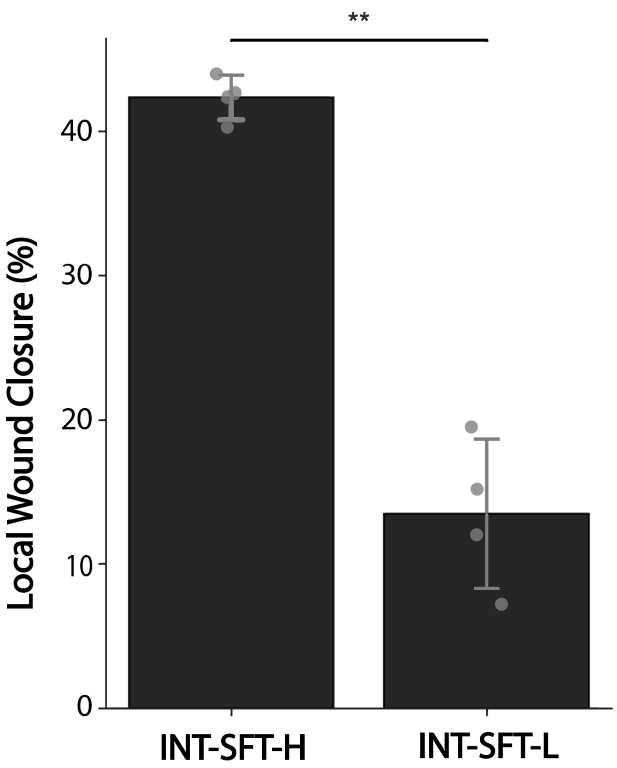

Figure S3. In vitro phenotypic characterization of the INT-SFT-H and INT-SFT-L cell lines. The wound healing assay was quantified using a customized Python-based image segmentation workflow and showed a 3.1-fold increase in cell motility in INT-SFT-H cells compared with INT-SFT-L cells. For each column, mean ± SD/Standard Deviation was displayed. For statistical analysis, two-tailed, paired t-tests were used. ** indicated p-value < 0.01.

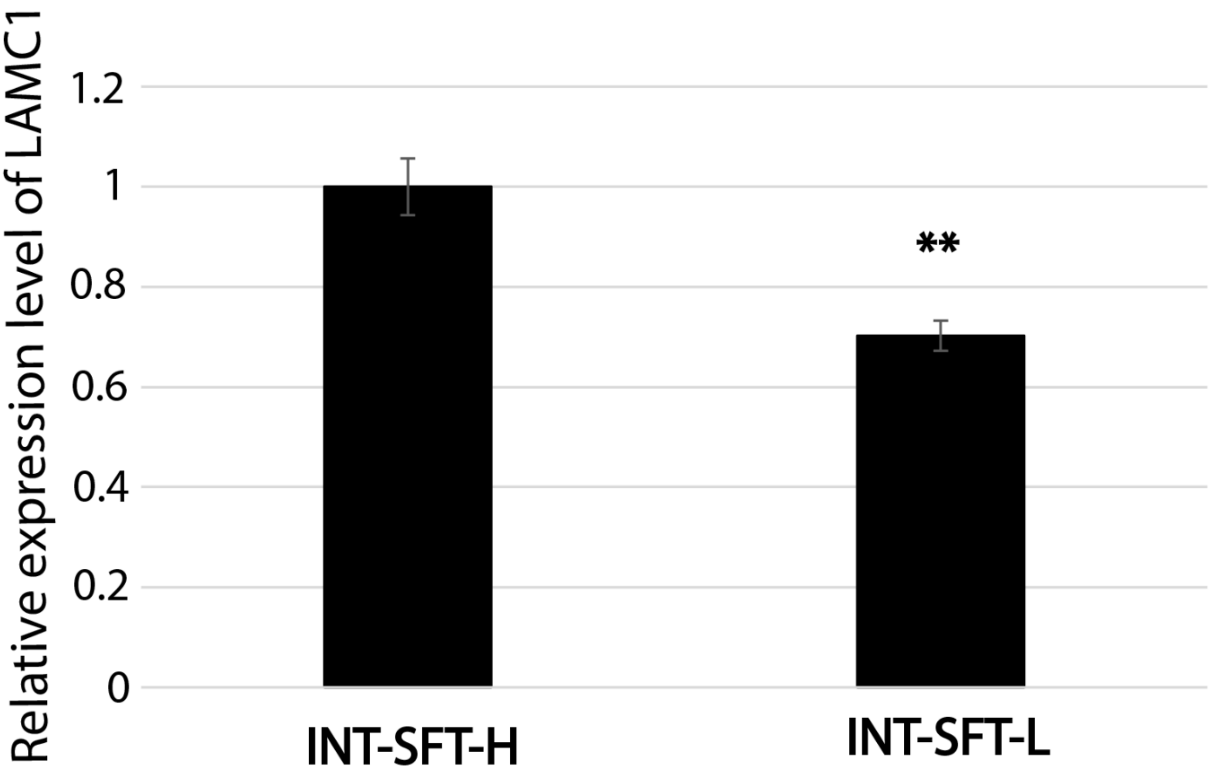

Figure S4. The expression of LAMC1 was significantly suppressed (30% reduction) in INT-SFT-L cells compared to INT-SFT-H. For each column, mean ± SD/Standard Deviation was displayed. For statistical analysis, a two-tailed, paired t-test was performed. ** denotes p-value < 0.01.

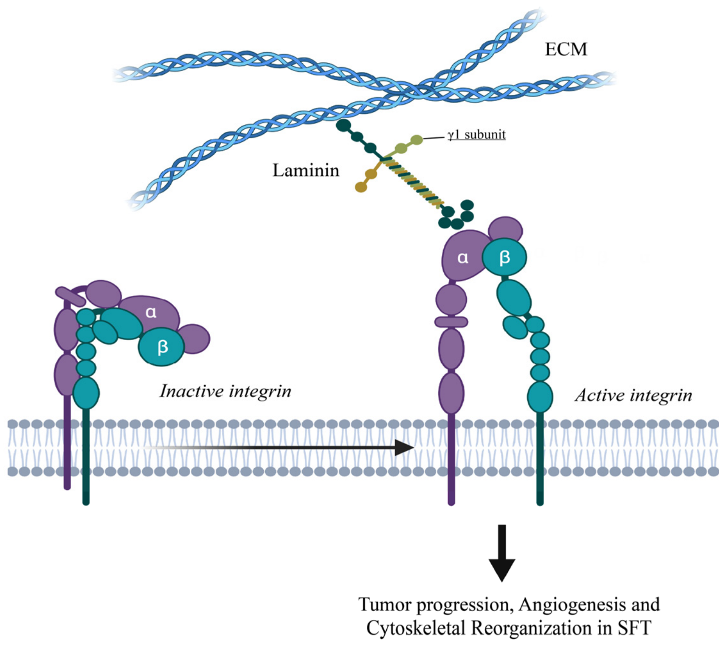

Figure S5. Proposed mechanism of upregulation of the laminin-integrin signaling in SFT pathogenesis. Created with BioRender.com. We hypothesized that the observed overexpression of LAMC1 in INT-SFT-H cells may support greater laminin assembly and ECM deposition, thereby enhancing integrin activation and driving tumor progression, angiogenesis, and cytoskeletal reorganization in SFT.

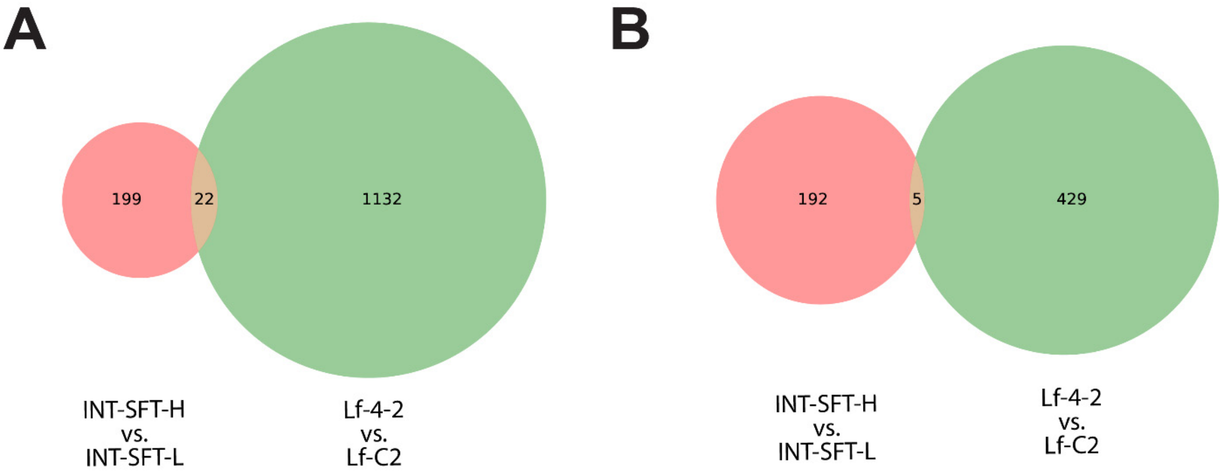

Figure S6. Comparisons of RNA-seq results between INT-SFT-H vs. INT-SFT-L (NAB2exon6::STAT6exon16/17 category) and Lf-4-2 vs. Lf-C2 (NAB2exon4::STAT6exon2 category). (A) Of the 221 candidate genes upregulated in INT-SFT-H compared to INT-SFT-L, only 22 were also upregulated in Lf-4-2 compared to Lf-C2. **(B)** Of the 197 candidate genes downregulated in INT-SFT-H compared to INT-SFT-L, only 5 were also downregulated in Lf-4-2 compared to Lf-C2.

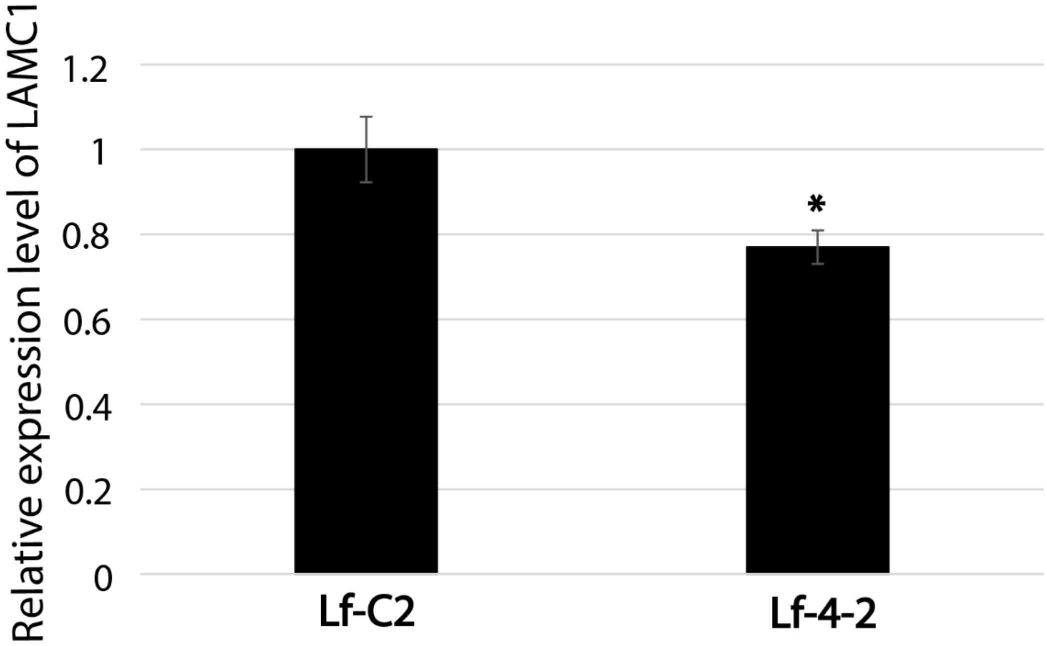

Figure S7. The expression of LAMC1 was significantly suppressed (23% reduction) in Lf-4-2 cells compared to Lf-C2. For each column, mean ± SD/Standard Deviation was displayed. For statistical analysis, a two-tailed, paired t-test was performed. * denotes p-value < 0.05.

Table S1. Upregulated genes in INT-SFT-H cells compared to INT-SFT-L cells from RNA-seq. Column description: gene_id: Ensembl gene ID number; **baseMean:** The average of the normalized

count values for the gene across all samples in both conditions; **log2FoldChange:** The estimated effect size between the two groups being compared; **pvalue:** The unadjusted p-value associated with the Wald statistic; **padj:** The p-value adjusted for multiple testing using the Benjamini-Hochberg (BH) procedure.

gene_id	baseMean	log2Fold-Change	pvalue	padj
ENSG00000171435|KSR2	31.05036228	8.470320366	2.86E-05	0.000668538
ENSG00000129910|CDH15	13.25626136	7.242357444	0.000911933	0.011415489
ENSG00000267577|CTD-2587H24.5	12.22296971	7.127321344	0.000976433	0.011982562
ENSG00000071073|MGAT4A	78.51668471	6.610096014	3.64E-18	7.95E-16
ENSG00000182240|BACE2	847.399033	6.353820247	7.72E-148	1.35E-143
ENSG00000180660|MAB21L1	13.57379778	4.951173492	0.000266977	0.004218279
ENSG00000136267|DGKB	27.16813194	4.733941369	9.94E-07	3.74E-05
ENSG00000131409|LRRC4B	15.30307126	4.733269132	0.000326074	0.005002762
ENSG00000182040|USH1G	12.99833956	4.672720648	0.00026166	0.004145527
ENSG00000265107|GJA5	20.70430577	4.592532423	2.58E-05	0.000612697
ENSG00000125965|GDF5	57.81509608	4.306827237	1.08E-07	5.22E-06
ENSG00000169515|CCDC8	12.05975688	4.106875736	0.002038947	0.021208692
ENSG00000131831|RAI2	330.1848382	3.975574398	7.95E-59	2.78E-55
ENSG00000113209|PCDHB5	22.96039971	3.971061318	0.000520594	0.007372266
ENSG00000018625|ATP1A2	254.8087497	3.894230253	0.000349892	0.005283497
ENSG00000267013|CTD-2171N6.1	12.25746056	3.783250655	0.002167633	0.022224836
ENSG00000165828|PRAP1	12.22908746	3.775523393	0.002877428	0.027507142
ENSG00000146938|NLGN4X	41.59083426	3.771040192	7.32E-06	0.00021428
ENSG00000076716|GPC4	205.4952356	3.736573191	0.000868581	0.010987435
ENSG00000135406|PRPH	103.3793938	3.66241924	0.002168432	0.022224836
ENSG00000067445|TRO	20.95972786	3.629588576	0.000350859	0.005286277
ENSG00000188620|HMX3	23.12997951	3.588823271	4.07E-05	0.000891729
ENSG00000253846|PCDHGA10	54.64573533	3.508997799	7.56E-06	0.000220155
ENSG00000114200|BCHE	22.60526357	3.481759473	0.000493004	0.007061671
ENSG00000120324|PCDHB10	16.14294455	3.385458813	0.000290422	0.004523277
ENSG00000269371|CTD-2207O23.11	14.89772115	3.33180576	0.006241822	0.049451044
ENSG00000249352|RP11-141O11.2	15.24286299	3.224535415	0.000981983	0.012033767
ENSG00000140450|ARRDC4	41.61353701	3.174614884	1.40E-05	0.000364954
ENSG00000272896|RP11-216L13.17	16.7753642	3.143698763	0.004893716	0.041148198
ENSG00000082397|EPB41L3	25.23079338	3.097421929	0.000773184	0.010001029
ENSG00000130600|H19	459.9187519	3.092248986	1.52E-44	3.33E-41
ENSG00000060718|COL11A1	194.9827195	3.055244666	0.000660694	0.008881254
ENSG00000272788|RP11-674N23.4	12.37470329	3.050169299	0.005937027	0.047613374
ENSG00000227051|C14orf132	25.20510749	3.010799228	6.40E-05	0.001319361
ENSG00000179761|PIPOX	26.82271223	2.995865582	3.66E-05	0.000818805
ENSG00000187800|PEAR1	78.70647816	2.987307096	3.12E-11	3.08E-09
ENSG00000089472|HEPH	28.70409277	2.971975986	0.00087548	0.011043224
ENSG00000278246|AC004556.2	15.53226533	2.910516339	0.001093962	0.013013609

ENSG00000121570|DPPA4	26.06835571	2.871828979	0.000820295	0.010486208
ENSG00000181449|SOX2	50.52480205	2.81704324	4.33E-05	0.00093776
ENSG00000118785|SPP1	59.56266233	2.811125432	5.05E-05	0.00108165
ENSG00000071242|RPS6KA2	118.0721359	2.788964366	2.83E-07	1.25E-05
ENSG00000269881|AC004754.3	31.04563792	2.780214257	0.004295292	0.037213795
ENSG00000034053|APBA2	18.00126246	2.731240337	0.003295094	0.030547359
ENSG00000155511|GRIA1	33.43108238	2.730728501	0.00078199	0.010092522
ENSG00000189212|DPY19L2P1	102.4766286	2.646221609	7.30E-12	8.23E-10
ENSG00000261279|ULK4P1	15.93106375	2.6183678	0.003876232	0.03452454
ENSG00000120278|PLEKHG1	28.02639839	2.602081705	0.004959929	0.041411738
ENSG00000186994|KANK3	32.05355012	2.535249217	0.00023535	0.003787062
ENSG00000271741|RP11-244H3.4	148.7129364	2.495150883	0.002534021	0.024919535
ENSG00000143847|PPFIA4	377.8305753	2.483236488	9.76E-32	5.88E-29
ENSG00000159217|IGF2BP1	30.1798065	2.47592624	9.12E-05	0.001776167
ENSG00000087085|ACHE	29.04668258	2.474884848	0.003556037	0.032421805
ENSG00000174807|CD248	40.11304064	2.335377882	0.002872123	0.027478957
ENSG00000164532|TBX20	39.51826558	2.328174123	4.73E-05	0.001019599
ENSG00000168672|FAM84B	172.9002609	2.30175059	5.70E-14	8.23E-12
ENSG00000248235|AC037459.4	31.95464234	2.293033131	0.000705251	0.009287308
ENSG00000162599|NFIA	192.0639564	2.255495889	1.03E-09	7.53E-08
ENSG00000124440|HIF3A	41.50059465	2.253060517	0.00226524	0.02292685
ENSG00000176887|SOX11	32.99354236	2.226029031	0.001458173	0.016323877
ENSG00000167772|ANGPTL4	1485.104758	2.225664653	1.32E-53	3.30E-50
ENSG00000107485|GATA3	177.2576772	2.196303301	5.95E-13	7.59E-11
ENSG00000139266|MARCH9	53.96144825	2.187779552	2.97E-06	9.70E-05
ENSG00000069812|HES2	40.99427209	2.132736096	0.001216923	0.014120672
ENSG00000180815|MAP3K15	55.74105351	2.111942469	0.000139991	0.002516822
ENSG00000131095|GFAP	92.68387166	2.079461158	3.49E-05	0.000784982
ENSG00000107159|CA9	1678.214707	2.051554997	6.96E-39	8.10E-36
ENSG00000143195|ILDR2	216.952313	2.021065473	3.84E-13	5.13E-11
ENSG00000226763|SRRM5	60.15577645	2.020774391	0.004639908	0.039485073
ENSG00000242147|RP13-463N16.6	484.7377552	2.01930454	6.38E-25	2.54E-22
ENSG00000132793|LPIN3	59.04736326	2.004988952	0.000116103	0.002167637
ENSG00000099284|H2AFY2	76.33067606	2.003417677	1.42E-06	5.14E-05
ENSG00000164283|ESM1	271.8297192	1.980857907	1.18E-11	1.28E-09
ENSG00000149294|NCAM1	49.06445719	1.976874577	0.001551786	0.017228375
ENSG00000154556|SORBS2	58.68262109	1.970713243	0.000651801	0.008788748
ENSG00000240764|PCDHGC5	90.39613534	1.932593453	0.000429356	0.006278652
ENSG00000006042|TMEM98	32.06950486	1.921703741	0.006221176	0.049414889
ENSG00000151640|DPYSL4	147.1071669	1.921014315	4.56E-11	4.33E-09
ENSG00000231890|DARS-AS1	80.45916717	1.917514489	4.13E-06	0.00012943
ENSG00000106624|AEBP1	232.7372702	1.91199931	0.004754598	0.040255133
ENSG00000185630|PBX1	55.40575032	1.870034182	0.000338469	0.005143254
ENSG00000171044|XKR6	45.36987758	1.865977184	0.001345543	0.01528827
ENSG00000162490|DRAXIN	129.6969674	1.860426153	5.27E-08	2.77E-06
ENSG00000118971|CCND2	51.52082232	1.842035806	0.00068296	0.009071685
ENSG00000105650|PDE4C	32.30799693	1.823702787	0.003816135	0.034145907
ENSG00000131089|ARHGEF9	49.97313462	1.819610618	0.000422309	0.006211989

ENSG00000124772|CPNE5	63.24291209	1.80298903	0.002222567	0.022620481
ENSG00000247095|MIR210HG	853.6246002	1.80288802	1.05E-36	9.81E-34
ENSG00000238266|LINC00707	405.2580181	1.795533574	1.23E-20	3.41E-18
ENSG00000156599|ZDHHC5	5151.289807	1.789727818	1.05E-75	6.11E-72
ENSG00000006016|CRLF1	181.1959285	1.743115475	9.69E-08	4.77E-06
ENSG00000182492|BGN	182.5278701	1.693321862	1.47E-05	0.000378821
ENSG00000270641|TSIX	98.63938482	1.692650887	2.52E-05	0.000600931
ENSG00000064692|SNCAIP	210.9559957	1.690796646	8.08E-12	9.05E-10
ENSG00000159339|PADI4	37.50509241	1.684635791	0.003544607	0.032345697
ENSG00000253882|RP11-61L23.2	222.8011891	1.683853066	3.27E-11	3.19E-09
ENSG00000137975|CLCA2	212.4079578	1.679730028	1.59E-07	7.49E-06
ENSG00000110427|KIAA1549L	384.966693	1.6621483	2.47E-14	3.84E-12
ENSG00000115297|TLX2	90.50366762	1.62833537	2.88E-05	0.000670943
ENSG00000133874|RNF122	136.9397088	1.622279737	2.02E-07	9.23E-06
ENSG00000112559|MDFI	39.60338926	1.621960777	0.00411849	0.03609359
ENSG00000107317|PTGDS	76.37036547	1.592965499	0.004900109	0.041148198
ENSG00000044524|EPHA3	292.1760631	1.592175249	1.82E-11	1.91E-09
ENSG00000251661|RP11-326C3.11	56.13850467	1.583336665	0.002638822	0.025647062
ENSG00000236090|LDHAP3	63.39761408	1.579745394	0.000466153	0.006750521
ENSG00000132386|SERPINF1	168.3068091	1.578603213	0.000103495	0.001972269
ENSG00000177283|FZD8	2018.584031	1.575166751	1.05E-18	2.43E-16
ENSG00000163132|MSX1	63.2577854	1.574126243	0.000349497	0.005283273
ENSG00000281186|LINC00706	76.85322388	1.573760662	0.00013057	0.002381752
ENSG00000145423|SFRP2	117.9293357	1.570430791	0.000275311	0.0043226
ENSG00000170745|KCNS3	245.2234353	1.5586217	9.75E-11	8.83E-09
ENSG00000257261|RP11-96H19.1	49.96360265	1.556813696	0.000888148	0.011173784
ENSG00000135269|TES	46.15204664	1.548822448	0.003081437	0.028984535
ENSG00000110042|DTX4	59.77661907	1.542715143	0.001909039	0.020120903
ENSG00000196104|SPOCK3	115.9331082	1.538087228	8.85E-06	0.000251398
ENSG00000272449|RP3-395M20.12	56.71046032	1.537410225	0.000957726	0.011836579
ENSG00000132471|WBP2	7350.186356	1.534034754	1.31E-41	1.64E-38
ENSG00000141934|PLPP2	100.8289738	1.526274007	5.59E-05	0.001182253
ENSG00000080561|MID2	78.14858582	1.526152731	5.83E-05	0.001225301
ENSG00000130021|PUDP	1028.614192	1.501813233	8.96E-24	3.07E-21
ENSG00000088881|EBF4	674.4335418	1.479162472	3.80E-11	3.67E-09
ENSG00000228288|PCAT6	61.13005382	1.47277087	0.002747515	0.026541083
ENSG00000165821|SALL2	116.5508179	1.469539696	8.06E-06	0.00023281
ENSG00000167617|CDC42EP5	57.91397261	1.466015942	0.001184205	0.013851395
ENSG00000196611|MMP1	170.9932671	1.453290433	1.40E-07	6.61E-06
ENSG00000135439|AGAP2	382.9033171	1.443154192	3.94E-14	5.88E-12
ENSG00000103381|CPPED1	194.7734275	1.438399644	6.28E-08	3.24E-06
ENSG00000188428|BLOC1S5	721.9053735	1.438174513	2.21E-17	4.39E-15
ENSG00000177614|PGBD5	165.1547017	1.437274556	7.21E-07	2.87E-05
ENSG00000184986|TMEM121	110.2290677	1.414222989	0.000676901	0.009036557
ENSG00000100100|PIK3IP1	215.7112089	1.405079431	3.01E-08	1.68E-06
ENSG00000106327|TFR2	49.35060117	1.39811707	0.003012811	0.02853597
ENSG00000153944|MSI2	74.55209471	1.386903624	0.00173198	0.018806839
ENSG00000183691|NOG	254.4822674	1.382355315	9.32E-06	0.000261977

ENSG00000149054 | ZNF215	151.8467517	1.360679184	1.93E-05	0.000479457
ENSG00000176171 | BNIP3	11028.51311	1.341588266	3.36E-43	5.49E-40
ENSG00000225205 | AC093818.1	102.1842317	1.336425904	0.000499417	0.007130159
ENSG00000182118 | FAM89A	66.54462518	1.330906686	0.003309996	0.03064771
ENSG00000184937 | WT1	104.4076781	1.330369511	0.000266685	0.004217486
ENSG00000159761 | C16orf86	75.81536045	1.328998796	0.004134298	0.036195823
ENSG00000159860 | TCAF2P1	96.19847016	1.325645969	0.000523006	0.007382501
ENSG00000166396 | SERPINB7	3434.215138	1.323221848	1.79E-23	6.00E-21
ENSG00000270006 | RP11-178L8.7	55.00183178	1.320876128	0.002803839	0.026936278
ENSG00000161544 | CYGB	60.03015193	1.315697205	0.004959044	0.041411738
ENSG00000271888 | RP11-560J1.2	51.92776592	1.311859294	0.005515415	0.045055224
ENSG00000177453 | NIM1K	50.51781499	1.310628733	0.005217319	0.043107636
ENSG00000059804 | SLC2A3	995.6952423	1.300399078	2.43E-18	5.45E-16
ENSG00000179428 | AC073072.5	134.9739427	1.294674424	0.000156608	0.002731267
ENSG00000133138 | TBC1D8B	88.13286	1.291619453	0.004498167	0.038626769
ENSG00000143816 | WNT9A	111.7889723	1.29158049	0.000676618	0.009036557
ENSG00000171700 | RGS19	940.8236258	1.291026043	2.97E-16	5.41E-14
ENSG00000162981 | FAM84A	397.408945	1.290658894	9.57E-12	1.05E-09
ENSG00000230479 | AP000695.6	58.11775754	1.289592001	0.002975864	0.028237596
ENSG00000065717 | TLE2	651.9998237	1.268760654	1.84E-10	1.56E-08
ENSG00000167779 | IGFBP6	1434.968464	1.245406605	2.58E-19	6.45E-17
ENSG00000178977 | LINC00324	107.5035151	1.241247011	0.000245682	0.003924405
ENSG00000108947 | EFNB3	1239.113066	1.239427523	1.99E-16	3.70E-14
ENSG00000134333 | LDHA	99396.70296	1.236884896	1.45E-33	9.75E-31
ENSG00000096060 | FKBP5	812.120354	1.229978415	2.59E-12	3.18E-10
ENSG00000170379 | TCAF2	313.1040677	1.228156006	6.71E-08	3.42E-06
ENSG00000145526 | CDH18	74.56257059	1.225056266	0.003401493	0.031317747
ENSG00000184292 | TACSTD2	1079.912211	1.223176128	2.36E-18	5.35E-16
ENSG00000061656 | SPAG4	816.4230418	1.216310819	1.98E-13	2.70E-11
ENSG00000139200 | PIANP	728.6482625	1.212824696	1.13E-10	1.00E-08
ENSG00000111319 | SCNN1A	163.40918	1.206849648	0.000440585	0.006426735
ENSG00000129757 | CDKN1C	130.9454216	1.205011069	0.000766802	0.009935188
ENSG00000197358 | BNIP3P1	79.44848911	1.204791551	0.002517556	0.02479949
ENSG00000214110 | LDHAP4	298.8415374	1.193154558	1.25E-07	5.96E-06
ENSG00000170525 | PFKFB3	7977.406898	1.186506999	2.31E-35	1.75E-32
ENSG00000010310 | GIPR	118.5196424	1.18468097	0.000556969	0.007755404
ENSG00000164849 | GPR146	137.2513454	1.184495356	0.00055586	0.007752313
ENSG00000105989 | WNT2	430.6583301	1.18303459	1.35E-10	1.18E-08
ENSG00000223784 | RP11-554I8.2	212.6045913	1.178124285	5.46E-06	0.00016521
ENSG00000067141 | NEO1	271.3119514	1.163280657	4.50E-06	0.000138583
ENSG00000173530 | TNFRSF10D	1187.878142	1.159914417	4.30E-21	1.27E-18
ENSG00000159388 | BTG2	249.9700272	1.156238143	8.61E-07	3.34E-05
ENSG00000187243 | MAGED4B	155.6348305	1.153866042	3.18E-05	0.000722833
ENSG00000159784 | FAM131B	425.7638625	1.151087287	2.60E-09	1.76E-07
ENSG00000186918 | ZNF395	4325.680534	1.150682456	6.21E-32	3.87E-29
ENSG00000132026 | RTBDN	104.3815972	1.143158573	0.005270526	0.043465051
ENSG00000088340 | FER1L4	1022.239492	1.136496375	1.49E-13	2.05E-11
ENSG00000154545 | MAGED4	150.3570701	1.132568777	0.000107272	0.002030962
ENSG00000104332 | SFRP1	3182.343977	1.129921806	1.43E-30	7.83E-28

ENSG00000130513|GDF15	640.3689643	1.12338193	1.73E-09	1.21E-07
ENSG00000146674|IGFBP3	7447.733274	1.122766583	1.06E-34	7.41E-32
ENSG00000106031|HOXA13	585.8053823	1.121327827	2.24E-11	2.27E-09
ENSG00000124762|CDKN1A	15689.77957	1.112302438	6.81E-31	3.84E-28
ENSG00000152229|PSTPIP2	769.9265901	1.098197327	1.24E-11	1.33E-09
ENSG00000173376|NDNF	136.8193826	1.091747177	0.00133747	0.015226245
ENSG00000175352|NRIP3	456.5731287	1.089942369	2.71E-08	1.53E-06
ENSG00000104419|NDRG1	13256.39933	1.081360087	2.44E-33	1.58E-30
ENSG00000143590|EFNA3	208.8087249	1.079554263	4.25E-05	0.00092569
ENSG00000183248|PRR36	350.8559061	1.079392441	7.74E-07	3.05E-05
ENSG00000185522|LMNTD2	216.6526399	1.070431903	0.000584756	0.008046149
ENSG00000233818|AP000695.4	81.77190457	1.068766461	0.004252249	0.036999167
ENSG00000127561|SYNGR3	326.4666895	1.065235938	1.45E-07	6.87E-06
ENSG00000170873|MTSS1	124.9874172	1.065070426	0.001968472	0.020622935
ENSG00000182580|EPHB3	136.0659425	1.062929154	0.00067791	0.009043118
ENSG00000134107|BHLHE40	3752.048604	1.06057945	3.62E-28	1.76E-25
ENSG00000129226|CD68	2001.354942	1.058844552	9.41E-13	1.17E-10
ENSG00000198715|GLMP	859.8061487	1.057922861	2.17E-11	2.21E-09
ENSG00000135862|LAMC1	17085.37477	1.055872354	2.82E-37	2.90E-34
ENSG00000141314|RHBDL3	257.0839577	1.054016883	7.82E-05	0.001570902
ENSG00000197461|PDGFA	146.460503	1.052806138	0.000295225	0.004589913
ENSG00000142252|GEMIN7	836.9326953	1.050918512	8.80E-11	8.01E-09
ENSG00000146205|ANO7	536.8909694	1.046792248	9.08E-08	4.51E-06
ENSG00000154310|TNIK	679.6618769	1.038504175	1.05E-10	9.40E-09
ENSG00000218426|RP11-475C16.1	320.0316664	1.034319605	1.27E-06	4.64E-05
ENSG00000186197|EDARADD	86.68380154	1.029663799	0.004863066	0.040974969
ENSG00000130052|STARD8	441.2674585	1.02008359	6.27E-07	2.55E-05
ENSG00000134574|DDB2	2599.229208	1.019267011	3.09E-24	1.13E-21
ENSG00000114646|CSPG5	276.3948363	1.018305199	3.65E-06	0.000116057
ENSG00000131196|NFATC1	374.5782001	1.016776827	2.68E-07	1.19E-05
ENSG00000173727|CMB9-22P13.1	236.0904313	1.015985443	0.00064116	0.008678748
ENSG00000233695|GAS6-AS1	90.64307002	1.015946566	0.005112092	0.042378465
ENSG00000143382|ADAMTSL4	3015.114928	1.015744584	5.70E-13	7.33E-11
ENSG00000184486|POU3F2	601.9934822	1.013003451	8.42E-10	6.26E-08
ENSG00000161955|TNFSF13	109.3966688	1.011012125	0.004858342	0.040954913
ENSG00000110031|LPXN	227.2085501	1.001748377	0.000589306	0.008101272
ENSG00000166341|DCHS1	6138.369791	1.001318268	4.53E-18	9.77E-16
ENSG00000111052|LIN7A	139.625775	1.00045974	0.001191939	0.013924189

Table S2. Downregulated genes in INT-SFT-H cells compared to INT-SFT-L cells from RNA-seq. Column description: gene_id: Ensembl gene ID number; **baseMean:** The average of the normalized count values for the gene across all samples in both conditions; **log2FoldChange:** The estimated effect size between the two groups being compared; **pvalue:** The unadjusted p-value associated with the Wald statistic; **padj:** The p-value adjusted for multiple testing using the Benjamini-Hochberg (BH) procedure.

gene_id	baseMean	log2Fold-Change	pvalue	padj
ENSG00000183508|FAM46C	225.7104286	-1.002512868	9.06E-05	0.001768802
ENSG00000177465|ACOT4	221.6371829	-1.002703959	0.000117474	0.002188542

ENSG00000116701|NCF2	102.8668009	-1.002821019	0.003803423	0.034084525
ENSG00000135549|PKIB	234.6856282	-1.003423494	2.05E-05	0.0005064
ENSG00000214517|PPME1	16104.83602	-1.004778061	1.44E-36	1.26E-33
ENSG00000261490|RP11-448G15.3	151.7444341	-1.006129233	0.001842074	0.0196282
ENSG00000205002|AARD	160.2397837	-1.006980691	0.000958108	0.011836579
ENSG00000154655|L3MBTL4	149.377746	-1.012105104	0.000723518	0.009484982
ENSG00000225108|ZBTB45P1	138.0962921	-1.012493592	0.004930713	0.041246626
ENSG00000170458|CD14	171.2070506	-1.017126352	0.000246999	0.003938231
ENSG00000136732|GYPC	987.7393498	-1.026516071	2.90E-08	1.62E-06
ENSG00000077616|NAALAD2	120.1725801	-1.027822819	0.002203746	0.022507575
ENSG00000196787|HIST1H2AG	414.9765846	-1.037602512	3.90E-08	2.10E-06
ENSG00000079263|SP140	146.2034604	-1.040855823	0.001108321	0.013154943
ENSG00000139645|ANKRD52	9290.387647	-1.040933765	1.10E-26	4.80E-24
ENSG00000197142|ACSL5	438.242136	-1.0422818	3.80E-07	1.62E-05
ENSG00000183570|PCBP3	1351.738983	-1.042323027	8.00E-18	1.64E-15
ENSG00000116761|CTH	466.1805629	-1.042729061	3.80E-06	0.000120094
ENSG00000138642|HERC6	530.6304598	-1.042788066	6.17E-09	3.97E-07
ENSG00000261556|SMG1P7	191.2558386	-1.049670336	0.000142261	0.002550016
ENSG00000070669|ASNS	7358.937857	-1.051364138	3.25E-24	1.16E-21
ENSG00000151468|CCDC3	135.1174412	-1.054011545	0.005914451	0.047519556
ENSG00000228544|CCDC183-AS1	112.4857944	-1.055068908	0.004257093	0.0370056
ENSG00000150540|HNMT	971.4725514	-1.056403048	5.74E-14	8.23E-12
ENSG00000058404|CAMK2B	229.4642562	-1.060319148	4.32E-05	0.000936876
ENSG00000163827|LRRC2	246.7115156	-1.063591075	2.06E-05	0.00050706
ENSG00000225614|ZNF469	749.7461437	-1.071701689	1.36E-10	1.19E-08
ENSG00000140465|CYP1A1	99.69776323	-1.072390106	0.003929005	0.034804978
ENSG00000184979|USP18	316.290086	-1.073672397	3.83E-06	0.000120831
ENSG00000283013|RP11-511P7.5	126.5263063	-1.07388579	0.001530202	0.017032027
ENSG00000180596|HIST1H2BC	109.9920805	-1.074955045	0.003088652	0.029002793
ENSG00000062282|DGAT2	118.5304562	-1.076492665	0.000516994	0.007333173
ENSG00000129951|PLPPR3	266.1735027	-1.076631804	1.43E-05	0.000370654
ENSG00000139973|SYT16	302.8466974	-1.079236475	6.38E-05	0.001316989
ENSG00000072506|HSD17B10	3824.592139	-1.079794283	6.08E-20	1.61E-17
ENSG00000174574|AKIRIN1	4258.844511	-1.080087777	6.23E-22	1.98E-19
ENSG00000256229|ZNF486	143.3771172	-1.081436336	0.001011622	0.012345037
ENSG00000197128|ZNF772	252.0050852	-1.081909352	1.14E-06	4.22E-05
ENSG00000185904|LINC00839	355.5179318	-1.082279003	1.01E-08	6.21E-07
ENSG00000240024|LINC00888	113.5992836	-1.099016015	0.000809109	0.010373567
ENSG00000133110|POSTN	3955.560522	-1.101271548	1.95E-15	3.34E-13
ENSG00000206432|TMEM200C	218.5447295	-1.104836958	3.16E-05	0.000720035
ENSG00000132530|XAF1	840.6313837	-1.106495099	5.63E-10	4.35E-08
ENSG00000157601|MX1	3043.584541	-1.107265056	1.58E-29	8.13E-27
ENSG00000168497|SDPR	176.7734599	-1.107743554	8.91E-05	0.001746963
ENSG00000204397|CARD16	359.6936167	-1.112651143	1.75E-08	1.02E-06
ENSG00000196517|SLC6A9	3677.05175	-1.115499131	2.86E-25	1.19E-22
ENSG00000240204|SMKR1	78.52181457	-1.117059202	0.005001777	0.041701358
ENSG00000144331|ZNF385B	114.1741271	-1.122334165	0.000868935	0.010987435
ENSG00000184232|OAF	568.4818016	-1.12370167	1.46E-09	1.04E-07
ENSG00000075461|CACNG4	379.2390606	-1.129813474	3.09E-09	2.06E-07

ENSG00000144959|NCEH1	3283.911063	-1.138172453	3.62E-26	1.54E-23
ENSG00000185621|LMLN	669.5042134	-1.140012455	6.57E-12	7.45E-10
ENSG00000144230|GPR17	105.2364556	-1.140556475	0.002438958	0.024243738
ENSG00000164176|EDIL3	5833.409427	-1.144200177	4.78E-25	1.94E-22
ENSG00000110446|SLC15A3	602.2954593	-1.146037372	2.00E-07	9.18E-06
ENSG00000221955|SLC12A8	1016.867218	-1.147067959	1.47E-15	2.54E-13
ENSG00000154930|ACSS1	430.8778894	-1.150192957	2.50E-10	2.06E-08
ENSG00000183378|OVCH2	88.22238801	-1.156844784	0.001633638	0.017920796
ENSG00000133134|BEX2	127.986281	-1.160511948	0.000201322	0.003334695
ENSG00000135069|PSAT1	19665.3286	-1.163677482	3.98E-31	2.32E-28
ENSG00000144476|ACKR3	593.8447256	-1.187164286	1.23E-09	8.87E-08
ENSG00000132170|PPARG	96.44274844	-1.189136854	0.002267023	0.02292685
ENSG00000248871|TNFSF12-TNFSF13	202.6903733	-1.190670684	0.003981287	0.035137873
ENSG00000144712|CAND2	269.2663161	-1.199884599	3.10E-07	1.36E-05
ENSG00000103021|CCDC113	122.4791911	-1.213769116	0.000125046	0.00230018
ENSG00000175471|MCTP1	210.7611601	-1.226809945	1.02E-06	3.81E-05
ENSG00000117525|F3	2771.263133	-1.229256274	4.87E-27	2.18E-24
ENSG00000233117|LINC00702	112.4910004	-1.2308878	0.000480227	0.006933774
ENSG00000140545|MFGE8	4770.610259	-1.231740327	8.93E-21	2.52E-18
ENSG00000171195|MUC7	117.5478049	-1.241303987	0.000156567	0.002731267
ENSG00000147642|SYBU	383.0029736	-1.246363007	2.88E-09	1.94E-07
ENSG00000163751|CPA3	1400.445777	-1.247480652	6.93E-18	1.44E-15
ENSG00000235180|LINC00601	100.975223	-1.261329006	0.000623321	0.008497124
ENSG00000204291|COL15A1	136.4702717	-1.271849326	3.35E-05	0.000758409
ENSG00000144115|THNSL2	221.8291449	-1.27256505	9.60E-06	0.000268438
ENSG00000160801|PTH1R	125.6864649	-1.278236614	0.00049102	0.007044808
ENSG00000158258|CLSTN2	651.4345226	-1.288763285	6.27E-17	1.22E-14
ENSG00000230606|AC159540.1	88.20207309	-1.317788918	0.000803861	0.010321429
ENSG00000277701|RP11-734K23.9	53.87088757	-1.329805984	0.003767691	0.033833709
ENSG00000025423|HSD17B6	115.7872626	-1.331418187	0.00010856	0.002051496
ENSG00000174564|IL20RB	65.84014087	-1.332085441	0.00200405	0.020920419
ENSG00000050327|ARHGEF5	96.94955214	-1.332196175	0.000254981	0.004050718
ENSG00000108370|RGS9	151.7044275	-1.339898112	1.05E-05	0.000287834
ENSG00000111331|OAS3	5847.005554	-1.35472674	5.70E-38	6.23E-35
ENSG00000100767|PAPLN	675.8053121	-1.35535308	1.11E-18	2.55E-16
ENSG00000116157|GPX7	125.2679845	-1.365535119	9.55E-05	0.001846892
ENSG00000254300|LINC01111	75.2724027	-1.372680581	0.002050717	0.021299878
ENSG00000189423|USP32P3	76.11582394	-1.377145743	0.003755093	0.033755269
ENSG00000204228|HSD17B8	60.50682931	-1.381706505	0.002701262	0.026210186
ENSG00000104313|EYA1	59.22282022	-1.410902418	0.00357484	0.032570556
ENSG00000158406|HIST1H4H	87.07563338	-1.414190656	0.001039568	0.012571939
ENSG00000135605|TEC	59.06953679	-1.430350289	0.002102816	0.021708572
ENSG00000150637|CD226	44.58572519	-1.433397935	0.005118561	0.042391871
ENSG00000145949|MYLK4	71.41773984	-1.433649392	0.002238475	0.022750746
ENSG00000172575|RASGRP1	137.0587118	-1.440906699	0.00030157	0.004676073
ENSG00000054793|ATP9A	438.4483231	-1.449957609	5.53E-15	9.20E-13
ENSG00000183760|ACP7	71.46400665	-1.454741024	0.002021278	0.02106251
ENSG00000187134|AKR1C1	55.3365715	-1.455746653	0.002562457	0.025128468

ENSG00000155846|PPARGC1B	94.68989888	-1.458692195	0.000171826	0.002938023
ENSG00000274021|RP11-823E8.3	42.45944609	-1.478763411	0.003702805	0.033436174
ENSG00000130707|ASS1	2315.674033	-1.481735701	9.30E-36	7.39E-33
ENSG00000168952|STXBP6	226.2068018	-1.482163712	2.58E-09	1.75E-07
ENSG00000112319|EYA4	358.4696529	-1.484812463	2.03E-10	1.69E-08
ENSG00000212123|PRR22	68.769418	-1.49074485	0.003342449	0.030871717
ENSG00000170166|HOXD4	48.47081131	-1.501310969	0.003338854	0.030871147
ENSG00000175274|TP53I11	181.8441066	-1.506168867	3.08E-07	1.35E-05
ENSG00000136404|TM6SF1	42.67559208	-1.507675974	0.005350391	0.043957726
ENSG00000271270|TMCC1-AS1	58.86813692	-1.516233071	0.002577384	0.025218248
ENSG00000128422|KRT17	1540.292416	-1.537105973	1.93E-14	3.04E-12
ENSG00000186472|PCLO	1028.346139	-1.543225957	3.75E-27	1.72E-24
ENSG00000111335|OAS2	1750.151063	-1.545588925	5.86E-36	4.87E-33
ENSG00000107562|CXCL12	908.3377433	-1.548838942	1.03E-24	3.98E-22
ENSG00000280537|RP11-33O4.2	70.62261346	-1.55625618	0.002440328	0.024243738
ENSG00000274917|RNA5-8S5	163.5457297	-1.575380148	0.000330853	0.00506275
ENSG00000158856|DMTN	363.7137883	-1.593116893	7.81E-14	1.11E-11
ENSG00000183979|NPB	71.72603032	-1.597088148	0.002710271	0.026257849
ENSG00000128284|APOL3	345.9847188	-1.608766071	2.42E-16	4.45E-14
ENSG00000128965|CHAC1	1025.831367	-1.613513478	4.71E-21	1.37E-18
ENSG00000151012|SLC7A11	8403.369705	-1.661464882	1.07E-36	9.81E-34
ENSG00000249306|LINC01411	47.98490938	-1.663844702	0.002488946	0.024600861
ENSG00000276700|RNA5-8S5	147.264559	-1.669335382	0.000227723	0.003691526
ENSG00000111846|GCNT2	176.1744361	-1.676196644	6.09E-08	3.17E-06
ENSG00000174951|FUT1	48.31334803	-1.684222625	0.004485509	0.038555958
ENSG00000282851|BISPR	49.7536153	-1.697429683	0.003665025	0.033184621
ENSG00000273730|RNA5-8S5	150.8235669	-1.699805303	0.000188042	0.003159652
ENSG00000137959|IFI44L	591.3095028	-1.72677115	2.18E-20	5.96E-18
ENSG00000275757|RNA5-8S5	144.047993	-1.729995833	0.00024061	0.003846903
ENSG00000138944|KIAA1644	233.9163885	-1.768138792	4.62E-14	6.79E-12
ENSG00000182986|ZNF320	131.3239949	-1.769363345	1.67E-08	9.81E-07
ENSG00000176046|NUPR1	1826.306184	-1.773281867	1.34E-43	2.60E-40
ENSG00000172159|FRMD3	90.80755009	-1.776954438	1.56E-05	0.000399612
ENSG00000188171|ZNF626	79.94490691	-1.787207935	2.28E-05	0.0005508
ENSG00000142185|TRPM2	116.7791913	-1.821697999	4.26E-06	0.000132936
ENSG00000147862|NFIB	985.8676606	-1.832478778	5.52E-42	7.41E-39
ENSG00000233098|CCDC144NL-AS1	57.03160827	-1.849301088	0.000280561	0.004396783
ENSG00000248019|FAM13A-AS1	29.58250736	-1.851226878	0.006223198	0.049414889
ENSG00000170577|SIX2	40.68973997	-1.863709837	0.00331118	0.03064771
ENSG00000262712|RP11-295D4.1	43.1918462	-1.867909494	0.001271561	0.014657339
ENSG00000111305|GSG1	33.13660677	-1.870854603	0.002188177	0.022400933
ENSG00000186487|MYT1L	35.21800647	-1.936641111	0.005069256	0.042149296
ENSG00000116771|AGMAT	85.82818426	-1.943416661	1.71E-06	5.96E-05
ENSG00000163219|ARHGAP25	47.62080089	-1.947457896	0.00021581	0.003541102
ENSG00000132359|RAP1GAP2	632.9787845	-1.956044922	9.52E-35	6.93E-32
ENSG00000134326|CMPK2	62.41037586	-1.98972193	4.19E-06	0.000130833
ENSG00000058866|DGKG	53.32528718	-1.992888293	2.39E-05	0.000575676
ENSG00000171658|RP11-443P15.2	117.0439697	-1.993974113	4.49E-08	2.40E-06

ENSG00000026559|KCNG1	42.3902757	-1.998547933	0.000159884	0.00277366
ENSG00000276903|HIST1H2AL	22.80514029	-2.039362207	0.004039021	0.035557628
ENSG00000105499|PLA2G4C	28.32132357	-2.049184563	0.003899689	0.034698098
ENSG00000131386|GALNT15	63.51539151	-2.061144153	2.08E-06	7.14E-05
ENSG00000089127|OAS1	753.5995037	-2.07634744	3.46E-43	5.49E-40
ENSG00000267520|RP11-373L24.1	74.02017318	-2.080756585	1.78E-05	0.000447569
ENSG00000240288|GHRLOS	57.43968822	-2.122424118	4.34E-06	0.00013496
ENSG00000203877|RIPPLY2	21.54850182	-2.148097011	0.004793874	0.040522527
ENSG00000119514|GALNT12	79.21385328	-2.162607165	7.91E-07	3.11E-05
ENSG00000134256|CD101	41.91102221	-2.179381981	0.000127706	0.00234418
ENSG00000139304|PTPRQ	56.99386815	-2.180387758	4.15E-06	0.000130098
ENSG00000106823|ECM2	60.7558489	-2.197168339	8.75E-07	3.38E-05
ENSG00000211448|DIO2	786.4287991	-2.20506038	2.84E-42	4.13E-39
ENSG00000277957|SENP3-EIF4A1	733.6273122	-2.305211622	3.48E-08	1.90E-06
ENSG00000163017|ACTG2	2278.625528	-2.323317462	3.01E-80	2.63E-76
ENSG00000151967|SCHIP1	274.4946615	-2.357810104	5.43E-16	9.78E-14
ENSG00000148735|PLEKHS1	18.58780433	-2.385435289	0.003211237	0.029976696
ENSG00000196139|AKR1C3	361.9136106	-2.404853784	8.55E-24	2.99E-21
ENSG00000225492|GBP1P1	47.56017027	-2.413572752	3.70E-06	0.000117247
ENSG00000055955|ITIH4	18.04985146	-2.428058052	0.003914723	0.034739763
ENSG00000138798|EGF	68.19833243	-2.45620382	2.81E-08	1.58E-06
ENSG00000179855|GIPC3	215.3863082	-2.472652167	6.63E-19	1.61E-16
ENSG00000267372|AC005330.2	25.09869567	-2.494306538	0.000341188	0.005180064
ENSG00000188517|COL25A1	25.94742245	-2.507502937	0.003556658	0.032421805
ENSG00000126010|GRPR	24.01649024	-2.519865509	0.004679133	0.039673873
ENSG00000233922|AL133493.2	35.17200833	-2.54191322	0.001478842	0.016491867
ENSG00000270617|URGCP-MRPS24	28.74966977	-2.578089923	0.000830099	0.010588311
ENSG00000185860|CCDC190	82.00287003	-2.607367519	2.00E-08	1.15E-06
ENSG00000254510|RP11-867G23.10	43.79198131	-2.609570615	1.78E-06	6.18E-05
ENSG00000011638|TMEM159	237.9737856	-2.617752705	1.05E-24	3.99E-22
ENSG00000065325|GLP2R	41.02397264	-2.623779851	1.20E-05	0.000321935
ENSG00000139269|INHBE	73.3922444	-2.843013798	4.18E-09	2.73E-07
ENSG00000047936|ROS1	54.56808536	-2.882426619	1.41E-05	0.000366271
ENSG00000227811|FAM212B-AS1	23.3233463	-2.985421268	0.004190137	0.036573516
ENSG00000235385|GS1-600G8.5	700.013851	-3.016126222	5.49E-65	2.40E-61
ENSG00000178342|KCNG2	22.00430345	-3.108182792	0.000509888	0.007250137
ENSG00000267886|CTD-2291D10.4	28.32336984	-3.120321122	0.000395472	0.005876597
ENSG00000187908|DMBT1	45.40180428	-3.136563603	2.15E-05	0.000525565
ENSG00000116183|PAPPA2	405.3673152	-3.230421056	3.04E-56	8.87E-53
ENSG00000279184|RP3-323A16.1	21.58244559	-3.29618348	0.000159568	0.002773274
ENSG00000259040|BLOC1S5-TXNDC5	1123.772462	-3.339413416	0.000473598	0.006845423
ENSG00000253679|KB-1410C5.2	20.10815536	-3.420719542	0.000859858	0.010920076
ENSG00000265118|CTD-2370N5.3	12.80657636	-3.42575257	0.004194182	0.036573516
ENSG00000130876|SLC7A10	13.1297718	-3.438502901	0.004165065	0.036392252
ENSG00000280184|AL023806.1	12.29134675	-3.627768781	0.00334125	0.030871717
ENSG00000206532|RP11-553A10.1	91.02463309	-4.32686297	7.97E-05	0.00159394

ENSG00000258989 | RP11-47I22.4	14.5491465	-4.717740141	0.000119578	0.002218283
ENSG00000120337 | TNFSF18	346.497465	-4.782177748	2.57E-11	2.57E-09
ENSG00000154914 | USP43	61.15476042	-5.789097239	7.49E-05	0.001516271
ENSG00000157368 | IL34	12.86038426	-7.066177794	0.00103198	0.012506133

Table S3. PANTHER signaling pathway enrichment analysis using upregulated genes in INT-SFT-H cells from RNA-seq. Significantly enriched pathways ($p < 0.05$) derived from candidate upregulated genes in INT-SFT cells. **Column description: PANTHER Pathways:** names and PANTHER accession numbers for identified signaling pathways; **Related Genes:** the specific differentially expressed genes for the candidate signaling pathway.

Related Genes
DCHS1, PCDHGA10, WNT2, FZD8, SFRP1, PCDHGC5, TLE2, WNT9A, NFATC1, SFRP2, PCDHB5, CDH15, CDH18, AC037459, PCDHB10
DCHS1, PCDHGA10, WNT2, FZD8, PCDHGC5, WNT9A, PCDHB5, CDH15, CDH18, PCDHB10
EPHA3, WNT2, FZD8, SFRP1, EPHB3, LPXN, PDGFA
WNT2, BACE2, FZD8, MMP1, WNT9A
CDKN1A, DDB2, IGFBP3, TNFRSF10D
RPS6KA2, STARD8, PDGFA
CHRNE , ACHE, BCHE
CDKN1A, RPS6KA2
BACE2, APBA2
LAMC1, COL11A1
PBX1, NFATC1
ACHE, BCHE
ACHE, BCHE
NFATC1, AC037459
AC004754, RGS19
AC004754, RGS19
NFATC1, AC037459
GDF15, GDF5
CDKN1A
LPXN
RPS6KA2
MMP1
SNCAIP
CCND2
HES2
GRIA1
GRIA1
DPYSL4
CCND2
EPB41L3
RPS6KA2
TNFRSF10D
NFATC1

Table S4. Overexpressed proteins in INT-SFT-H cells compared to INT-SFT-L cells from mass spectrometry. Column description: Gene Symbol: gene names for the protein; **Accession:** The unique identifier for the protein in UniProt; **log2FoldChange:** The log2-transformed ratio of protein abundance between INT-SFT-H and INT-SFT-L cells; **Description:** The full text describing the protein, typically including its name and organism of origin; **MW in kDa:** Molecular Weight of the protein; # **Unique Peptides:** The number of detected peptides that map exclusively to one specific protein or protein group; # **PSMs (Peptide-Spectrum Matches):** The total number of times a peptide was matched to a mass spectrum, including redundant matches; # **Peptides:** The total number of distinct peptide sequences identified for the protein group; **% Coverage:** The percentage of the total amino acid sequence of the protein that was identified by the detected peptides.

Gene Symbol	Accession	log2FoldChange	Description	MW in kDa	# Unique Peptides	# PSMs	# Peptides	% Coverage	Normalized abundance INT-SFT-H replicate 1	Normalized abundance INT-SFT-H replicate 2	Normalized abundance INT-SFT-H replicate 3	Normalized abundance INT-SFT-L replicate 1	Normalized abundance INT-SFT-L replicate 2	Normalized abundance INT-SFT-L replicate 3
TMOD2	Q9NZR1	2.508648554	Tropomodulin-2 OS=Homo sapiens OX=9606 GN=TMOD2 PE=1 SV=1	39.6	5	19	6	14	22.45010376	24.75429916	21.7055645	20.80341148	20.84995461	19.73065567
HBA1;HBA2	P69905	2.201795578	Hemoglobin subunit alpha OS=Homo sapiens OX=9606 GN=HBA1 PE=1 SV=2	15.2	4	27	4	37	21.43745995	22.91342163	20.89504242	19.94592476	19.58416939	19.11044312
ZNF524	Q96C55	2.139355659	Zinc finger protein 524 OS=Homo sapiens OX=9606 GN=ZNF524 PE=1 SV=1	28.7	1	1	1	4	15.90124416	15.80120277	14.29490757	13.17211533	13.00842857	13.39874363
CDKN2A	Q8N726	1.943820318	Tumor suppressor ARF OS=Homo sapiens OX=9606	13.9	2	4	2	20	16.47357941	16.45603371	16.62399483	14.69370461	14.51422119	14.51422119

			GN=CDKN2A PE=1 SV=2											
WBP2	Q969T9	1.732975006	WW domain-binding protein 2 OS=Homo sapiens OX=9606 GN=WBP2 PE=1 SV=1	28.1	4	11	4	15	18.1801033	17.89652252	18.27866745	16.28951454	16.29670143	16.57015228
ZDHHC5	Q9C0B5	1.578994751	Palmitoyltransferase ZDHHC5 OS=Homo sapiens OX=9606 GN=ZDHHC5 PE=1 SV=2	77.5	14	28	14	26	19.31007957	19.01835251	19.30117416	17.59514046	17.6382103	17.65927124
BNIP3	Q12983	1.250944455	BCL2/adenovirus E1B 19 kDa protein-interacting protein 3 OS=Homo sapiens OX=9606 GN=BNIP3 PE=1 SV=4	21.5	1	2	1	6	16.47357941	16.24190903	16.26040459	14.93847656	15.07848454	15.20609856
UAP1L1	Q3KQV9	1.064320246	UDP-N-acetylhexosamine pyrophosphorylase-like protein 1 OS=Homo sapiens OX=9606 GN=UAP1L1 PE=1 SV=2	57	8	21	10	20	20.02917862	19.67831039	20.00195885	18.73727417	18.83614922	18.94306374
PARP16	Q8N5Y8	1.057538986	Protein mono-ADP-ribosyltransferase PARP16 OS=Homo sapiens OX=9606 GN=PARP16 PE=1 SV=2	36.4	1	2	1	3	13.99859142	13.46403503	13.10656261	12.3049221	12.19229317	12.89935684

LAMC1	P11047	1.030221939	Laminin subunit gamma-1 OS=Homo sapiens OX=9606 GN=LAMC1 PE=1 SV=3	177.5	29	67	29	21	22.23022652	21.86514091	22.27355385	21.11426163	21.0625	21.10149384
RGS19	P49795	1.023645401	Regulator of G-protein signaling 19 OS=Homo sapiens OX=9606 GN=RGS19 PE=1 SV=1	24.6	4	10	4	18	18.02106667	18.08472443	18.05854225	16.98815155	17.09506798	17.01017761
PUDP	Q08623	0.988205592	Pseudouridine-5'-phosphatase OS=Homo sapiens OX=9606 GN=PUDP PE=1 SV=3	25.2	2	9	2	10	17.94877815	17.94877815	18.00470352	16.72067261	16.96578407	17.25118637
PPIL1	Q9Y3C6	0.948856354	Peptidyl-prolyl cis-trans isomerase-like 1 OS=Homo sapiens OX=9606 GN=PPIL1 PE=1 SV=1	18.2	7	21	7	57	21.04260063	21.60512543	20.96013832	20.18252945	20.39623642	20.18252945
AMZ2	Q86W34	0.919056892	Archaemetzincin-2 OS=Homo sapiens OX=9606 GN=AMZ2 PE=1 SV=2	41.2	2	2	2	5	14.01017857	13.88226128	13.95673943	13.23332024	13.1969099	12.66177845
CCNY	Q8ND76	0.895954768	Cyclin-Y OS=Homo sapiens OX=9606 GN=CCNY PE=1 SV=2	39.3	4	12	7	30	18.9629631	18.7634449	19.0237751	17.98259163	18.02647972	18.05324745
CPPED1	Q9BRF8	0.895423571	Serine/threonine-protein phosphatase CPPED1 OS=Homo sapiens OX=9606	35.5	2	2	2	6	16.87267494	16.68002892	17.02106667	15.89415455	15.96578407	16.02756119

			GN=CPPED1 PE=1 SV=3											
BLOC1S5	Q8TDH9	0.856221835	Biogenesis of lysosome-related organelles complex 1 subunit 5 OS=Homo sapiens OX=9606 GN=BLOC1S5 PE=1 SV=1	21.6	3	8	3	17	16.16545677	16.10328865	16.17718506	15.47674656	15.18301487	15.21750355
SPHK1	Q9NYA1	0.850465139	Sphingosine kinase 1 OS=Homo sapiens OX=9606 GN=SPHK1 PE=1 SV=1	42.5	1	3	1	2	15.29490757	14.91064262	15.31274128	14.39204884	14.2585659	14.31628132
CLDND1	Q9NY35	0.831794739	Claudin domain-containing protein 1 OS=Homo sapiens OX=9606 GN=CLDND1 PE=1 SV=1	28.6	1	4	1	6	17.46562958	17.0318737	17.47357941	16.47516251	16.49404335	16.50649261
CA9	Q16790	0.828760783	Carbonic anhydrase 9 OS=Homo sapiens OX=9606 GN=CA9 PE=1 SV=2	49.7	2	3	2	9	15.45121098	15.47990608	15.47990608	14.87267494	14.62685013	14.42521572
CDKN1A	P38936	0.814717293	Cyclin-dependent kinase inhibitor 1 OS=Homo sapiens OX=9606 GN=CDKN1A PE=1 SV=3	18.1	2	4	2	18	13.50183678	13.9476366	13.83368111	13.01889515	12.82575417	12.99435329

TSPYL1	Q9H0U9	0.798419317	Testis-specific Y-encoded-like protein 1 OS=Homo sapiens OX=9606 GN=TSPYL1 PE=1 SV=3	49.2	6	15	6	17	19.1180687	18.70049477	19.11044312	18.2041893	18.12060165	18.20895767
TNC	P24821	0.79367129	Tenascin OS=Homo sapiens OX=9606 GN=TNC PE=1 SV=3	240.7	14	22	14	8	19.24655533	19.44960022	19.21845055	18.48148346	18.51267815	18.53943062
CA2	P00918	0.783232371	Carbonic anhydrase 2 OS=Homo sapiens OX=9606 GN=CA2 PE=1 SV=2	29.2	5	12	5	25	19.2877121	18.91415215	19.33429146	18.48934555	18.29670143	18.40041161
PSTPIP2	Q9H939	0.776852926	Proline-serine-threonine phosphatase-interacting protein 2 OS=Homo sapiens OX=9606 GN=PSTPIP2 PE=1 SV=4	38.8	9	21	9	31	19.18495369	19.0237751	19.22081375	18.39623642	18.32333565	18.3794117
PARD3	Q8TEW0	0.771922429	Partitioning defective 3 homolog OS=Homo sapiens OX=9606 GN=PARD3 PE=1 SV=2	151.3	33	75	33	30	21.50203133	21.2877121	21.5308857	20.65403366	20.64526367	20.7055645
SERPINB7	O75635	0.765871048	Serpin B7 OS=Homo sapiens OX=9606 GN=SERPINB7 PE=1 SV=1	42.9	3	4	3	7	17.02106667	17.14569283	17.13570976	16.40872765	16.416996	16.17913246
SLC2A14	Q8TDB8	0.761076609	Solute carrier family 2, facilitated glucose	56.3	1	2	1	2	15.72067165	15.36837006	15.82127571	14.85330963	14.76797771	15.00580025

			transporter member 14 OS=Homo sapiens OX=9606 GN=SLC2A14 PE=1 SV=1										
KIAA0319L	Q8IZA0	0.742192586	Dyslexia-associated protein KIAA0319-like protein OS=Homo sapiens OX=9606 GN=KIAA0319L PE=1 SV=2	115.6	3	6	3	4	17.24190903	16.81127357	17.33210564	16.37517548	16.42028999	16.36324501
DDB2	Q92466	0.739854177	DNA damage-binding protein 2 OS=Homo sapiens OX=9606 GN=DDB2 PE=1 SV=1	47.8	9	20	9	26	20.42726326	20.08212852	20.46762085	19.6132431	19.58783531	19.55637169
NNT	Q13423	0.724656423	NAD(P) transhydrogenase, mitochondrial OS=Homo sapiens OX=9606 GN=NNT PE=1 SV=3	113.8	29	74	29	32	23.57542419	23.26785278	23.57542419	22.74509239	22.71607208	22.78356743
PLEKHA7	Q6IQ23	0.722700119	Pleckstrin homology domain-containing family A member 7 OS=Homo sapiens OX=9606 GN=PLEKHA7 PE=1 SV=2	127.1	4	6	4	4	16.7072506	16.50494385	16.7602005	15.89178371	15.81127453	16.10123634
LDHA	P00338	0.719929377	L-lactate dehydrogenase A chain OS=Homo	36.7	24	238	26	71	26.65948868	26.32044601	26.69975281	25.85281372	25.85043144	25.81665421

			sapiens OX=9606 GN=LDHA PE=1 SV=2											
FKBP5	Q13451	0.703492483	Peptidyl-prolyl cis-trans isomerase FKBP5 OS=Homo sapiens OX=9606 GN=FKBP5 PE=1 SV=2	51.2	9	20	9	25	18.76991463	18.43338966	18.82687187	17.98815155	17.97140884	17.96013832
RNASET2	O00584	0.696008682	Ribonuclease T2 OS=Homo sapiens OX=9606 GN=RNASET2 PE=1 SV=2	29.5	3	4	3	13	17.43338966	17.24190903	17.59514046	16.81127357	16.62399483	16.7471447
ANKRD16	Q6P6B7	0.68870608	Ankyrin repeat domain-containing protein 16 OS=Homo sapiens OX=9606 GN=ANKRD16 PE=1 SV=1	39.3	1	2	1	5	17.12565613	16.8237648	17.04260063	16.16937637	16.2877121	16.46881485
DTNBP1	Q96EV8	0.666334152	Dysbindin OS=Homo sapiens OX=9606 GN=DTNBP1 PE=1 SV=1	39.5	4	8	4	13	18.31007957	18.11553192	18.38784981	17.60240936	17.60240936	17.60964012
FBXO3	Q9UK99	0.641968727	F-box only protein 3 OS=Homo sapiens OX=9606 GN=FBXO3 PE=1 SV=3	54.5	2	6	2	7	15.87987041	16.09300423	16.05959702	15.46403503	15.29131508	15.35121536

NEU1	Q99519	0.637915929	Sialidase-1 OS=Homo sapiens OX=9606 GN=NEU1 PE=1 SV=1	45.4	9	18	9	33	19.91269112	20.43745995	19.93156815	19.49716568	19.44354248	19.42726326
CHCHD6	Q9BRQ6	0.628734589	MICOS complex subunit MIC25 OS=Homo sapiens OX=9606 GN=CHCHD6 PE=1 SV=1	26.4	4	7	4	20	18.7634449	18.19460297	18.83614922	18.16054153	17.94877815	17.79867363
ACAT1	P24752	0.625881831	Acetyl-CoA acetyltransferase, mitochondrial OS=Homo sapiens OX=9606 GN=ACAT1 PE=1 SV=1	45.2	15	55	15	42	22.66492271	22.32937241	22.63422585	21.91706848	21.89504242	21.93876457
SERPINF1	P36955	0.611790975	Pigment epithelium-derived factor OS=Homo sapiens OX=9606 GN=SERPINF1 PE=1 SV=4	46.3	1	5	1	3	13.76279736	14.11146164	13.84352875	13.14529514	13.53867435	13.19844532
PCOLCE	Q15113	0.595064163	Procollagen C-endopeptidase enhancer 1 OS=Homo sapiens OX=9606 GN=PCOLCE PE=1 SV=2	47.9	6	8	6	18	17.416996	16.89652252	17.51267815	16.77313995	16.58783531	16.68002892
PDZD8	Q8NEN9	0.59379069	PDZ domain-containing protein 8 OS=Homo sapiens OX=9606 GN=PDZD8 PE=1 SV=1	128.5	4	6	4	4	14.68825054	14.51422119	14.90124416	14.43175888	13.86302471	14.02756023

KCTD14	Q9BQ13	0.591405869	BTB/POZ domain-containing protein KCTD14 OS=Homo sapiens OX=9606 GN=KCTD14 PE=1 SV=2	29.6	1	2	1	5	17.85452843	17.48934555	17.81753349	17.24190903	16.93156815	17.21371269
PHF10	Q8WUB8	0.591398239	PHD finger protein 10 OS=Homo sapiens OX=9606 GN=PHF10 PE=1 SV=3	56	6	14	6	15	19.72400665	19.15313721	19.72234154	18.90242195	18.94306374	18.97980499
ARFIP2	P53365	0.586859385	Arfaptin-2 OS=Homo sapiens OX=9606 GN=ARFIP2 PE=1 SV=1	37.8	7	21	7	22	19.64350319	19.27866745	19.46762085	18.83922768	18.98815155	18.80183411
MADD	Q8WXG6	0.571407318	MAP kinase-activating death domain protein OS=Homo sapiens OX=9606 GN=MADD PE=1 SV=2	183.2	12	16	12	9	17.77313995	17.7072506	17.93732834	17.25118637	17.15560913	17.29670143
TERF2	Q15554	0.562209447	Telomeric repeat-binding factor 2 OS=Homo sapiens OX=9606 GN=TERF2 PE=1 SV=3	59.6	14	22	14	30	20.37517548	19.92578697	20.38574409	19.688591	19.66622353	19.64526367
TRAF6	Q9Y4K3	0.551762899	TNF receptor-associated factor 6 OS=Homo sapiens OX=9606 GN=TRAF6 PE=1 SV=1	59.5	4	9	4	8	18.50881577	18.65228462	18.45362473	17.90829849	18.16054153	17.89059639
NKAP	Q8N5F7	0.544497172	NF-kappa-B-activating protein OS=Homo	47.1	2	6	2	6	17.04260063	16.81127357	17.01017761	16.37687111	16.53564072	16.31804848

			sapiens OX=9606 GN=NKAP PE=1 SV=1												
FBH1	Q8NFZ0	0.543254217	F-box DNA helicase 1 OS=Homo sapiens OX=9606 GN=FBH1 PE=1 SV=2	117.6	4	8	4	4	18.29221344	18.02647972	18.31451225	17.61683655	17.65927124	17.72733498
CARM1	Q86X55	0.539063136	Histone-arginine methyltransferase CARM1 OS=Homo sapiens OX=9606 GN=CARM1 PE=1 SV=3	65.8	9	23	9	18	20.35380173	20.62756157	20.40665436	19.89799881	19.91269112	19.96013832
CERS1	P27544	0.535453796	Ceramide synthase 1 OS=Homo sapiens OX=9606 GN=CERS1 PE=1 SV=1	39.5	1	2	1	4	17.2041893	17.02106667	17.31451225	16.73396873	16.5612278	16.6382103
HDHD2	Q9H0R4	0.530326207	Haloacid dehalogenase-like hydrolase domain-containing protein 2 OS=Homo sapiens OX=9606 GN=HDHD2 PE=1 SV=1	28.5	8	16	8	42	20.91706848	20.52611732	20.87267494	20.20657539	20.24190903	20.27639771
NXT2	Q9NPJ8	0.529677709	NTF2-related export protein 2 OS=Homo sapiens OX=9606 GN=NXT2 PE=1 SV=1	16.2	3	10	3	32	17.98815155	18.14569283	18.12565613	17.44960022	17.52037239	17.70049477
PTCD2	Q8WV60	0.526710828	Pentatricopeptide repeat-containing protein	43.9	1	2	1	2	14.56271935	14.06170845	14.36495495	13.74188805	13.83368111	13.83368111

			2, mitochondrial OS=Homo sapiens OX=9606 GN=PTCD2 PE=1 SV=3											
TM7SF2	O76062	0.526040713	Delta(14)-sterol reductase TM7SF2 OS=Homo sapiens OX=9606 GN=TM7SF2 PE=1 SV=3	46.4	1	2	1	2	15.69642448	15.75759792	16.10942268	15.15165043	15.43827248	15.39540005
CCDC90B	Q9GZT6	0.525997162	Coiled-coil domain-containing protein 90B, mitochondrial OS=Homo sapiens OX=9606 GN=CCDC90B PE=1 SV=2	29.5	2	4	2	12	15.15955639	15.09093952	15.29490757	14.61539936	14.73661327	14.61539936
NLRP10	Q86W26	0.521130244	NACHT, LRR and PYD domains-containing protein 10 OS=Homo sapiens OX=9606 GN=NLRP10 PE=1 SV=1	75	3	5	3	4	16.56271935	16.06802177	16.51576233	15.87267494	15.89415455	15.81628323
TLL1	O43897	0.518419584	Tolloid-like protein 1 OS=Homo sapiens OX=9606 GN=TLL1 PE=1 SV=1	114.6	1	1	1	1	13.47674656	13.96578407	13.60964012	13.23032093	13.20151138	13.06507969
HDHD5	Q9BXW7	0.510672887	Haloacid dehalogenase-like hydrolase	46.3	6	17	6	17	19.87417603	19.68517303	19.92433739	19.31672287	19.2854557	19.34948921

			domain-containing 5 OS=Homo sapiens OX=9606 GN=HDHD5 PE=1 SV=1											
ZCCHC17	Q9NP64	0.507852554	Zinc finger CCHC domain-containing protein 17 OS=Homo sapiens OX=9606 GN=ZCCHC17 PE=1 SV=1	27.6	5	12	5	26	19.21371269	19.32991982	19.1481781	18.7923336	18.73065567	18.64526367
TRIM22	Q8IYM9	0.507123311	E3 ubiquitin-protein ligase TRIM22 OS=Homo sapiens OX=9606 GN=TRIM22 PE=1 SV=1	56.9	5	12	5	14	18.58416939	18.3794117	18.68346024	18.01563263	18.00470352	18.10533524
RPS6KB1	P23443	0.503767649	Ribosomal protein S6 kinase beta-1 OS=Homo sapiens OX=9606 GN=RPS6KB1 PE=1 SV=2	59.1	6	11	7	13	17.0318737	16.65228462	16.8237648	16.33734322	16.28410149	16.37517548
SELENOH	Q8IZQ5	0.503749212	Selenoprotein H OS=Homo sapiens OX=9606 GN=SELENOH PE=1 SV=2	13.4	5	12	5	49	19.12060165	18.86665154	19.21845055	18.61683655	18.45362473	18.62399483
YRDC	Q86U90	0.499710719	Threonylcarbamoyl-AMP synthase OS=Homo sapiens	29.3	1	1	1	5	17.60964012	17.53564072	17.72067261	17.06381607	17.04260063	17.26040459

			OX=9606 GN=YRDC PE=1 SV=1											
SDF4	Q9BRK5	0.496021271	45 kDa calcium-binding protein OS=Homo sapiens OX=9606 GN=SDF4 PE=1 SV=1	41.8	14	41	14	50	21.33210564	20.86514091	21.24190903	20.7055645	20.65403366	20.59149361
GATD3	P0DPI2	0.495128632	Glutamine amidotransferase-like class 1 domain-containing protein 3, mitochondrial OS=Homo sapiens OX=9606 GN=GATD3 PE=1 SV=1	28.2	8	23	8	32	22.1332016	21.73479652	22.11743546	21.54037857	21.45763779	21.50203133
KLC4	Q9NSK0	0.485904694	Kinesin light chain 4 OS=Homo sapiens OX=9606 GN=KLC4 PE=1 SV=3	68.6	14	66	20	36	20.34299469	20.10789108	20.38574409	19.80498886	19.74550438	19.82842255
COIL	P38432	0.479322433	Coilin OS=Homo sapiens OX=9606 GN=COIL PE=1 SV=1	62.6	12	27	12	27	20.71397781	20.98119926	20.77152824	20.32113457	20.35380173	20.35380173
PFKFB3	Q16875	0.478627523	6-phosphofructo-2-kinase/fructose-2,6-bisphosphatase 3 OS=Homo sapiens OX=9606 GN=PFKFB3 PE=1 SV=1	59.6	7	16	9	18	18.82687187	18.69370461	18.7634449	18.24190903	18.33210564	18.27412415
NSD3	Q9BZ95	0.478049596	Histone-lysine N-methyltransferase NSD3	161.5	10	17	10	11	19.50494385	19.24887276	19.55074692	18.96859932	18.90536308	18.99645233

			OS=Homo sapiens OX=9606 GN=NSD3 PE=1 SV=1											
ITGA1	P56199	0.459337234	Integrin alpha-1 OS=Homo sapiens OX=9606 GN=ITGA1 PE=1 SV=2	130.8	19	39	19	15	20.36452866	20.68002892	20.44758415	20.05589676	20.01563263	20.04260063
DGKA	P23743	0.458689372	Diacylglycerol kinase alpha OS=Homo sapiens OX=9606 GN=DGKA PE=1 SV=3	82.6	15	28	15	27	21.24190903	20.93876457	21.29333687	20.688591	20.65403366	20.75531769
SBF1	O95248	0.457162221	Myotubularin-related protein 5 OS=Homo sapiens OX=9606 GN=SBF1 PE=1 SV=4	208.3	16	38	18	13	19.60964012	19.34516335	19.65752792	19.07169342	19.06381607	19.10533524
P4HA1	P13674	0.456761042	Prolyl 4-hydroxylase subunit alpha-1 OS=Homo sapiens OX=9606 GN=P4HA1 PE=1 SV=2	61	24	99	24	60	24.22434998	23.85756874	24.21697044	23.62086868	23.6320076	23.67572975
NACAD	O15069	0.456671397	NAC-alpha domain-containing protein 1 OS=Homo sapiens OX=9606 GN=NACAD PE=1 SV=3	161	9	15	9	10	18.86363029	18.59514046	18.88166428	18.26499176	18.29670143	18.40872765
PACSIN3	Q9UKS6	0.456005732	Protein kinase C and casein kinase substrate in neurons protein 3	48.5	14	41	14	39	20.55449867	20.24190903	20.54510117	20.02917862	19.92868042	20.01563263

			OS=Homo sapiens OX=9606 GN=PACSIN3 PE=1 SV=2											
BLOC1S3	Q6QNY0	0.452932358	Biogenesis of lysosome-related organelles complex 1 subunit 3 OS=Homo sapiens OX=9606 GN=BLOC1S3 PE=1 SV=1	21.2	3	10	3	21	18.23257065	17.95446968	18.27866745	17.70049477	17.65927124	17.7471447
NDRG1	Q92597	0.450286229	Protein NDRG1 OS=Homo sapiens OX=9606 GN=NDRG1 PE=1 SV=1	42.8	6	32	6	24	20.15807724	19.82531929	20.26499176	19.59331894	19.62934303	19.67486763
UPP1	Q16831	0.449660619	Uridine phosphorylase 1 OS=Homo sapiens OX=9606 GN=UPP1 PE=1 SV=1	33.9	8	16	8	31	19.82531929	19.58416939	19.80656242	19.23257065	19.27639771	19.35810089
COL18A1	P39060	0.44589742	Collagen alpha-1(XVIII) chain OS=Homo sapiens OX=9606 GN=COL18A1 PE=1 SV=5	178.1	20	47	20	18	22.43237114	22.17035484	22.48246956	21.91706848	21.90609741	21.92433739
MLPH	Q9BV36	0.445718447	Melanophilin OS=Homo sapiens OX=9606 GN=MLPH PE=1 SV=1	65.9	3	6	3	6	16.07639885	15.97701168	16.05959702	15.6296978	15.58343506	15.56271935
TCAF2	A6NFQ2	0.444404602	TRPM8 channel-associated factor 2 OS=Homo	100.8	3	5	3	5	16.94306374	16.65228462	17.05324745	16.38194847	16.52802658	16.40540695

			sapiens OX=9606 GN=TCAF2 PE=1 SV=2											
RFLNB	Q8N5W9	0.441150665	Refilin-B OS=Homo sapiens OX=9606 GN=RFLNB PE=1 SV=1	22.9	4	8	4	36	19.05589676	19.03456306	19.10789108	18.63112068	18.59149361	18.65228462
HNRNPUL1	Q9BUJ2	0.441116969	Heterogeneous nuclear ribonucleoprotein U-like protein 1 OS=Homo sapiens OX=9606 GN=HNRNPUL1 PE=1 SV=2	95.7	26	84	26	38	22.416996	22.58233261	22.48000526	22.01903152	21.98815155	22.1487999
P4HA2	O15460	0.437401454	Prolyl 4-hydroxylase subunit alpha-2 OS=Homo sapiens OX=9606 GN=P4HA2 PE=1 SV=1	60.9	24	87	24	53	23.74919128	23.46762085	23.76951218	23.2110405	23.18101311	23.28206635
FAM133B	Q5BKY9	0.436723709	Protein FAM133B OS=Homo sapiens OX=9606 GN=FAM133B PE=1 SV=1	28.4	2	4	2	7	17.50494385	17.48934555	17.53564072	17.23257065	16.97701073	17.01017761
RASA3	Q14644	0.435127894	Ras GTPase-activating protein 3 OS=Homo sapiens OX=9606 GN=RASA3 PE=1 SV=3	95.6	6	12	7	10	17.416996	17.29670143	17.37517548	17.05324745	16.93156815	16.79867363
FMN2	Q9NZ56	0.430251439	Formin-2 OS=Homo sapiens OX=9606 GN=FMN2 PE=1 SV=4	180	23	51	23	21	20.87267494	20.87267494	20.85756874	20.44758415	20.416996	20.44758415

GID4	Q8IVV7	0.428712527	Glucose-induced degradation protein 4 homolog OS=Homo sapiens OX=9606 GN=GID4 PE=1 SV=1	33.5	1	2	1	4	15.45121098	15.71531868	15.43827248	15.22128487	15.07848454	15.01889515
PLOD2	O00469	0.42587026	Procollagen-lysine,2-oxoglutarate 5-dioxygenase 2 OS=Homo sapiens OX=9606 GN=PLOD2 PE=1 SV=2	84.6	28	104	28	43	24.49228287	24.20209694	24.52850342	23.97596359	23.97596359	23.99334526
CUL2	Q13617	0.425561269	Cullin-2 OS=Homo sapiens OX=9606 GN=CUL2 PE=1 SV=2	86.9	22	52	22	30	22.73892403	22.44252968	22.7634449	22.21252441	22.17645645	22.27923393
DNM1	Q05193	0.412877401	Dynamin-1 OS=Homo sapiens OX=9606 GN=DNM1 PE=1 SV=2	97.3	16	68	23	30	20.73892403	20.46762085	20.75531769	20.20657539	20.20657539	20.31007957
CWC15	Q9P013	0.410556793	Spliceosome-associated protein CWC15 homolog OS=Homo sapiens OX=9606 GN=CWC15 PE=1 SV=2	26.6	7	13	7	31	20.21845055	20.21845055	20.21845055	20.02917862	19.74221802	19.65228462
PRUNE1	Q86TP1	0.403980891	Exopolyphosphatase PRUNE1 OS=Homo sapiens OX=9606 GN=PRUNE1 PE=1 SV=2	50.2	6	12	6	15	19.98815155	19.74386215	20.04260063	19.49521446	19.52802658	19.53943062
SBF2	Q86WG5	0.402604421	Myotubularin-related protein 13 OS=Homo	208.3	6	14	9	5	17.71397781	17.54321289	17.84842682	17.33210564	17.25118637	17.31451225

			sapiens OX=9606 GN=SBF2 PE=1 SV=1											
TIGAR	Q9NQ88	0.401427587	Fructose-2,6-bisphosphatase TIGAR OS=Homo sapiens OX=9606 GN=TIGAR PE=1 SV=1	30	6	20	6	31	20.67141724	20.416996	20.66275215	20.17035484	20.21845055	20.15807724
DUS2	Q9NX74	0.400857925	tRNA-dihydrouridine(20) synthase [NAD(P)+]-like OS=Homo sapiens OX=9606 GN=DUS2 PE=1 SV=1	55	9	19	10	23	18.44151688	18.20895767	18.46562958	17.87867355	17.96578407	18.06907272
KDM3A	Q9Y4C1	0.39915212	Lysine-specific demethylase 3A OS=Homo sapiens OX=9606 GN=KDM3A PE=1 SV=4	147.2	12	23	13	10	19.31672287	19.1231308	19.31451225	18.85148048	18.8237648	18.88166428
SLC25A15	Q9Y619	0.394121488	Mitochondrial ornithine transporter 1 OS=Homo sapiens OX=9606 GN=SLC25A15 PE=1 SV=1	32.7	1	1	1	2	15.90124416	15.87026882	15.7910614	15.59222317	15.5111351	15.27685165
TBX3	O15119	0.393760045	T-box transcription factor TBX3 OS=Homo sapiens OX=9606 GN=TBX3 PE=1 SV=4	79.3	3	5	3	5	15.62113571	15.82873154	15.82873154	15.37856579	15.43827248	15.28048038

PLEKHA2	Q9HB19	0.390967051	Pleckstrin homology domain-containing family A member 2 OS=Homo sapiens OX=9606 GN=PLEKHA2 PE=1 SV=3	47.2	8	14	8	21	19.31007957	19.19940376	19.40457535	18.93732834	18.86363029	18.9401989
PDGFRB	P09619	0.384477615	Platelet-derived growth factor receptor beta OS=Homo sapiens OX=9606 GN=PDGFRB PE=1 SV=1	123.9	8	19	9	9	18.40457535	18.25580406	18.54321289	18.06381607	17.95446968	18.0318737
KCTD3	Q9Y597	0.384381612	BTB/POZ domain-containing protein KCTD3 OS=Homo sapiens OX=9606 GN=KCTD3 PE=1 SV=2	88.9	11	32	13	17	19.90536308	19.74386215	19.90242195	19.48345375	19.47555923	19.43948936
REPS2	Q8NFH8	0.383234024	RalBP1-associated Eps domain-containing protein 2 OS=Homo sapiens OX=9606 GN=REPS2 PE=1 SV=2	71.5	5	16	6	12	18.26499176	18.02106667	18.34082413	17.84842682	17.80498886	17.8237648
DDX41	Q9UJV9	0.381628672	Probable ATP-dependent RNA helicase DDX41 OS=Homo sapiens OX=9606 GN=DDX41 PE=1 SV=2	69.8	14	31	14	27	20.72234154	20.52611732	20.83460617	20.29893875	20.25349617	20.38574409

SYNE3	Q6ZMZ3	0.38013649	Nesprin-3 OS=Homo sapiens OX=9606 GN=SYNE3 PE=1 SV=2	112.1	7	12	7	9	16.50649261	16.69370461	16.5612278	16.18881989	16.29849243	16.13370323
PIK3C2A	O00443	0.376586278	Phosphatidylinositol 4-phosphate 3-kinase C2 domain-containing subunit alpha OS=Homo sapiens OX=9606 GN=PIK3C2A PE=1 SV=2	190.6	29	51	29	18	21.24190903	21.01563263	21.28206635	20.80341148	20.82687187	20.77956581
EML4	Q9HC35	0.375829697	Echinoderm microtubule-associated protein-like 4 OS=Homo sapiens OX=9606 GN=EML4 PE=1 SV=3	108.8	24	64	24	32	21.67572975	21.65403366	21.67572975	21.35380173	21.27070618	21.25349617
ITGA3	P26006	0.372997284	Integrin alpha-3 OS=Homo sapiens OX=9606 GN=ITGA3 PE=1 SV=5	116.5	14	48	14	15	22.7055645	22.82493019	22.70977783	22.41956902	22.37782478	22.32388687
GYS1	P13807	0.370573044	Glycogen [starch] synthase, muscle OS=Homo sapiens OX=9606 GN=GYS1 PE=1 SV=2	83.7	20	60	20	37	22.03925705	22.22729111	22.049263	21.71397781	21.73479652	21.75531769
RNF2	Q99496	0.364021937	E3 ubiquitin-protein ligase RING2 OS=Homo sapiens	37.6	2	9	3	11	17.17523766	17.09506798	17.16545677	16.8237648	16.78596306	16.73396873

			OX=9606 GN=RNF2 PE=1 SV=1											
SEPTIN6	Q14141	0.359029134	Septin-6 OS=Homo sapiens OX=9606 GN=SEPTIN6 PE=1 SV=4	49.7	6	55	13	40	20.60964012	20.44758415	20.66275215	20.21845055	20.18252945	20.24190903
UACA	Q9BZF9	0.354661306	Uveal autoantigen with coiled-coil domains and ankyrin repeats OS=Homo sapiens OX=9606 GN=UACA PE=1 SV=2	162.4	46	110	47	33	22.66275215	22.47010612	22.67788124	22.20955276	22.22434998	22.31285286
RUFY2	Q8WXA3	0.344694138	RUN and FYVE domain-containing protein 2 OS=Homo sapiens OX=9606 GN=RUFY2 PE=1 SV=3	70	13	30	15	24	20.32113457	20.2877121	20.416996	19.919981	20.02917862	20.04260063
EPB41	P11171	0.33701388	Protein 4.1 OS=Homo sapiens OX=9606 GN=EPB41 PE=1 SV=4	97	9	19	11	16	18.50106049	18.52420425	18.59149361	18.24655533	18.14071083	18.21845055
PIAS3	Q9Y6X2	0.322154045	E3 SUMO-protein ligase PIAS3 OS=Homo sapiens OX=9606 GN=PIAS3 PE=1 SV=2	68	1	2	1	2	14.54472256	14.46403503	14.52037334	14.22128487	14.1276722	14.21371174
SMTN	P53814	0.3205808	Smoothelin OS=Homo sapiens OX=9606 GN=SMTN PE=1 SV=7	99	23	47	23	24	19.91269112	19.84383583	19.98815155	19.62578011	19.56383705	19.59331894

PRKAR2B	P31323	0.320451101	cAMP-dependent protein kinase type II-beta regulatory subunit OS=Homo sapiens OX=9606 GN=PRKAR2B PE=1 SV=3	46.3	8	18	10	37	17.49716568	17.44151688	17.59514046	17.23257065	17.13570976	17.2041893
RLF	Q13129	0.319515228	Zinc finger protein Rlf OS=Homo sapiens OX=9606 GN=RLF PE=1 SV=2	217.8	6	13	6	4	17.0743084	16.93156815	16.90829849	16.60964012	16.69370461	16.65228462
ADAM15	Q13444	0.316054026	Disintegrin and metalloproteinase domain-containing protein 15 OS=Homo sapiens OX=9606 GN=ADAM15 PE=1 SV=4	92.9	7	12	7	10	17.39204979	17.2877121	17.35810089	16.93156815	17.04260063	17.11553192
INPP4B	O15327	0.309908231	Inositol polyphosphate 4-phosphatase type II OS=Homo sapiens OX=9606 GN=INPP4B PE=1 SV=4	104.7	1	1	1	1	14.773139	14.76797771	14.81378078	14.56271935	14.39204884	14.47040462
ZNF580	Q9UK33	0.309464137	Zinc finger protein 580 OS=Homo sapiens OX=9606 GN=ZNF580 PE=1 SV=1	18.7	1	3	1	10	13.06507969	12.94031334	12.92369843	12.68387222	12.65060329	12.66622353
RAB13	P51153	0.291135788	Ras-related protein Rab-13 OS=Homo	22.8	8	42	11	57	20.97421265	20.88016891	21.01563263	20.688591	20.64526367	20.66275215

			sapiens OX=9606 GN=RAB13 PE=1 SV=1											
GABRA3	P34903	0.282771428	Gamma-aminobutyric acid receptor subunit alpha-3 OS=Homo sapiens OX=9606 GN=GABRA3 PE=1 SV=1	55.1	3	4	3	5	15.84107304	15.70725155	15.78596306	15.52649879	15.45763779	15.50183678
AKAP13	Q12802	0.280081431	A-kinase anchor protein 13 OS=Homo sapiens OX=9606 GN=AKAP13 PE=1 SV=2	307.4	48	91	48	24	21.2877121	21.247715	21.33756065	20.99507141	20.99507141	21.04260063
HELZ	P42694	0.266127268	Probable helicase with zinc finger domain OS=Homo sapiens OX=9606 GN=HELZ PE=1 SV=2	218.8	12	28	12	7	19.38574409	19.45563316	19.44758415	19.2041893	19.1481781	19.1382122
CCAR2	Q8N163	0.26311175	Cell cycle and apoptosis regulator protein 2 OS=Homo sapiens OX=9606 GN=CCAR2 PE=1 SV=2	102.8	41	133	41	56	23.79946518	23.6971035	23.76951218	23.48000526	23.49228287	23.50445747
TRA2A	Q13595	0.255561829	Transformer-2 protein homolog alpha OS=Homo sapiens OX=9606 GN=TRA2A PE=1 SV=1	32.7	4	12	5	16	19.919981	19.90094948	19.90683174	19.65403366	19.69200325	19.61503983

MACROH2A 2	Q9P0M6	0.23517735 8	Core histone macro-H2A.2 OS=Homo sapiens OX=9606 GN=MACROH2A2 PE=1 SV=3	40	1	16	2	7	19.1481781	19.1281757 4	19.1654567 7	18.8994751	18.9373283 4	18.8994751

Table S5. Downregulated proteins in INT-SFT-H cells compared to INT-SFT-L cells from mass spectrometry. Column description: Gene Symbol: gene names for the protein; **Accession:** The unique identifier for the protein in UniProt; **log2FoldChange:** The log2-transformed ratio of protein abundance between INT-SFT-H and INT-SFT-L cells; **Description:** The full text describing the protein, typically including its name and organism of origin; **MW in kDa:** Molecular Weight of the protein; # **Unique Peptides:** The number of detected peptides that map exclusively to one specific protein or protein group; # **PSMs (Peptide-Spectrum Matches):** The total number of times a peptide was matched to a mass spectrum, including redundant matches; # **Peptides:** The total number of distinct peptide sequences identified for the protein group; **% Coverage:** The percentage of the total amino acid sequence of the protein that was identified by the detected peptides.

Gene Symbol	Accession	log2FoldChange	Description	MW in kDa	# Unique Peptides	# PSMs	# Peptides	% Coverage	Normalized abundance INT-SFT-H replicate 1	Normalized abundance INT-SFT-H replicate 2	Normalized abundance INT-SFT-H replicate 3	Normalized abundance INT-SFT-L replicate 1	Normalized abundance INT-SFT-L replicate 2	Normalized abundance INT-SFT-L replicate 3
MX1	P20591	-2.768302282	Interferon-induced GTP-binding protein Mx1 OS=Homo sapiens OX=9606 GN=MX1 PE=1 SV=4	75.5	21	59	25	47	20.34299469	20.02917862	20.42726326	22.98119926	23.04426956	23.07887459
OAS1	P00973	-2.50981013	2'-5'-oligoadenylate synthase 1 OS=Homo sapiens OX=9606 GN=OAS1 PE=1 SV=4	46	8	19	8	23	16.5111351	16.33734322	16.51576233	18.94592476	18.87867355	19.06907272
AKR1C3	P42330	-2.319425583	Aldo-keto reductase family 1 member C3 OS=Homo sapiens OX=9606	36.8	6	9	6	21	16.78596306	16.69370461	16.98815155	19.08731842	19.08990479	19.24887276

			GN=AKR1C3 PE=1 SV=4											
OAS2	P29728	-2.23907725	2'-5'-oligoadenylate synthase 2 OS=Homo sapiens OX=9606 GN=OAS2 PE=1 SV=3	82.4	18	46	19	33	19.75857544	19.62042046	19.840765	21.94592476	21.93517113	22.05589676
MX2	P20592	-1.970006943	Interferon-induced GTP-binding protein Mx2 OS=Homo sapiens OX=9606 GN=MX2 PE=1 SV=1	82	11	33	14	23	18.26040459	18.16054153	18.3709259	20.21845055	20.21845055	20.26499176
F3	P13726	-1.75963974	Tissue factor OS=Homo sapiens OX=9606 GN=F3 PE=1 SV=1	33	5	8	5	20	17.60964012	17.30563354	17.64526367	19.26956558	19.32333565	19.24655533
SLC7A11	Q9UPY5	-1.635138194	Cystine/glutamate transporter OS=Homo sapiens OX=9606 GN=SLC7A11 PE=1 SV=1	55.4	4	8	4	8	20.00195885	19.74221802	19.91122818	21.54980659	21.44252968	21.56848335
OAS3	Q9Y6K5	-1.631181081	2'-5'-oligoadenylate synthase 3 OS=Homo sapiens OX=9606 GN=OAS3 PE=1 SV=3	121.1	24	49	24	27	20.39623642	20.1332016	20.37517548	21.90609741	21.8764267	22.01563263

NCEH1	Q6PIU2	-1.550043106	Neutral cholesterol ester hydrolase 1 OS=Homo sapiens OX=9606 GN=NCEH1 PE=1 SV=3	45.8	12	34	12	38	20.94592476	20.60059547	20.99507141	22.40405655	22.33756065	22.45010376
GYPC	P04921	-1.513722738	Glycophorin-C OS=Homo sapiens OX=9606 GN=GYPC PE=1 SV=1	13.8	1	8	1	20	14.15165043	13.51422119	13.60964012	15.47357845	15.46084023	14.88226128
NFIB	O00712	-1.48458004	Nuclear factor 1 B-type OS=Homo sapiens OX=9606 GN=NFIB PE=1 SV=2	47.4	3	6	4	11	15.70184803	15.75238037	16.88464737	17.60964012	17.59514046	17.58783531
ZC3HAV1L	Q96H79	-1.433403651	Zinc finger CCCH-type antiviral protein 1-like OS=Homo sapiens OX=9606 GN=ZC3HAV1L PE=1 SV=2	32.9	6	13	6	30	17.44960022	17.16545677	17.34082413	18.60240936	18.71061897	18.94306374
MFGE8	Q08431	-1.394299189	Lactadherin OS=Homo sapiens OX=9606 GN=MFGE8 PE=1 SV=3	43.1	3	4	3	11	14.53867435	14.44475651	14.75759792	15.90359974	16.01672173	16.00360489

CASP1	P29466	-1.364109039	Caspase-1 OS=Homo sapiens OX=9606 GN=CASP1 PE=1 SV=1	45.1	5	11	5	13	17.7923336	17.45763779	17.89059639	19.0795269	19.10277557	19.05059242
HSD17B10	Q99714	-1.355364482	3-hydroxyacyl-CoA dehydrogenase type-2 OS=Homo sapiens OX=9606 GN=HSD17B10 PE=1 SV=3	26.9	15	66	15	84	22.2035923	21.88016891	22.21252441	23.40405655	23.42981911	23.52850342
TGM2	P21980	-1.344314257	Protein-glutamine gamma-glutamyl-transferase 2 OS=Homo sapiens OX=9606 GN=TGM2 PE=1 SV=2	77.3	2	4	2	3	13.97477341	12.44604969	13.55074692	14.74714375	14.57459354	14.6827755
STXBP6	Q8NFX7	-1.316109339	Syntaxin-binding protein 6 OS=Homo sapiens OX=9606 GN=STXBP6 PE=1 SV=2	23.5	4	8	4	17	16.48620605	16.51729965	16.60095787	17.78596306	17.8237648	17.94306374
ZNF618	Q5T7W0	-1.296407382	Zinc finger protein 618 OS=Homo sapiens OX=9606 GN=ZNF618 PE=1 SV=1	104.9	3	5	3	4	13.37177658	13.47674656	13.46403503	14.94306374	14.59805202	14.66066456

OASL	Q15646	-1.289165179	2'-5'-oligoadenylate synthase-like protein OS=Homo sapiens OX=9606 GN=OASL PE=1 SV=2	59.2	1	2	1	2	12.82575417	12.00842857	12.04984856	13.88226128	13.66622353	13.20304203
TLE3	Q04726	-1.28635025	Transducin-like enhancer protein 3 OS=Homo sapiens OX=9606 GN=TLE3 PE=1 SV=2	83.4	2	9	6	8	14.00140858	13.16113186	14.40540695	15.22128487	15.3091917	14.89652157
NAPRT	Q6XQN6	-1.285085678	Nicotinate phosphoribosyltransferase OS=Homo sapiens OX=9606 GN=NAPRT PE=1 SV=2	57.5	7	16	7	20	18.53564072	18.31451225	18.62042046	19.7072506	19.75041962	19.86816025
CA3	P07451	-1.279630661	Carbonic anhydrase 3 OS=Homo sapiens OX=9606 GN=CA3 PE=1 SV=3	29.5	4	6	4	19	17.34948921	17.22317123	17.43338966	18.5731144	18.55449867	18.71732903
GBP1	P32455	-1.261537552	Guanylate-binding protein 1 OS=Homo sapiens OX=9606 GN=GBP1 PE=1 SV=2	67.9	15	87	23	45	22.28206635	21.83074379	22.32663155	23.44252968	23.36452866	23.416996

CAND2	O75155	-1.217531522	Cullin-associated NEDD8-dissociated protein 2 OS=Homo sapiens OX=9606 GN=CAND2 PE=1 SV=3	135.2	1	8	2	2	12.45121098	13.55663681	12.9693861	14.06170845	14.27321243	14.29490757
ASS1	P00966	-1.198630651	Argininosuccinate synthase OS=Homo sapiens OX=9606 GN=ASS1 PE=1 SV=2	46.5	10	21	10	23	19.22552681	19.0743084	19.21845055	20.34299469	20.36452866	20.40665436
PARP9	Q8IXQ6	-1.183087031	Protein mono-ADP-ribosyltransferase PARP9 OS=Homo sapiens OX=9606 GN=PARP9 PE=1 SV=2	96.3	6	10	6	8	17.12565613	17.08472443	17.13570976	18.21845055	18.31451225	18.36238861
PTS	Q03393	-1.108137449	6-pyruvoyl tetrahydrobiopterin synthase OS=Homo sapiens OX=9606 GN=PTS PE=1 SV=1	16.4	2	5	2	12	16.07012177	15.77571297	15.8870306	16.93156815	17.11553192	17.01017761
TRIM58	Q8NG06	-1.105321884	E3 ubiquitin-protein ligase TRIM58 OS=Homo sapiens OX=9606 GN=TRIM58 PE=1 SV=2	54.7	15	34	15	31	19.92433739	19.70049477	19.98815155	20.97421265	20.94592476	21.00881195

CAVIN2	O95810	-1.077127775	Caveolae-associated protein 2 OS=Homo sapiens OX=9606 GN=CAVIN2 PE=1 SV=3	47.1	2	3	2	7	15.2877121	14.8087635	15.25488091	16.38700676	15.9992075	16.19652557
RAB6B	Q9NRW1	-1.053325017	Ras-related protein Rab-6B OS=Homo sapiens OX=9606 GN=RAB6B PE=1 SV=1	23.4	2	28	8	49	14.50183678	13.773139	14.42521572	15.42193317	15.2474823	15.19075108
PPME1	Q9Y570	-1.050430298	Protein phosphatase methylesterase 1 OS=Homo sapiens OX=9606 GN=PPME1 PE=1 SV=3	42.3	17	87	17	54	21.51171494	21.35380173	21.65839958	22.50203133	22.49960136	22.67357445
STXBP2	Q15833	-1.039678574	Syntaxin-binding protein 2 OS=Homo sapiens OX=9606 GN=STXBP2 PE=1 SV=2	66.4	6	11	6	13	16.95446968	16.55524826	16.88464737	17.88464737	17.8237648	17.80498886
NEDD4	P46934	-1.039351145	E3 ubiquitin-protein ligase NEDD4 OS=Homo sapiens OX=9606 GN=NEDD4 PE=1 SV=4	149	9	37	12	12	20.04260063	19.82221031	20.15807724	21.049263	21.02917862	21.0625

WHAMM	Q8TF30	-1.039343516	WASP homolog-associated protein with actin, membranes and microtubules OS=Homo sapiens OX=9606 GN=WHAMM PE=1 SV=2	90.9	1	2	1	2	11.50680351	12.15165043	12.02583122	12.99964809	13.07347202	12.72919559
EFEMP1	Q12805	-1.035069466	EGF-containing fibulin-like extracellular matrix protein 1 OS=Homo sapiens OX=9606 GN=EFEMP1 PE=1 SV=2	54.6	2	3	2	6	14.3303566	14.22128487	14.45121098	15.20609856	15.21371174	15.68825054
DTX3L	Q8TDB6	-1.020613352	E3 ubiquitin-protein ligase DTX3L OS=Homo sapiens OX=9606 GN=DTX3L PE=1 SV=1	83.5	12	30	12	19	19.75694656	19.56197357	19.8190937	20.7471447	20.7055645	20.7471447
EDIL3	O43854	-0.9854997	EGF-like repeat and discoidin I-like domain-containing protein 3 OS=Homo sapiens OX=9606 GN=EDIL3 PE=1 SV=1	53.7	3	8	3	8	16.33385468	16.29131508	16.37517548	17.40872765	17.2877121	17.26040459

FHL1	Q13642	-0.980852763	Four and a half LIM domains protein 1 OS=Homo sapiens OX=9606 GN=FHL1 PE=1 SV=4	36.2	9	20	9	29	20.98815155	20.73065567	20.8190937	21.73479652	21.7471447	21.99851799
ITGB3	P05106	-0.957909266	Integrin beta-3 OS=Homo sapiens OX=9606 GN=ITGB3 PE=1 SV=2	87	16	37	16	24	19.34732819	19.15807724	19.44758415	20.25349617	20.23022652	20.34299469
IGF2BP3	O00425	-0.950180054	Insulin-like growth factor 2 mRNA-binding protein 3 OS=Homo sapiens OX=9606 GN=IGF2BP3 PE=1 SV=2	63.7	22	57	23	44	21.02242279	20.75531769	21.01563263	21.86514091	21.87267494	21.90609741
SLC6A9	P48067	-0.949997584	Sodium- and chloride-dependent glycine transporter 1 OS=Homo sapiens OX=9606 GN=SLC6A9 PE=1 SV=3	78.2	4	10	4	8	17.0743084	16.93156815	17.13570976	17.99369049	17.97140884	18.02647972
ACYP2	P14621	-0.94821167	Acylphosphatase-2 OS=Homo sapiens OX=9606	11.1	1	1	1	9	14.418643	13.24674034	14.11958981	15.02756023	14.82376575	14.77828217

			GN=ACYP2 PE=1 SV=2											
PLSCR1	O15162	-0.938346863	Phospholipid scramblase 1 OS=Homo sapiens OX=9606 GN=PLSCR1 PE=1 SV=1	35	2	8	2	8	19.71565437	19.50881577	19.81127357	20.52611732	20.54510117	20.77956581
CTH	P32929	-0.931770325	Cystathionine gamma-lyase OS=Homo sapiens OX=9606 GN=CTH PE=1 SV=3	44.5	7	14	7	21	19.8190937	19.43338966	19.88762474	20.63644028	20.62756157	20.67141724
LMCD1	Q9NZU5	-0.929750443	LIM and cysteine-rich domains protein 1 OS=Homo sapiens OX=9606 GN=LMCD1 PE=1 SV=1	40.8	7	12	7	25	18.53943062	18.56941032	18.65228462	19.33864975	19.36024666	19.85148048
ATL1	Q8WXF7	-0.923484802	Atlastin-1 OS=Homo sapiens OX=9606 GN=ATL1 PE=1 SV=1	63.5	6	11	6	12	16.29131508	15.83861351	16.36324501	17.05324745	17.08472443	17.12565613
AP1G2	O75843	-0.922719955	AP-1 complex subunit gamma-like 2 OS=Homo sapiens OX=9606	87.1	2	5	2	2	15.28410149	14.58637047	15.05748367	15.80120277	15.9246273	15.97028542

			GN=AP1G2 PE=1 SV=1											
ISG15	P05161	-0.92189916	Ubiquitin-like protein ISG15 OS=Homo sapiens OX=9606 GN=ISG15 PE=1 SV=5	17.9	6	28	6	48	22.88762474	22.55215454	22.96895027	23.72858238	23.75938797	23.68645668
DOCK8	Q8NF50	-0.910210609	Dedicator of cytokinesis protein 8 OS=Homo sapiens OX=9606 GN=DOCK8 PE=1 SV=3	238.4	1	4	2	1	14.89178371	14.76279736	14.94306374	16.41204071	15.26956463	15.6466713
PROSER2	Q86WR7	-0.903856277	Proline and serine-rich protein 2 OS=Homo sapiens OX=9606 GN=PROSER2 PE=1 SV=2	45.8	4	6	4	9	15.83861351	15.61252308	15.79360294	16.65228462	16.62399483	16.68002892
PSMB9	P28065	-0.899153392	Proteasome subunit beta type-9 OS=Homo sapiens OX=9606 GN=PSMB9 PE=1 SV=2	23.3	4	12	4	23	19.33864975	19.15560913	19.3794117	20.19460297	20.15807724	20.21845055
TMEM164	Q5U3C3	-0.887097994	Transmembrane protein 164 OS=Homo sapiens	33.5	2	4	2	8	13.05832958	12.89368057	13.10656261	13.97477341	13.85330963	13.89178371

			OX=9606 GN=TMEM164 PE=1 SV=1											
MYL9	P24844	- 0.8829561 87	Myosin regulatory light polypeptide 9 OS=Homo sapiens OX=9606 GN=MYL9 PE=1 SV=4	19.8	3	119	9	62	18.887624 74	18.648778 92	18.902421 95	19.698799 13	19.582332 61	19.806562 42
SLC6A15	Q9H2J7	- 0.8769648 87	Sodium-dependent neutral amino acid transporter B(0)AT2 OS=Homo sapiens OX=9606 GN=SLC6A15 PE=1 SV=1	81.8	2	2	2	3	13.451210 98	13.501836 78	13.901244 16	14.550746 92	14.464035 03	14.470404 62
ARFGEF3	Q5TH69	- 0.8758811 95	Brefeldin A-inhibited guanine nucleotide-exchange protein 3 OS=Homo sapiens OX=9606 GN=ARFGEF3 PE=1 SV=3	240. 5	5	6	5	3	16.038318 63	16.177185 06	16.486206 05	17.031873 7	17.074308 4	17.223171 23
STC2	O76061	- 0.8718039 19	Stanniocalcin-2 OS=Homo sapiens OX=9606 GN=STC2 PE=1 SV=1	33.2	2	9	2	13	14.649480 82	14.532599 45	15.127672 2	15.589300 16	15.612523 08	15.723340 99

CDCP1	Q9H5V8	-0.871765773	CUB domain-containing protein 1 OS=Homo sapiens OX=9606 GN=CDCP1 PE=1 SV=3	92.9	7	12	7	9	17.22317123	17.31451225	17.27866745	18.15065956	18.16545677	18.11553192
ICAM1	P05362	-0.866923014	Intercellular adhesion molecule 1 OS=Homo sapiens OX=9606 GN=ICAM1 PE=1 SV=2	57.8	17	41	17	37	21.93876457	21.6496563	22.00195885	22.7055645	22.71607208	22.76951218
ALDH1B1	P30837	-0.865966161	Aldehyde dehydrogenase X, mitochondrial OS=Homo sapiens OX=9606 GN=ALDH1B1 PE=1 SV=4	57.2	16	47	17	33	21.93156815	21.50688171	21.94948959	22.68217468	22.65839958	22.64526367
NUDT19	A8MXV4	-0.865623474	Acyl-coenzyme A diphosphatase NUDT19 OS=Homo sapiens OX=9606 GN=NUDT19 PE=1 SV=1	42.2	4	7	4	17	17.89652252	17.48934555	17.93156815	18.6132431	18.64877892	18.65228462
RIGI	O95786	-0.862653732	Antiviral innate immune response receptor RIG-I	106.5	19	39	19	26	19.93156815	19.65228462	20.00195885	20.6971035	20.6971035	20.77956581

			OS=Homo sapiens OX=9606 GN=RIGI PE=1 SV=2											
ANKRD52	Q8NB46	-0.8525575	Serine/threonine-protein phosphatase 6 regulatory ankyrin repeat subunit C OS=Homo sapiens OX=9606 GN=ANKRD52 PE=1 SV=3	115	5	11	5	6	17.23257065	17.21371269	17.44151688	18.15065956	18.10021019	18.19460297
TRIM14	Q14142	-0.845648448	Tripartite motif-containing protein 14 OS=Homo sapiens OX=9606 GN=TRIM14 PE=1 SV=2	49.7	8	11	8	20	17.59514046	17.55074692	17.64526367	18.46562958	18.44960022	18.41286659
GMPR	P36959	-0.843374888	GMP reductase 1 OS=Homo sapiens OX=9606 GN=GMPR PE=1 SV=1	37.4	4	15	6	24	17.64526367	17.40041161	17.65927124	18.34948921	18.39623642	18.48934555
CD63	P08962	-0.843251546	CD63 antigen OS=Homo sapiens OX=9606 GN=CD63 PE=1 SV=2	25.6	2	3	2	12	14.43827248	13.83368111	14.19844532	14.9246273	14.79867458	15.27685165

GBP4	Q96PP9	-0.838513692	Guanylate-binding protein 4 OS=Homo sapiens OX=9606 GN=GBP4 PE=1 SV=2	73.1	14	27	14	23	19.84842682	19.58600426	19.93156815	20.59149361	20.61862946	20.67141724
MAP2	P11137	-0.835258484	Microtubule-associated protein 2 OS=Homo sapiens OX=9606 GN=MAP2 PE=1 SV=4	199.4	7	12	7	5	17.96013832	17.86060143	18.06381607	18.73396873	18.80183411	18.85452843
PARP14	Q460N5	-0.833214442	Protein mono-ADP-ribosyltransferase PARP14 OS=Homo sapiens OX=9606 GN=PARP14 PE=1 SV=3	202.7	28	55	28	17	20.21845055	20.14569283	20.31007957	21.02917862	21.06907272	21.07561493
GNAO1	P09471	-0.832821528	Guanine nucleotide-binding protein G(o) subunit alpha OS=Homo sapiens OX=9606 GN=GNAO1 PE=1 SV=4	40	5	26	7	21	17.98815155	17.89652252	18.04793358	18.7634449	18.78596306	18.88166428
DIP2C	Q9Y2E4	-0.831298192	Disco-interacting protein 2 homolog C OS=Homo sapiens OX=9606	170.7	4	12	7	4	13.14370251	13.08679962	13.52649879	14.14370251	13.9476366	14.15955639

			GN=DIP2C PE=1 SV=2												
C1orf122	Q6ZSJ8	-0.82783254	Uncharacterized protein C1orf122 OS=Homo sapiens OX=9606 GN=C1orf122 PE=1 SV=2	11.5	1	2	1	10	14.6325407	14.22881889	14.56271935	15.5111351	15.27685165	15.11958981
ACSS1	Q9NUB1	-0.824299812	Acetyl-coenzyme A synthetase 2-like, mitochondrial OS=Homo sapiens OX=9606 GN=ACSS1 PE=1 SV=2	74.8	2	5	2	4	14.51422119	13.96578407	14.18301487	15.11146164	14.99258423	15.0318737
SQOR	Q9Y6N5	-0.824021657	Sulfide:quinone oxidoreductase, mitochondrial OS=Homo sapiens OX=9606 GN=SQOR PE=1 SV=1	49.9	19	49	19	44	21.25925636	21.049263	21.29333687	22.03925705	22.01563263	22.01903152
CYP27A1	Q02318	-0.820075989	Sterol 26-hydroxylase, mitochondrial OS=Homo sapiens OX=9606 GN=CYP27A1 PE=1 SV=1	60.2	3	5	3	8	14.44475651	14.22881889	14.63820934	15.2585659	15.2585659	15.25488091

CPS1	P31327	-0.818698883	Carbamoyl-phosphate synthase [ammonia], mitochondrial OS=Homo sapiens OX=9606 GN=CPS1 PE=1 SV=2	164.8	11	17	14	11	17.97140884	17.7923336	18.11553192	18.78596306	18.7634449	18.78596306
TYMP	P19971	-0.81779925	Thymidine phosphorylase OS=Homo sapiens OX=9606 GN=TYMP PE=1 SV=2	49.9	3	4	3	11	14.45763779	14.45121098	14.68825054	15.45442772	15.37856579	15.21750355
DOK1	Q99704	-0.802922885	Docking protein 1 OS=Homo sapiens OX=9606 GN=DOK1 PE=1 SV=1	52.4	1	1	1	2	15.35810184	14.83861351	15.29131508	15.96803665	15.93617821	15.99258423
UBA7	P41226	-0.79635493	Ubiquitin-like modifier-activating enzyme 7 OS=Homo sapiens OX=9606 GN=UBA7 PE=1 SV=2	111.6	12	31	12	16	19.25349617	18.97140884	19.29445839	19.96013832	19.96013832	19.98815155
MGLL	Q99685	-0.794882456	Monoglyceride lipase OS=Homo sapiens OX=9606	33.2	3	7	3	15	16.38700676	16.19075012	16.69370461	17.17523766	17.30563354	17.17523766

			GN=MGLL PE=1 SV=2											
HERC5	Q9UII4	-0.792896907	E3 ISG15--protein ligase HERC5 OS=Homo sapiens OX=9606 GN=HERC5 PE=1 SV=2	116.8	8	18	8	9	18.29670143	18.42111206	18.45763779	19.20895767	19.1481781	19.19700623
NDFIP1	Q9BT67	-0.788342476	NEDD4 family-interacting protein 1 OS=Homo sapiens OX=9606 GN=NDFIP1 PE=1 SV=1	24.9	1	1	1	3	13.1308918	12.77930927	13.67727947	14.07012081	13.76279736	14.11958981
HLA-H	P01893	-0.785024007	Putative HLA class I histocompatibility antigen, alpha chain H OS=Homo sapiens OX=9606 GN=HLA-H PE=5 SV=3	40.9	2	42	9	39	17.38363647	17.23257065	17.65927124	18.11553192	18.11044312	18.40457535
C18orf21	Q32NC0	-0.781308174	UPF0711 protein C18orf21 OS=Homo sapiens OX=9606 GN=C18orf21 PE=1 SV=1	24.8	2	3	2	10	13.58637047	12.84549046	13.3303566	14.15165043	13.90124416	14.05324745

SAMD9L	Q8IVG5	-0.765397708	Sterile alpha motif domain-containing protein 9-like OS=Homo sapiens OX=9606 GN=SAMD9L PE=1 SV=2	184.4	1	3	2	1	13.6325407	13.69913864	13.73131943	14.41204071	14.47040462	14.47674656
GLI2	P10070	-0.758027395	Zinc finger protein GLI2 OS=Homo sapiens OX=9606 GN=GLI2 PE=1 SV=4	167.7	1	3	2	1	15.39204884	15.07012081	15.60095787	16.18495369	16.01454353	16.13771248
GCA	P28676	-0.757404327	Grancalcin OS=Homo sapiens OX=9606 GN=GCA PE=1 SV=2	24	4	12	4	18	18.7038765	18.56941032	18.7634449	19.22552681	19.45763779	19.62578011
SULF1	Q8IWU6	-0.756983757	Extracellular sulfatase Sulf-1 OS=Homo sapiens OX=9606 GN=SULF1 PE=1 SV=1	101	2	5	2	2	14.50804234	14.23631287	14.80372715	15.51730061	15.19844532	15.1032877
TMEM106C	Q9BVX2	-0.752633095	Transmembrane protein 106C OS=Homo sapiens OX=9606 GN=TMEM106C PE=1 SV=1	27.9	1	2	1	7	13.75238037	14.15955639	14.03617382	14.81378078	14.72600555	14.66622353

SH3BGRL 2	Q9UJC5	- 0.7522513 07	SH3 domain-binding glutamic acid-rich-like protein 2 OS=Homo sapiens OX=9606 GN=SH3BGRL2 PE=1 SV=2	12.3	2	3	2	27	15.421933 17	15.167418 48	15.412040 71	16.109422 68	15.884647 37	16.264076 23
GPNMB	Q14956	- 0.7453950 25	Transmembrane glycoprotein NMB OS=Homo sapiens OX=9606 GN=GPNMB PE=1 SV=2	63.9	4	13	4	8	19.439489 36	19.227878 57	19.443542 48	20.264991 76	20.107891 08	19.974212 65
HMGA2	P52926	- 0.7437601 09	High mobility group protein HMGI-C OS=Homo sapiens OX=9606 GN=HMGA2 PE=1 SV=1	11.8	2	3	2	28	16.291315 08	15.875077 25	16.179132 46	16.860601 43	16.773139 95	16.943063 74
SECTM1	Q8WV N6	- 0.7424885 43	Secreted and transmembrane protein 1 OS=Homo sapiens OX=9606 GN=SECTM1 PE=1 SV=2	27	1	2	1	4	12.435669 9	12.154818 53	12.965784 07	13.075144 77	13.283377 65	13.425215 72
HNMT	P50135	- 0.7396583 56	Histamine N-methyltransferase OS=Homo sapiens	33.3	8	17	8	38	19.069072 72	18.905363 08	19.213712 69	19.829971 31	19.725671 77	19.851480 48

			OX=9606 GN=HNMT PE=1 SV=1											
TTC33	Q6PID6	-0.732195854	Tetratricopeptide repeat protein 33 OS=Homo sapiens OX=9606 GN=TTC33 PE=1 SV=2	29.4	3	7	3	9	15.25488091	14.85817528	15.15560913	15.78851414	15.773139	15.90359974
LCP1	P13796	-0.728040059	Plastin-2 OS=Homo sapiens OX=9606 GN=LCP1 PE=1 SV=6	70.2	4	46	12	19	17.09506798	16.73396873	17.17523766	17.74057198	17.74057198	17.7072506
CCPG1	Q9ULG6	-0.727968216	Cell cycle progression protein 1 OS=Homo sapiens OX=9606 GN=CCPG1 PE=1 SV=3	87.3	10	23	10	14	18.73065567	18.25580406	18.82687187	19.37729454	19.29221344	19.32772827
PRELID1	Q9Y255	-0.722567558	PRELI domain-containing protein 1, mitochondrial OS=Homo sapiens OX=9606 GN=PRELID1 PE=1 SV=1	25.2	1	2	1	3	14.57459354	13.9476366	14.38532352	14.96126842	15.14370251	14.97028542
SAMD9	Q5K651	-0.714235942	Sterile alpha motif domain-containing protein 9 OS=Homo	184.2	25	49	26	20	21.07561493	20.81127357	21.14569283	21.73065567	21.73065567	21.71397781

			sapiens OX=9606 GN=SAMD9 PE=1 SV=1											
DSC3	Q14574	-0.707529068	Desmocollin-3 OS=Homo sapiens OX=9606 GN=DSC3 PE=1 SV=3	99.9	6	9	6	10	15.69097996	15.68002987	16.12161446	16.5819664	16.43664742	16.59659767
BCAM	P50895	-0.706419627	Basal cell adhesion molecule OS=Homo sapiens OX=9606 GN=BCAM PE=1 SV=2	67.4	14	41	14	27	19.97421265	19.57680893	20.02917862	20.60059547	20.58233261	20.51653099
PDCD1LG2	Q9BQ51	-0.706401825	Programmed cell death 1 ligand 2 OS=Homo sapiens OX=9606 GN=PDCD1LG2 PE=1 SV=2	30.9	2	6	2	9	17.87267494	17.38363647	17.7923336	18.44556427	18.42111206	18.30117416
PLSCR4	Q9NRQ2	-0.704267502	Phospholipid scramblase 4 OS=Homo sapiens OX=9606 GN=PLSCR4 PE=1 SV=2	37	2	5	2	7	16.69370461	16.43501854	16.66622353	17.2877121	17.32333565	17.29670143
STX11	O75558	-0.704051971	Syntaxin-11 OS=Homo sapiens OX=9606	33.2	3	6	3	13	17.44960022	17.22317123	17.58049393	18.0743084	18.16545677	18.12565613

			GN=STX11 PE=1 SV=1											
LRRC41	Q15345	-0.699740728	Leucine-rich repeat-containing protein 41 OS=Homo sapiens OX=9606 GN=LRRC41 PE=1 SV=3	88.6	9	17	9	12	19.05059242	18.75694656	19.1180687	19.72567177	19.63466835	19.66448975
FKBP7	Q9Y680	-0.697052638	Peptidyl-prolyl cis-trans isomerase FKBP7 OS=Homo sapiens OX=9606 GN=FKBP7 PE=1 SV=2	25.8	9	22	9	34	20.40665436	20.01563263	20.34299469	20.99507141	20.88016891	20.98119926
PANK3	Q9H999	-0.693639755	Pantothenate kinase 3 OS=Homo sapiens OX=9606 GN=PANK3 PE=1 SV=1	41.1	1	9	4	10	12.66622353	12.73343945	12.58637047	13.69913864	13.24822426	13.11958981
WWP1	Q9H0M0	-0.693286578	NEDD4-like E3 ubiquitin-protein ligase WWP1 OS=Homo sapiens OX=9606 GN=WWP1 PE=1 SV=1	105.1	2	21	7	9	12.4893465	12.31344986	12.44604969	13.14529514	13.07848454	13.10492611

STAT1	P42224	-0.692441305	Signal transducer and activator of transcription 1-alpha/beta OS=Homo sapiens OX=9606 GN=STAT1 PE=1 SV=2	87.3	32	132	32	52	22.94413757	22.66709137	22.9207077	23.54037857	23.54037857	23.52850342
SQSTM1	Q13501	-0.691071192	Sequestosome-1 OS=Homo sapiens OX=9606 GN=SQSTM1 PE=1 SV=1	47.7	6	28	6	26	19.22787857	19.04526901	19.23491096	19.89208031	19.840765	19.84842682
PCBD1	P61457	-0.689531326	Pterin-4-alpha-carbinolamine dehydratase OS=Homo sapiens OX=9606 GN=PCBD1 PE=1 SV=2	12	2	5	2	22	18.0318737	18.0743084	18.05324745	18.60964012	19.04526901	18.5731144
DGLUCY	Q7Z3D6	-0.687506676	D-glutamate cyclase, mitochondrial OS=Homo sapiens OX=9606 GN=DGLUCY PE=1 SV=2	66.4	3	3	3	5	15.45121098	15.25488091	15.64385605	16.23069572	16.07848358	16.10328865
PTGR1	Q14914	-0.678469976	Prostaglandin reductase 1 OS=Homo sapiens OX=9606	35.8	11	39	11	45	20.86514091	20.65403366	20.90242195	21.50688171	21.47753716	21.47258759

			GN=PTGR1 PE=1 SV=2											
TXNRD1	Q16881	-0.676429749	Thioredoxin reductase 1, cytoplasmic OS=Homo sapiens OX=9606 GN=TXNRD1 PE=1 SV=3	70.9	28	188	29	59	24.18706894	23.8190937	24.24626541	24.73892403	24.72858238	24.81421089
PSAT1	Q9Y617	-0.673563639	Phosphoserine aminotransferase OS=Homo sapiens OX=9606 GN=PSAT1 PE=1 SV=2	40.4	22	116	23	75	23.28206635	22.9207077	23.25060844	23.77956581	23.79946518	23.89504242
LETMD1	Q6P1Q0	-0.673497836	LETM1 domain-containing protein 1 OS=Homo sapiens OX=9606 GN=LETMD1 PE=1 SV=1	41.8	1	1	1	3	13.00140858	12.39070606	12.78953362	13.67727947	13.22279453	13.30206776
ACADS	P16219	-0.67326355	Short-chain specific acyl-CoA dehydrogenase, mitochondrial OS=Homo sapiens OX=9606 GN=ACADS PE=1 SV=1	44.3	2	5	2	6	16.19460297	16.37517548	16.43989563	16.99920654	17.05324745	16.97701073

TNFAIP8L1	Q8WVP5	-0.672844251	Tumor necrosis factor alpha-induced protein 8-like protein 1 OS=Homo sapiens OX=9606 GN=TNFAIP8L1 PE=1 SV=2	20.8	2	5	2	18	13.55074692	13.16427803	13.27029514	13.92925835	13.85330963	14.22128487
GPBP1L1	Q9HC44	-0.672465642	Vasculin-like protein 1 OS=Homo sapiens OX=9606 GN=GPBP1L1 PE=1 SV=1	52.3	2	2	2	5	11.88417053	12.10000515	12.61194706	12.84156418	12.79360294	12.97835255
TGFBR1	P36897	-0.670111338	TGF-beta receptor type-1 OS=Homo sapiens OX=9606 GN=TGFBR1 PE=1 SV=1	55.9	6	13	6	15	18.08472443	17.86060143	18.04260063	18.67658997	18.68346024	18.6382103
IFRD1	O00458	-0.667743047	Interferon-related developmental regulator 1 OS=Homo sapiens OX=9606 GN=IFRD1 PE=1 SV=4	50.2	3	6	3	9	15.43175888	15.3056345	15.81878185	16.21750259	16.2041893	16.13771248
SLC1A4	P43007	-0.666260401	Neutral amino acid transporter A OS=Homo sapiens OX=9606	55.7	9	27	9	26	20.97421265	20.5731144	21.02242279	21.51171494	21.51171494	21.54510117

			GN=SLC1A4 PE=1 SV=1											
ENAH	Q8N8S7	-0.662318548	Protein enabled homolog OS=Homo sapiens OX=9606 GN=ENAH PE=1 SV=2	66.5	18	69	19	34	21.15807724	20.87267494	21.18252945	21.63644028	21.65403366	21.90976334
CEP162	Q5TB80	-0.662183762	Centrosomal protein of 162 kDa OS=Homo sapiens OX=9606 GN=CEP162 PE=1 SV=2	161.8	2	3	2	1	11.94398022	12.32192802	12.65060329	12.89178371	13.20151138	12.80976772
GRPEL2	Q8TAA5	-0.660852432	GrpE protein homolog 2, mitochondrial OS=Homo sapiens OX=9606 GN=GRPEL2 PE=1 SV=1	25.4	3	4	3	15	14.07848454	14.63820934	14.56866837	15.12363625	14.96126842	15.18301487
FAIM	Q9NVQ4	-0.654917399	Fas apoptotic inhibitory molecule 1 OS=Homo sapiens OX=9606 GN=FAIM PE=1 SV=1	20.2	2	3	2	9	14.21371174	14.44475651	14.04473591	14.98370647	14.9476366	14.73661327
METAP2	P50579	-0.64470164	Methionine aminopeptidase 2 OS=Homo sapiens OX=9606	52.9	13	35	13	35	20.26499176	19.96013832	20.26499176	20.7875576	20.77152824	20.86514091

			GN=METAP2 PE=1 SV=1											
SLC38A1	Q9H2H9	-0.644071579	Sodium-coupled neutral amino acid symporter 1 OS=Homo sapiens OX=9606 GN=SLC38A1 PE=1 SV=1	54	3	4	3	6	17.80498886	17.16545677	17.72067261	18.36238861	18.15560913	18.10533524
TAP2	Q03519	-0.64114507	Antigen peptide transporter 2 OS=Homo sapiens OX=9606 GN=TAP2 PE=1 SV=1	75.6	13	43	13	23	20.1332016	19.80970573	20.06907272	20.61862946	20.66275215	20.65403366
STX3	Q13277	-0.639233907	Syntaxin-3 OS=Homo sapiens OX=9606 GN=STX3 PE=1 SV=3	33.1	7	13	7	21	19.05324745	18.53564072	19.12817574	19.57496262	19.52037239	19.53943062
WLS	Q5T9L3	-0.63866361	Protein wntless homolog OS=Homo sapiens OX=9606 GN=WLS PE=1 SV=2	62.2	4	5	4	8	17.58049393	17.33210564	17.58783531	18.12565613	18.17523766	18.11553192
QNG1	Q5T6V5	-0.63848877	Queuosine 5'-phosphate N-glycosylase/hydrolase OS=Homo sapiens OX=9606	39	12	33	12	37	20.82687187	20.94592476	20.93876457	21.42726326	21.45261955	21.7471447

			GN=QNG1 PE=1 SV=1											
CPE	P16870	-0.638007164	Carboxypeptidase E OS=Homo sapiens OX=9606 GN=CPE PE=1 SV=1	53.1	1	2	1	3	14.50804234	14.13570976	14.2877121	14.81378078	15.06592083	14.96578407
PANK1	Q8TE04	-0.637008349	Pantothenate kinase 1 OS=Homo sapiens OX=9606 GN=PANK1 PE=1 SV=2	64.3	1	7	3	5	13.04473591	13.16427803	13.38532352	13.67727947	13.97477341	13.85330963
KATNAL1	Q9BW62	-0.636942546	Katanin p60 ATPase-containing subunit A-like 1 OS=Homo sapiens OX=9606 GN=KATNAL1 PE=1 SV=1	55.4	10	29	11	28	19.10277557	18.7923336	19.26040459	19.72567177	19.64526367	19.69540596
CALHM5	Q8N5C1	-0.63445282	Calcium homeostasis modulator protein 5 OS=Homo sapiens OX=9606 GN=CALHM5 PE=1 SV=1	35.1	2	3	2	7	17.52802658	17.18495369	17.58049393	18.04260063	17.99369049	18.16054153
SLC41A3	Q96GZ6	-0.633909225	Solute carrier family 41 member 3 OS=Homo sapiens	54.7	2	3	2	6	16.06381607	16.19844437	16.42685509	16.86060143	16.79867363	16.93156815

			OX=9606 GN=SLC41A3 PE=1 SV=2											
AHNAK2	Q8IVF2	-0.632943471	Protein AHNAK2 OS=Homo sapiens OX=9606 GN=AHNAK2 PE=1 SV=2	616.2	5	13	7	6	17.01017761	17.04260063	17.04260063	17.58049393	17.67314339	17.74057198
LACTB2	Q53H82	-0.632263184	Endoribonuclease LACTB2 OS=Homo sapiens OX=9606 GN=LACTB2 PE=1 SV=2	32.8	10	18	10	46	20.34299469	20.05589676	20.40665436	20.77956581	20.88016891	21.04260063
NDUFAF8	A1L188	-0.630886714	NADH dehydrogenase [ubiquinone] 1 alpha subcomplex assembly factor 8 OS=Homo sapiens OX=9606 GN=NDUFAF8 PE=1 SV=1	7.8	1	2	1	11	13.84352875	14.07012081	14.01889515	14.74714375	14.45121098	14.62685013
WNT5A	P41221	-0.630590439	Protein Wnt-5a OS=Homo sapiens OX=9606 GN=WNT5A PE=1 SV=2	42.3	3	6	4	13	15.42521572	15.33734322	15.74976444	16.1634922	15.84352875	16.39707375

RAB3D	O95716	-0.626317978	Ras-related protein Rab-3D OS=Homo sapiens OX=9606 GN=RAB3D PE=1 SV=1	24.3	1	28	5	27	13.72067165	13.99258423	14.20609856	14.3442955	14.83861351	14.61539936
PTP4A1	Q93096	-0.626164118	Protein tyrosine phosphatase type IVA 1 OS=Homo sapiens OX=9606 GN=PTP4A1 PE=1 SV=2	19.8	1	9	5	29	16.04473495	16.19460297	16.13370323	16.66622353	16.44960022	17.13570976
LAP3	P28838	-0.624540965	Cytosol aminopeptidase OS=Homo sapiens OX=9606 GN=LAP3 PE=1 SV=3	56.1	28	114	28	66	23.48000526	23.03086472	23.48000526	23.94055748	23.91342163	24.01051903
TAP1	Q03518	-0.624010722	Antigen peptide transporter 1 OS=Homo sapiens OX=9606 GN=TAP1 PE=1 SV=3	80.9	12	33	12	21	20.29893875	20.10789108	20.37517548	20.84230042	20.88016891	20.93156815
EIF5A2	Q9GZV4	-0.622922262	Eukaryotic translation initiation factor 5A-2 OS=Homo sapiens OX=9606 GN=EIF5A2 PE=1 SV=3	16.8	3	91	8	52	19.01835251	18.71732903	19.01835251	19.54698563	19.47159576	19.60421944

GFPT2	O94808	-0.622334798	Glutamine--fructose-6-phosphate aminotransferase [isomerizing] 2 OS=Homo sapiens OX=9606 GN=GFPT2 PE=1 SV=3	76.9	8	27	13	21	17.77956581	17.36666298	17.86060143	18.30117416	18.22317123	18.34948921
CALD1	Q05682	-0.620914459	Caldesmon OS=Homo sapiens OX=9606 GN=CALD1 PE=1 SV=3	93.2	48	295	49	49	23.85756874	23.59832573	23.84804535	24.416996	24.28206635	24.46762085
GBP2	P32456	-0.62074852	Guanylate-binding protein 2 OS=Homo sapiens OX=9606 GN=GBP2 PE=1 SV=3	67.2	9	36	13	26	20.26499176	20.00195885	20.39623642	20.88762474	20.79550743	20.84230042
IRF9	Q00978	-0.620384852	Interferon regulatory factor 9 OS=Homo sapiens OX=9606 GN=IRF9 PE=1 SV=1	43.7	7	15	7	24	18.91122818	18.416996	18.92868042	19.29670143	19.42930794	19.39204979
PML	P29590	-0.619992574	Protein PML OS=Homo sapiens OX=9606 GN=PML PE=1 SV=3	97.5	24	60	24	27	20.77152824	20.53564072	20.80341148	21.23022652	21.2877121	21.45261955

TP53I3	Q53FA7	-0.619509379	Quinone oxidoreductase PIG3 OS=Homo sapiens OX=9606 GN=TP53I3 PE=1 SV=2	35.5	9	31	9	27	21.18857956	20.95304871	21.27639771	21.69285393	21.72234154	21.86135864
STX12	Q86Y82	-0.617559433	Syntaxin-12 OS=Homo sapiens OX=9606 GN=STX12 PE=1 SV=1	31.6	6	18	6	26	19.18978691	18.72733498	19.00744438	19.48148346	19.59696198	19.69879913
BAZ2A	Q9UIF9	-0.617179871	Bromodomain adjacent to zinc finger domain protein 2A OS=Homo sapiens OX=9606 GN=BAZ2A PE=1 SV=4	211.1	9	14	9	5	17.54321289	17.30563354	17.60240936	18.15065956	18.02647972	18.12565613
UBE2L6	O14933	-0.616341273	Ubiquitin/ISG15-conjugating enzyme E2 L6 OS=Homo sapiens OX=9606 GN=UBE2L6 PE=1 SV=4	17.8	4	8	4	37	17.81753349	17.7471447	17.95446968	18.4616394	18.48542023	18.42111206
HLA-B	P01889	-0.614527384	HLA class I histocompatibility antigen, B alpha chain	40.4	3	50	9	33	18.91415215	18.43745995	18.94306374	19.43948936	19.36666298	19.33210564

			OS=Homo sapiens OX=9606 GN=HLA-B PE=1 SV=3											
BPGM	P07738	-0.613222122	Bisphosphoglycerate mutase OS=Homo sapiens OX=9606 GN=BPGM PE=1 SV=2	30	8	11	8	37	18.97980499	18.68688393	19.0372467	19.533741	19.49911308	19.51074791
PDE5A	O76074	-0.609534899	cGMP-specific 3',5'-cyclic phosphodiesterase OS=Homo sapiens OX=9606 GN=PDE5A PE=1 SV=2	99.9	3	5	3	4	14.17523766	13.87267494	14.418643	14.81878185	14.79360294	14.6827755
DDAH1	O94760	-0.608717601	N(G),N(G)-dimethylarginine dimethylaminohydrolase 1 OS=Homo sapiens OX=9606 GN=DDAH1 PE=1 SV=3	31.1	10	41	11	52	21.47753716	21.20060158	21.53564072	21.92795753	21.90242195	22.20955276
RND3	P61587	-0.60589091	Rho-related GTP-binding protein RhoE OS=Homo sapiens OX=9606	27.4	5	15	5	27	18.99092293	18.58783531	19.21608353	19.68174553	19.36879539	19.56197357

			GN=RND3 PE=1 SV=1											
DNMT3A	Q9Y6K1	-0.604459127	DNA (cytosine-5)-methyltransferase 3A OS=Homo sapiens OX=9606 GN=DNMT3A PE=1 SV=4	101.8	1	3	2	3	13.31628132	13.19537258	13.3442955	14.01017857	13.72067165	13.93847656
HLA-C	P10321	-0.604284286	HLA class I histocompatibility antigen, C alpha chain OS=Homo sapiens OX=9606 GN=HLA-C PE=1 SV=3	40.6	4	65	11	40	21.04260063	20.77152824	21.04260063	21.47753716	21.58691978	21.60512543
SLC38A2	Q96QD8	-0.603313446	Sodium-coupled neutral amino acid symporter 2 OS=Homo sapiens OX=9606 GN=SLC38A2 PE=1 SV=2	56	4	12	4	12	18.99920654	18.90536308	19.26728058	19.58966637	19.62220955	19.76991463
SNAPC1	Q16533	-0.603291194	snRNA-activating protein complex subunit 1 OS=Homo sapiens OX=9606 GN=SNAPC1 PE=1 SV=1	43	3	4	3	8	16.3109684	15.82625008	16.45442772	16.7471447	16.66622353	16.98815155

ABCD1	P33897	-0.601881345	ATP-binding cassette sub-family D member 1 OS=Homo sapiens OX=9606 GN=ABCD1 PE=1 SV=2	82.9	5	9	5	6	16.44313812	15.90124416	16.35810089	16.84842682	16.84842682	16.81127357
USP11	P51784	-0.601640066	Ubiquitin carboxyl-terminal hydrolase 11 OS=Homo sapiens OX=9606 GN=USP11 PE=1 SV=3	109.7	25	65	27	32	21.79550743	21.44758415	21.83845901	22.26785278	22.26212692	22.35649109
HLA-A	P04439	-0.59937795	HLA class I histocompatibility antigen, A alpha chain OS=Homo sapiens OX=9606 GN=HLA-A PE=1 SV=2	40.8	3	75	9	32	22.11743546	21.69285393	22.02917862	22.54980659	22.53564072	22.55215454
IFI44	Q8TCB0	-0.598285039	Interferon-induced protein 44 OS=Homo sapiens OX=9606 GN=IFI44 PE=1 SV=2	50.5	3	4	3	6	14.15955639	14.2585659	14.51422119	14.88226128	14.8087635	15.03617382
PARP12	Q9H0J9	-0.597171783	Protein mono-ADP-ribosyltransferase PARP12 OS=Homo sapiens OX=9606	79	7	12	7	12	18.13069153	17.86060143	18.01017761	18.66275215	18.60602951	18.52420425

			GN=PARP12 PE=1 SV=1											
CASP4	P49662	-0.5950737	Caspase-4 OS=Homo sapiens OX=9606 GN=CASP4 PE=1 SV=1	43.2	12	38	12	37	20.4873848	20.19460297	20.55449867	20.93156815	20.99507141	21.09506798
SOD2	P04179	-0.590027491	Superoxide dismutase [Mn], mitochondrial OS=Homo sapiens OX=9606 GN=SOD2 PE=1 SV=3	24.7	8	31	8	50	22.5937748	22.31561852	22.58691978	23.09345627	23.12533951	23.04759979
PLEKHF1	Q96S99	-0.584883372	Pleckstrin homology domain-containing family F member 1 OS=Homo sapiens OX=9606 GN=PLEKHF1 PE=1 SV=3	31.2	1	6	2	11	15.55374908	15.31981277	15.46722317	15.86785793	16.06802177	16.15955544
CROT	Q9UKG9	-0.583362579	Peroxisomal carnitine O-octanoyltransferase OS=Homo sapiens OX=9606 GN=CROT PE=1 SV=2	70.1	10	28	10	20	20.00195885	19.63112068	20.06907272	20.47753716	20.47753716	20.49716568

IRGQ	Q8WZA9	-0.582703273	Immunity-related GTPase family Q protein OS=Homo sapiens OX=9606 GN=IRGQ PE=1 SV=1	62.7	18	51	18	43	20.18252945	19.97421265	20.20657539	20.66275215	20.64526367	20.80341148
SP110	Q9HB58	-0.578086217	Sp110 nuclear body protein OS=Homo sapiens OX=9606 GN=SP110 PE=1 SV=5	78.3	6	13	6	13	16.84842682	16.46722412	16.83614922	17.2877121	17.22317123	17.37517548
B2M	P61769	-0.577702204	Beta-2-microglobulin OS=Homo sapiens OX=9606 GN=B2M PE=1 SV=1	13.7	4	20	4	41	22.01563263	21.75123596	22.23899651	22.75531769	22.67357445	22.31007957
ACAA1	P09110	-0.577473958	3-ketoacyl-CoA thiolase, peroxisomal OS=Homo sapiens OX=9606 GN=ACAA1 PE=1 SV=2	44.3	14	43	14	49	21.27070618	20.91706848	21.34299469	21.71397781	21.72234154	21.82687187
PSMB8	P28062	-0.577169418	Proteasome subunit beta type-8 OS=Homo sapiens OX=9606 GN=PSMB8 PE=1 SV=3	30.3	8	25	8	30	20.40665436	20.1332016	20.56383705	21.00881195	20.88762474	20.93876457

DNAJB1	P25685	-0.570112864	DnaJ homolog subfamily B member 1 OS=Homo sapiens OX=9606 GN=DNAJB1 PE=1 SV=4	38	16	44	17	48	22.13946152	21.80341148	22.13946152	22.62756157	22.5308857	22.63422585
PPP1R9A	Q9ULJ8	-0.569355647	Neurabin-1 OS=Homo sapiens OX=9606 GN=PPP1R9A PE=1 SV=2	123.3	7	23	8	9	16.48306084	16.11756134	16.39037132	16.96578407	16.86060143	16.87267494
IFI35	P80217	-0.566052755	Interferon-induced 35 kDa protein OS=Homo sapiens OX=9606 GN=IFI35 PE=1 SV=5	31.5	14	39	14	55	21.25349617	21.02242279	21.23022652	21.72650337	21.73065567	21.7471447
CHCHD2	Q9Y6H1	-0.564954122	Coiled-coil-helix-coiled-coil-helix domain-containing protein 2 OS=Homo sapiens OX=9606 GN=CHCHD2 PE=1 SV=1	15.5	2	6	2	15	17.51267815	17.69370461	17.72733498	18.09506798	18.30563354	18.22787857
GBA2	Q9HCG7	-0.563829422	Non-lysosomal glucosylceramidase OS=Homo sapiens OX=9606	104.6	1	3	1	2	13.773139	13.3442955	13.89178371	14.3233366	14.11146164	14.26590824

			GN=GBA2 PE=1 SV=2											
YIPF3	Q9GZM5	-0.563341777	Protein YIPF3 OS=Homo sapiens OX=9606 GN=YIPF3 PE=1 SV=1	38.2	2	2	2	4	15.74976444	15.63820934	16.03831863	16.34775925	16.21181297	16.55674553
CD274	Q9NZQ7	-0.562699	Programmed cell death 1 ligand 1 OS=Homo sapiens OX=9606 GN=CD274 PE=1 SV=1	33.3	7	15	7	31	19.12817574	19.08731842	19.32553291	19.72400665	19.6399765	19.86514091
ALDH1L2	Q3SY69	-0.561096827	Mitochondrial 10-formyltetrahydrofolate dehydrogenase OS=Homo sapiens OX=9606 GN=ALDH1L2 PE=1 SV=2	101.7	29	66	29	36	21.60059547	21.35380173	21.62756157	22.0886116	22.05920219	22.11743546
ADAMTS1	Q9UHI8	-0.560727437	A disintegrin and metalloproteinase with thrombospondin motifs 1 OS=Homo sapiens OX=9606 GN=ADAMTS1 PE=1 SV=4	105.3	3	4	3	3	16.69370461	16.47199249	16.6067524	17.18495369	17.08472443	17.18495369

NMI	Q13287	-0.558916728	N-myc-interactor OS=Homo sapiens OX=9606 GN=NMI PE=1 SV=2	35	14	30	14	45	20.85756874	20.61862946	20.92433739	21.38047028	21.34840775	21.34840775
TMLHE	Q9NVH6	-0.555681547	Trimethyllysine dioxygenase, mitochondrial OS=Homo sapiens OX=9606 GN=TMLHE PE=1 SV=1	49.5	3	5	3	7	15.9970026	15.68825054	15.9059515	16.30028152	16.35121536	16.6067524
L3HYPDH	Q96EM0	-0.555514018	Trans-3-hydroxy-L-proline dehydratase OS=Homo sapiens OX=9606 GN=L3HYPDH PE=1 SV=2	38.1	7	17	7	24	20.05589676	19.86966705	20.05589676	20.45763779	20.54510117	20.64526367
FBXO6	Q9NRD1	-0.554331144	F-box only protein 6 OS=Homo sapiens OX=9606 GN=FBXO6 PE=1 SV=1	33.9	2	5	2	8	14.23631287	14.22881889	14.41204071	14.85330963	14.75759792	14.92925835
ERICH5	Q6P6B1	-0.550865809	Glutamate-rich protein 5 OS=Homo sapiens OX=9606 GN=ERICH5 PE=1 SV=1	39.9	10	20	10	29	18.80498886	18.69029808	18.71397781	19.28996468	19.37729454	19.19460297

EVI5L	Q96CN4	-0.550478935	EVI5-like protein OS=Homo sapiens OX=9606 GN=EVI5L PE=1 SV=1	91.3	5	7	5	7	15.25118637	15.11553097	15.52955246	15.80120277	15.98370647	15.76279736
EPB41L4B	Q9H329	-0.547568639	Band 4.1-like protein 4B OS=Homo sapiens OX=9606 GN=EPB41L4B PE=1 SV=2	99.7	1	1	1	1	15.19075108	15.00580025	15.20227718	15.73131943	15.74451828	15.56569672
CBLB	Q13191	-0.547074636	E3 ubiquitin-protein ligase CBL-B OS=Homo sapiens OX=9606 GN=CBLB PE=1 SV=2	109.4	1	5	3	4	14.11146164	13.95673943	13.64385605	14.51422119	14.58049393	14.2585659
MBLAC1	A4D2B0	-0.546921412	Metallo-beta-lactamase domain-containing protein 1 OS=Homo sapiens OX=9606 GN=MBLAC1 PE=1 SV=1	27.2	4	9	4	29	15.33385372	15.47357845	15.45442772	15.91298294	15.88226128	16.10737991
CEBPB	P17676	-0.546559016	CCAAT/enhancer-binding protein beta OS=Homo sapiens OX=9606 GN=CEBPB PE=1 SV=2	36.1	4	8	4	17	18.35380173	18.29670143	18.45362473	18.94592476	18.79867363	18.99920654

TRMT10C	Q7L0Y3	-0.544663111	tRNA methyltransferase 10 homolog C OS=Homo sapiens OX=9606 GN=TRMT10C PE=1 SV=2	47.3	13	33	13	36	20.61862946	20.45763779	20.688591	21.17035484	21.10789108	21.12060165
PARD3B	Q8TEW8	-0.542641958	Partitioning defective 3 homolog B OS=Homo sapiens OX=9606 GN=PARD3B PE=1 SV=2	132.4	4	8	4	4	17.98259163	17.76668358	18.11553192	18.51267815	18.55074692	18.42930794
CEBPD	P49716	-0.542048772	CCAAT/enhancer-binding protein delta OS=Homo sapiens OX=9606 GN=CEBPD PE=1 SV=2	28.4	2	2	2	7	14.66066456	14.27321243	14.50183678	14.92925835	14.96126842	15.17133331
NEXN	Q0ZGT2	-0.539415359	Nexilin OS=Homo sapiens OX=9606 GN=NEXN PE=1 SV=1	80.6	21	48	21	27	21.9207077	21.5308857	21.93156815	22.33483696	22.31007957	22.35649109
LAMTOR5	O43504	-0.536428452	Ragulator complex protein LAMTOR5 OS=Homo sapiens OX=9606 GN=LAMTOR5 PE=1 SV=1	9.6	1	4	1	22	16.39037132	16.57754707	16.43338966	16.99920654	17.17523766	16.83614922

TMEM201	Q5SNT2	-0.53631719	Transmembrane protein 201 OS=Homo sapiens OX=9606 GN=TMEM201 PE=1 SV=1	72.2	8	16	8	16	18.25580406	18.02647972	18.3794117	18.6971035	18.7038765	18.86966705
CSTB	P04080	-0.535862605	Cystatin-B OS=Homo sapiens OX=9606 GN=CSTB PE=1 SV=2	11.1	5	28	5	62	20.08212852	20.01563263	20.20657539	20.6971035	20.7875576	20.42726326
TMCC3	Q9ULS5	-0.535289764	Transmembrane and coiled-coil domain protein 3 OS=Homo sapiens OX=9606 GN=TMCC3 PE=1 SV=3	53.8	1	1	1	2	15.26956463	15.03617382	15.47674656	15.72600555	15.84107304	15.82127571
DAPK1	P53355	-0.534267426	Death-associated protein kinase 1 OS=Homo sapiens OX=9606 GN=DAPK1 PE=1 SV=6	159.9	6	16	7	5	17.04260063	16.95446968	17.31451225	17.65228462	17.61683655	17.64526367
PRKAR1B	P31321	-0.534228643	cAMP-dependent protein kinase type I-beta regulatory subunit OS=Homo sapiens OX=9606	43	3	12	5	18	16.7471447	16.55374908	16.73396873	17.13570976	17.27866745	17.22317123

			GN=PRKAR1B PE=1 SV=4											
GNPDA1	P46926	-0.533706665	Glucosamine-6-phosphate deaminase 1 OS=Homo sapiens OX=9606 GN=GNPDA1 PE=1 SV=1	32.6	7	32	10	57	21.56383705	21.41183281	21.66709137	21.99507141	22.03925705	22.20955276
APOL2	Q9BQE5	-0.533676147	Apolipoprotein L2 OS=Homo sapiens OX=9606 GN=APOL2 PE=1 SV=2	37.1	8	26	8	28	19.57865334	19.17523766	19.53943062	19.97421265	19.97421265	19.94592476
PSG7	Q13046	-0.532819112	Pregnancy-specific beta-1-glycoprotein 7 OS=Homo sapiens OX=9606 GN=PSG7 PE=1 SV=3	47	2	2	2	4	16.19844437	16.26040459	16.40706825	16.89652252	16.7072506	16.86060143
F11R	Q9Y624	-0.532755852	Junctional adhesion molecule A OS=Homo sapiens OX=9606 GN=F11R PE=1 SV=1	32.6	2	4	2	9	13.43827248	13.51422119	13.41204071	13.93847656	13.87267494	14.15165043
CALB2	P22676	-0.532495499	Calretinin OS=Homo sapiens OX=9606 GN=CALB2 PE=1 SV=2	31.5	8	24	9	36	20.45763779	20.12060165	20.50688171	20.88016891	20.96013832	20.84230042

TAGLN	Q01995	-0.532393773	Transgelin OS=Homo sapiens OX=9606 GN=TAGLN PE=1 SV=4	22.6	16	230	16	71	24.05258369	23.74919128	24.05258369	24.46138954	24.46762085	24.5225296
CMC1	Q7Z7K0	-0.53162384	COX assembly mitochondrial protein homolog OS=Homo sapiens OX=9606 GN=CMC1 PE=1 SV=1	12.5	2	6	2	25	15.97028542	16.32509422	16.14370155	16.78596306	16.62399483	16.62399483
PON2	Q15165	-0.529996872	Serum paraoxonase/arylesterase 2 OS=Homo sapiens OX=9606 GN=PON2 PE=1 SV=4	39.4	8	28	8	37	21.1332016	20.688591	21.16422844	21.53564072	21.46263885	21.57773209
HELZ2	Q9BYK8	-0.529680888	3'-5' exoribonuclease HELZ2 OS=Homo sapiens OX=9606 GN=HELZ2 PE=1 SV=7	322.1	28	49	28	12	20.10789108	20.10789108	20.09506798	20.65403366	20.64526367	20.60059547
MELTF	P08582	-0.529661179	Melanotransferrin OS=Homo sapiens OX=9606 GN=MELTF PE=1 SV=2	80.2	12	27	12	18	19.97421265	20.06907272	19.97421265	20.54510117	20.50688171	20.55449867

NOTCH1	P46531	-0.527713776	Neurogenic locus notch homolog protein 1 OS=Homo sapiens OX=9606 GN=NOTCH1 PE=1 SV=4	272.3	5	10	5	2	17.19460297	17.0743084	17.14569283	17.65228462	17.7072506	17.6382103
PPCDC	Q96CD2	-0.526897748	Phosphopantothenoylcysteine decarboxylase OS=Homo sapiens OX=9606 GN=PPCDC PE=1 SV=2	22.4	1	1	1	3	14.48305988	14.67176247	14.84842777	15.19844532	15.11958981	15.26590824
FBLIM1	Q8WUP2	-0.525311152	Filamin-binding LIM protein 1 OS=Homo sapiens OX=9606 GN=FBLIM1 PE=1 SV=2	40.6	6	14	6	19	17.93156815	17.919981	18.0795269	18.37517548	18.49716568	18.63466835
PTGFRN	Q9P2B2	-0.524857203	Prostaglandin F2 receptor negative regulator OS=Homo sapiens OX=9606 GN=PTGFRN PE=1 SV=2	98.5	18	45	18	23	20.94592476	20.72234154	20.92433739	21.3698616	21.37517548	21.42213821
HSPB1	P04792	-0.523998896	Heat shock protein beta-1 OS=Homo sapiens OX=9606	22.8	13	76	13	81	22.64306259	22.6496563	22.5892086	23.23899651	23.14258003	23.07234764

			GN=HSPB1 PE=1 SV=2												
HDDC2	Q7Z4H3	-0.523962021	5'-deoxynucleotidase HDDC2 OS=Homo sapiens OX=9606 GN=HDDC2 PE=1 SV=1	23.4	3	7	3	20	17.76668358	17.50494385	17.7536869	18.22787857	18.15560913	18.21371269
TBC1D17	Q9HA65	-0.523300807	TBC1 domain family member 17 OS=Homo sapiens OX=9606 GN=TBC1D17 PE=1 SV=3	72.6	4	5	4	10	16.86060143	16.50028229	16.93156815	17.416996	17.18495369	17.26040459
ARHGAP26	Q9UNA1	-0.523209254	Rho GTPase-activating protein 26 OS=Homo sapiens OX=9606 GN=ARHGAP26 PE=1 SV=1	92.2	4	6	4	5	16.72067261	16.23818016	16.59514046	17.06381607	16.95446968	17.10533524
CPM	P14384	-0.522795359	Carboxypeptidase M OS=Homo sapiens OX=9606 GN=CPM PE=1 SV=2	50.5	5	12	5	12	18.80183411	18.87267494	18.76668358	19.3709259	19.36452866	19.27412415
FSCN1	Q16658	-0.52221489	Fascin OS=Homo sapiens OX=9606	54.5	27	124	27	63	23.84804535	23.56383705	23.89504242	24.27497673	24.26785278	24.33073997

			GN=FSCN1 PE=1 SV=3											
ERAP1	Q9NZ08	-0.521045049	Endoplasmic reticulum aminopeptidase 1 OS=Homo sapiens OX=9606 GN=ERAP1 PE=1 SV=3	107.2	12	22	12	14	19.56755447	19.1801033	19.61144257	20.00195885	19.96013832	19.96013832
ASNS	P08243	-0.520899455	Asparagine synthetase [glutamine-hydrolyzing] OS=Homo sapiens OX=9606 GN=ASNS PE=1 SV=4	64.3	12	28	12	26	21.69285393	21.43237114	21.71397781	22.10789108	22.12060165	22.17340851
ADAMTS5	Q9UNA0	-0.520685196	A disintegrin and metalloproteinase with thrombospondin motifs 5 OS=Homo sapiens OX=9606 GN=ADAMTS5 PE=1 SV=2	101.7	4	6	4	4	15.90124416	15.48620701	15.60964012	16.23631287	16.14370155	16.17913246
ARL6	Q9H0F7	-0.519568761	ADP-ribosylation factor-like protein 6 OS=Homo sapiens OX=9606 GN=ARL6 PE=1 SV=1	21.1	3	7	3	20	17.94306374	17.84842682	18.0795269	18.43745995	18.44151688	18.55074692

DYSF	O75923	-0.518851598	Dysferlin OS=Homo sapiens OX=9606 GN=DYSF PE=1 SV=1	237.1	14	39	17	9	18.4616394	18.24190903	18.58049393	18.94306374	18.94306374	18.95446968
GNPNAT1	Q96EK6	-0.518789927	Glucosamine 6-phosphate N-acetyltransferase OS=Homo sapiens OX=9606 GN=GNPNAT1 PE=1 SV=1	20.7	3	6	3	33	20.59149361	20.4873848	20.75531769	21.02917862	21.10789108	21.25349617
GALNT3	Q14435	-0.518762589	Polypeptide N-acetylgalactosaminyltransferase 3 OS=Homo sapiens OX=9606 GN=GALNT3 PE=1 SV=2	72.6	8	12	8	15	18.40872765	18.32333565	18.45362473	18.91415215	18.88762474	18.9401989
NPEPL1	Q8NDH3	-0.518062592	Probable aminopeptidase NPEPL1 OS=Homo sapiens OX=9606 GN=NPEPL1 PE=1 SV=3	55.8	7	11	7	20	18.26040459	18.05324745	18.40457535	18.70049477	18.78276825	18.78915215
ETS2	P15036	-0.5180041	Protein C-ets-2 OS=Homo sapiens OX=9606 GN=ETS2 PE=1 SV=1	53	1	2	1	2	13.64385605	13.82376575	13.53867435	13.95673943	14.27321243	14.3303566

SNX15	Q9NRS6	-0.514431	Sorting nexin-15 OS=Homo sapiens OX=9606 GN=SNX15 PE=1 SV=1	38.3	10	17	10	34	19.23491096	18.7602005	19.18252945	19.51267815	19.62042046	19.58783531
SLC49A4	Q96SL1	-0.51346906	Solute carrier family 49 member 4 OS=Homo sapiens OX=9606 GN=SLC49A4 PE=1 SV=1	52.1	1	1	1	1	14.45121098	14.19075108	14.45763779	14.81878185	15.05324745	14.76797771
FOXF2	Q12947	-0.512814204	Forkhead box protein F2 OS=Homo sapiens OX=9606 GN=FOXF2 PE=1 SV=2	46	1	2	1	2	16.98815155	17.04260063	17.30563354	17.63112068	17.54321289	17.70049477
TGFBI	Q15582	-0.512503942	Transforming growth factor-beta-induced protein ig-h3 OS=Homo sapiens OX=9606 GN=TGFBI PE=1 SV=1	74.6	4	8	4	6	13.99258423	13.83368111	14.01889515	14.30206776	14.60385799	14.47674656
HACL1	Q9UJ83	-0.512390137	2-hydroxyacyl-CoA lyase 1 OS=Homo sapiens OX=9606 GN=HACL1 PE=1 SV=2	63.7	10	26	10	21	20.52611732	20.26499176	20.60059547	21.00195885	20.93876457	20.98815155

BDH1	Q02338	-0.512282689	D-beta-hydroxy-butyrate dehydrogenase, mitochondrial OS=Homo sapiens OX=9606 GN=BDH1 PE=1 SV=3	38.1	6	12	6	21	18.72733498	18.48934555	18.6557827	19.06381607	19.18495369	19.16054153
GLS	O94925	-0.511808395	Glutaminase kidney isoform, mitochondrial OS=Homo sapiens OX=9606 GN=GLS PE=1 SV=1	73.4	20	99	20	45	23.00881195	22.67357445	23.12060165	23.45512962	23.39100075	23.49228287
ACOT7	O00154	-0.509405772	Cytosolic acyl coenzyme A thioester hydrolase OS=Homo sapiens OX=9606 GN=ACOT7 PE=1 SV=3	41.8	16	73	16	48	22.08537483	21.98119926	22.19760513	22.62086868	22.59149361	22.58003426
SERAC1	Q96JX3	-0.50883166	Protein SERAC1 OS=Homo sapiens OX=9606 GN=SERAC1 PE=1 SV=1	74.1	3	5	3	4	17.37517548	17.11553192	17.416996	17.7072506	17.84230042	17.88464737
DUS3L	Q96G46	-0.508825938	tRNA-dihydrouridine(47) synthase [NAD(P)(+)]-like	72.5	7	15	7	17	17.56569672	17.27866745	17.44960022	17.87267494	17.94306374	18.00470352

			OS=Homo sapiens OX=9606 GN=DUS3L PE=1 SV=2											
ARHGEF6	Q15052	-0.506605148	Rho guanine nucleotide exchange factor 6 OS=Homo sapiens OX=9606 GN=ARHGEF6 PE=1 SV=2	87.4	1	5	2	3	15.58637047	15.52955246	15.73925304	16.1316967	15.9059515	16.33734322
NF2	P35240	-0.506203969	Merlin OS=Homo sapiens OX=9606 GN=NF2 PE=1 SV=1	69.6	13	35	14	22	21.06907272	20.79550743	21.0886116	21.4873848	21.47753716	21.50688171
CGRRF1	Q99675	-0.505602837	Cell growth regulator with RING finger domain protein 1 OS=Homo sapiens OX=9606 GN=CGRRF1 PE=1 SV=1	38.2	1	2	1	5	14.75238037	14.55674553	14.64385605	15.01017857	15.17913151	15.28048038
TMEM168	Q9H0V1	-0.505472501	Transmembrane protein 168 OS=Homo sapiens OX=9606 GN=TMEM168 PE=1 SV=2	79.7	3	3	3	6	14.15955639	13.89178371	14.13570976	14.45121098	14.58049393	14.67176247

EPB41L5	Q9HCM4	-0.50499026	Band 4.1-like protein 5 OS=Homo sapiens OX=9606 GN=EPB41L5 PE=1 SV=3	81.8	11	19	11	24	19.32991982	19.17767334	19.40872765	19.78436661	19.85300446	19.79392052
ERMP1	Q7Z2K6	-0.504749298	Endoplasmic reticulum metallopeptidase 1 OS=Homo sapiens OX=9606 GN=ERMP1 PE=1 SV=2	100.2	6	11	6	7	18.94592476	18.6417408	19.01017761	19.3899498	19.33429146	19.38784981
IFIT1	P09914	-0.503210068	Antiviral innate immune response effector IFIT1 OS=Homo sapiens OX=9606 GN=IFIT1 PE=1 SV=2	55.3	16	38	16	39	21.3698616	21.19460297	21.45261955	21.78356743	21.81518936	21.92795753
DPP7	Q9UHL4	-0.500212987	Dipeptidyl peptidase 2 OS=Homo sapiens OX=9606 GN=DPP7 PE=1 SV=3	54.3	8	16	8	22	19.10021019	18.8994751	19.21845055	19.57126427	19.54510117	19.60240936
MGST1	P10620	-0.499895732	Microsomal glutathione S-transferase 1 OS=Homo sapiens OX=9606 GN=MGST1 PE=1 SV=1	17.6	4	13	4	41	20.2877121	20.04260063	20.25349617	20.59149361	20.688591	20.80341148

LRRC20	Q8TCA0	-0.4998703	Leucine-rich repeat-containing protein 20 OS=Homo sapiens OX=9606 GN=LRRC20 PE=1 SV=1	20.5	5	11	5	42	18.02106667	17.94877815	18.30117416	18.58049393	18.77313995	18.416996
LACTB	P83111	-0.499422709	Serine beta-lactamase-like protein LACTB, mitochondrial OS=Homo sapiens OX=9606 GN=LACTB PE=1 SV=2	60.7	14	49	14	27	21.40145493	21.05589676	21.47258759	21.77152824	21.79153824	21.86514091
TCIRG1	Q13488	-0.498764674	V-type proton ATPase 116 kDa subunit a 3 OS=Homo sapiens OX=9606 GN=TCIRG1 PE=1 SV=3	92.9	13	26	14	20	20.26499176	20.40665436	20.35380173	20.77956581	20.80341148	20.93876457
CELSR2	Q9HCU4	-0.497696877	Cadherin EGF LAG seven-pass G-type receptor 2 OS=Homo sapiens OX=9606 GN=CELSR2 PE=1 SV=1	317.3	2	2	2	1	13.98370647	13.98370647	14.03617382	14.61539936	14.29490757	14.58637047

FAM177A 1	Q8N128	- 0.4974721 27	Protein FAM177A1 OS=Homo sapiens OX=9606 GN=FAM177A1 PE=1 SV=1	23.7	6	11	6	40	18.937328 34	18.595140 46	18.872674 94	19.258106 23	19.289964 68	19.349489 21
NT5DC3	Q86UY8	- 0.4958775 84	5'-nucleotidase do-main-containing protein 3 OS=Homo sapiens OX=9606 GN=NT5DC3 PE=1 SV=1	63.4	9	17	9	19	18.673143 39	18.323335 65	18.750419 62	19.130691 53	19.053247 45	19.050592 42
LGMN	Q99538	- 0.4957644 14	Legumain OS=Homo sapiens OX=9606 GN=LGMN PE=1 SV=1	49.4	3	7	3	7	16.884647 37	16.798673 63	17.095067 98	17.433389 66	17.465629 58	17.366662 98
PRRC2B	Q5JSZ5	- 0.4949639 64	Protein PRRC2B OS=Homo sapiens OX=9606 GN=PRRC2B PE=1 SV=2	242. 8	25	57	26	16	19.693704 61	19.467620 85	19.812841 42	20.206575 39	20.082128 52	20.170354 84
SKIC3	Q6PGP7	- 0.4926586 15	Superkiller complex protein 3 OS=Homo sapiens OX=9606 GN=SKIC3 PE=1 SV=1	175. 4	25	61	25	18	21.008811 95	20.787557 6	21.022422 79	21.447584 15	21.406654 36	21.442529 68

FYTTD1	Q96QD9	-0.492580414	UAP56-interacting factor OS=Homo sapiens OX=9606 GN=FYTTD1 PE=1 SV=3	35.8	9	20	9	36	19.83306313	19.78116798	19.92433739	20.39623642	20.29893875	20.32113457
PIP5K1C	O60331	-0.492157936	Phosphatidylinositol 4-phosphate 5-kinase type-1 gamma OS=Homo sapiens OX=9606 GN=PIP5K1C PE=1 SV=2	73.2	4	5	4	6	16.1634922	15.96126842	16.25672531	16.59368324	16.59805298	16.66622353
ATP13A3	Q9H7F0	-0.491295497	Polyamine-transporting ATPase 13A3 OS=Homo sapiens OX=9606 GN=ATP13A3 PE=1 SV=4	138	9	17	9	9	18.6132431	18.16545677	18.52420425	18.89356232	18.93445015	18.94877815
NDUFC2	O95298	-0.490976969	NADH dehydrogenase [ubiquinone] 1 subunit C2 OS=Homo sapiens OX=9606 GN=NDUFC2 PE=1 SV=1	14.2	5	8	5	24	19.30340576	19.31451225	19.62756157	19.92578697	20.02917862	19.7634449
TOR1B	O14657	-0.490976969	Torsin-1B OS=Homo sapiens OX=9606	38	9	15	9	33	21.0886116	20.7634449	21.22434998	21.51653099	21.49716568	21.53564072

			GN=TOR1B PE=1 SV=2											
SKIC2	Q15477	-0.490571976	Superkiller complex protein 2 OS=Homo sapiens OX=9606 GN=SKIC2 PE=1 SV=3	137.7	21	42	21	24	20.72234154	20.71397781	20.87267494	21.21252441	21.23608017	21.33210564
SLC35A4	L0R6Q1	-0.489985784	SLC35A4 upstream open reading frame protein OS=Homo sapiens OX=9606 GN=SLC35A4 PE=1 SV=1	11.1	2	6	2	17	16.60240936	16.57754707	16.84842682	17.33210564	17.22317123	16.94306374
SAMHD1	Q9Y3Z3	-0.48747762	Deoxynucleoside triphosphate tri-phosphohydrolase SAMHD1 OS=Homo sapiens OX=9606 GN=SAMHD1 PE=1 SV=2	72.2	34	109	34	61	23.37782478	23.03925705	23.37782478	23.72858238	23.74919128	23.77956581
GPAT3	Q53EU6	-0.486165682	Glycerol-3-phosphate acyltransferase 3 OS=Homo sapiens OX=9606 GN=GPAT3 PE=1 SV=2	48.7	3	6	3	8	14.41204071	14.25118637	14.3442955	14.82873154	14.79867458	14.83861351

STAT5A	P42229	-0.485525449	Signal transducer and activator of transcription 5A OS=Homo sapiens OX=9606 GN=STAT5A PE=1 SV=1	90.6	1	24	11	15	14.61539936	14.59805202	14.87747574	15.13169575	15.22505665	15.19075108
CIAO2A	Q9H5X1	-0.484223684	Cytosolic iron-sulfur assembly component 2A OS=Homo sapiens OX=9606 GN=CIAO2A PE=1 SV=1	18.3	2	7	2	13	15.9903698	15.69913864	16.01454353	16.28048134	16.33908463	16.53715706
PLPP3	O14495	-0.483933767	Phospholipid phosphatase 3 OS=Homo sapiens OX=9606 GN=PLPP3 PE=1 SV=1	35.1	1	1	1	2	16.94306374	16.66622353	16.96578407	17.38363647	17.25118637	17.39204979
PSME2	Q9UL46	-0.483647664	Proteasome activator complex subunit 2 OS=Homo sapiens OX=9606 GN=PSME2 PE=1 SV=4	27.4	15	50	15	67	23.40405655	23.09506798	23.46762085	23.77956581	23.82880974	23.80931282

PTTG1IP	P53801	-0.482524236	Pituitary tumor-transforming gene 1 protein-interacting protein OS=Homo sapiens OX=9606 GN=PTTG1IP PE=1 SV=1	20.3	2	6	2	8	18.05854225	17.91415215	18.17523766	18.53564072	18.53183746	18.52802658
GDI1	P31150	-0.481585821	Rab GDP dissociation inhibitor alpha OS=Homo sapiens OX=9606 GN=GDI1 PE=1 SV=2	50.6	12	101	20	54	22.40405655	22.25925636	22.57773209	22.88576508	22.83460617	22.96543121
RAB2B	Q8WUD1	-0.481524785	Ras-related protein Rab-2B OS=Homo sapiens OX=9606 GN=RAB2B PE=1 SV=1	24.2	4	55	11	67	18.90829849	18.78596306	19.16300011	19.42726326	19.41493225	19.4596405
PSMG1	O95456	-0.480514526	Proteasome assembly chaperone 1 OS=Homo sapiens OX=9606 GN=PSMG1 PE=1 SV=1	32.8	4	9	4	19	19.61683655	19.4636364	19.82065201	20.17035484	20.00195885	20.17035484
HEBP2	Q9Y5Z4	-0.480272929	Heme-binding protein 2 OS=Homo sapiens OX=9606 GN=HEBP2 PE=1 SV=1	22.9	7	21	7	43	19.91269112	19.52802658	19.87867355	20.26499176	20.23022652	20.26499176

TXNDC16	Q9P2K2	-0.47991689	Thioredoxin domain-containing protein 16 OS=Homo sapiens OX=9606 GN=TXNDC16 PE=1 SV=4	93.5	2	2	2	3	16.38869095	16.15758324	16.54170227	16.7471447	16.919981	16.86060143
DCTD	P32321	-0.47776413	Deoxycytidylate deaminase OS=Homo sapiens OX=9606 GN=DCTD PE=1 SV=2	20	3	4	3	23	17.54321289	17.44960022	17.65927124	17.98259163	17.93732834	18.16545677
MYLK	Q15746	-0.477371216	Myosin light chain kinase, smooth muscle OS=Homo sapiens OX=9606 GN=MYLK PE=1 SV=4	210.6	44	106	45	27	21.52611732	21.40145493	21.55917549	21.95659828	21.9671917	21.99507141
SLC7A1	P30825	-0.475264231	High affinity cationic amino acid transporter 1 OS=Homo sapiens OX=9606 GN=SLC7A1 PE=1 SV=1	67.6	3	6	3	4	18.80813408	18.52420425	18.78915215	19.15807724	19.13570976	19.25349617
CASP7	P55210	-0.474659602	Caspase-7 OS=Homo sapiens OX=9606	34.3	4	13	4	17	17.94306374	17.54321289	17.94306374	18.26499176	18.26499176	18.32333565

			GN=CASP7 PE=1 SV=1											
PPP1R9B	Q96SB3	-0.474432627	Neurabin-2 OS=Homo sapiens OX=9606 GN=PPP1R9B PE=1 SV=3	89.3	21	48	22	34	20.38574409	20.05589676	20.49716568	20.8190937	20.7634449	20.77956581
PDLIM5	Q96HC4	-0.473990758	PDZ and LIM domain protein 5 OS=Homo sapiens OX=9606 GN=PDLIM5 PE=1 SV=5	63.9	22	59	22	37	22.02242279	21.61414146	21.97421265	22.37517548	22.2877121	22.3698616
ACAP2	Q15057	-0.473798752	Arf-GAP with coiled-coil, ANK repeat and PH domain-containing protein 2 OS=Homo sapiens OX=9606 GN=ACAP2 PE=1 SV=3	88	21	51	21	31	20.42726326	20.25349617	20.43745995	20.81127357	20.81127357	20.91706848
MAP3K20	Q9NYL2	-0.470984777	Mitogen-activated protein kinase kinase kinase 20 OS=Homo sapiens OX=9606 GN=MAP3K20 PE=1 SV=3	91.1	15	33	15	25	20.61862946	20.63644028	20.73065567	21.09506798	21.05589676	21.247715

DGUOK	Q16854	-0.470650991	Deoxyguanosine kinase, mitochondrial OS=Homo sapiens OX=9606 GN=DGUOK PE=1 SV=2	32	2	2	2	5	16.94306374	16.88464737	16.99920654	17.55074692	17.2877121	17.40041161
RPP25	Q9BUL9	-0.469973564	Ribonuclease P protein subunit p25 OS=Homo sapiens OX=9606 GN=RPP25 PE=1 SV=1	20.6	1	2	1	7	15.3056345	15.38532352	15.5597353	15.94991779	15.67452335	16.03617287
ZNF91	Q05481	-0.469964663	Zinc finger protein 91 OS=Homo sapiens OX=9606 GN=ZNF91 PE=1 SV=2	137.1	2	6	4	15	16.21181297	15.87507725	16.10328865	16.58343506	16.47040558	16.54623222
RP2	O75695	-0.469335556	Protein XRP2 OS=Homo sapiens OX=9606 GN=RP2 PE=1 SV=4	39.6	11	23	11	33	21.42213821	21.02917862	21.42213821	21.77555275	21.72234154	21.78356743
BTN2A1	Q7KYR7	-0.46928978	Butyrophilin subfamily 2 member A1 OS=Homo sapiens OX=9606 GN=BTN2A1 PE=1 SV=3	59.6	3	5	3	8	18.76668358	18.74386215	18.78915215	19.16300011	19.19940376	19.34516335

DHRS4	Q9BTZ2	-0.469156265	Dehydrogenase/reductase SDR family member 4 OS=Homo sapiens OX=9606 GN=DHRS4 PE=1 SV=3	29.5	6	17	6	27	20.05589676	19.8190937	20.1332016	20.38574409	20.44758415	20.58233261
WWC2	Q6AWC2	-0.46897761	Protein WWC2 OS=Homo sapiens OX=9606 GN=WWC2 PE=1 SV=2	133.8	3	6	3	4	14.45763779	14.22881889	14.47040462	14.81878185	14.7045517	15.04046059
ELOVL5	Q9NYP7	-0.46828715	Very long chain fatty acid elongase 5 OS=Homo sapiens OX=9606 GN=ELOVL5 PE=1 SV=1	35.3	2	4	2	6	14.89652157	15.15955639	15.15560913	15.6296978	15.45121098	15.53563976
STK38L	Q9Y2H1	-0.465614001	Serine/threonine-protein kinase 38-like OS=Homo sapiens OX=9606 GN=STK38L PE=1 SV=3	54	2	6	4	10	14.19075108	13.99258423	14.23631287	14.46403503	14.73131943	14.62113571
HEG1	Q9ULI3	-0.464183172	Protein HEG homolog 1 OS=Homo sapiens OX=9606	147.4	6	13	6	6	18.82065201	18.5731144	18.80183411	19.13069153	19.16300011	19.29445839

			GN=HEG1 PE=1 SV=3											
NIT2	Q9NQR4	-0.462287267	Omega-amidase NIT2 OS=Homo sapiens OX=9606 GN=NIT2 PE=1 SV=1	30.6	13	36	13	62	21.25349617	21.08212852	21.33756065	21.57773209	21.60964012	21.87267494
CDO1	Q16878	-0.459777514	Cysteine dioxygenase type 1 OS=Homo sapiens OX=9606 GN=CDO1 PE=1 SV=2	23	1	5	2	10	13.52649879	13.51422119	13.39874363	13.98370647	13.83368111	14.00140858
GOLGA4	Q13439	-0.459506353	Golgin subfamily A member 4 OS=Homo sapiens OX=9606 GN=GOLGA4 PE=1 SV=1	261	87	221	88	42	23.52850342	23.23753929	23.57542419	23.86702919	23.88576508	23.9671917
RDX	P35241	-0.459487915	Radixin OS=Homo sapiens OX=9606 GN=RDX PE=1 SV=1	68.5	19	161	36	53	23.20657539	22.90976334	23.31007957	23.57542419	23.57542419	23.65403366
PKN3	Q6P5Z2	-0.458972931	Serine/threonine-protein kinase N3 OS=Homo sapiens OX=9606	99.4	11	22	12	18	18.64526367	18.48934555	18.77313995	19.08731842	19.07169342	19.12565613

			GN=PKN3 PE=1 SV=1											
ATPAF1	Q5TC12	-0.458009084	ATP synthase mitochondrial F1 complex assembly factor 1 OS=Homo sapiens OX=9606 GN=ATPAF1 PE=1 SV=1	36.4	2	2	2	5	17.44151688	17.11553192	17.44960022	17.7602005	17.70049477	17.919981
PIGG	Q5H8A4	-0.457553228	GPI ethanolamine phosphate transferase 2, catalytic subunit OS=Homo sapiens OX=9606 GN=PIGG PE=1 SV=1	108.1	5	9	6	7	18.2041893	17.90829849	18.30117416	18.52420425	18.55824089	18.7038765
EML1	O00423	-0.457496643	Echinoderm microtubule-associated protein-like 1 OS=Homo sapiens OX=9606 GN=EML1 PE=1 SV=3	89.8	11	20	12	15	18.90242195	18.84230042	19.01017761	19.3730526	19.35810089	19.39623642
MOSPD1	Q9UJG1	-0.457260132	Motile sperm domain-containing protein 1 OS=Homo sapiens OX=9606	24.1	2	5	2	9	17.81753349	17.65927124	17.95446968	18.26040459	18.23257065	18.31007957

			GN=MOSPD1 PE=1 SV=1											
XYLB	O75191	-0.457032522	Xylulose kinase OS=Homo sapiens OX=9606 GN=XYLB PE=1 SV=3	58.3	8	17	8	22	18.45763779	18.34948921	18.47357941	18.83614922	18.83306313	18.98259163
SIX5	Q8N196	-0.455598195	Homeobox protein SIX5 OS=Homo sapiens OX=9606 GN=SIX5 PE=1 SV=3	74.5	2	8	3	5	15.31274128	14.96578407	15.3442955	15.58930016	15.73131943	15.66899586
SER-PINH1	P50454	-0.453521093	Serpin H1 OS=Homo sapiens OX=9606 GN=SER-PINH1 PE=1 SV=2	46.4	21	142	21	63	24.49228287	24.10149384	24.51050758	24.88576508	24.78456688	24.79451561
KCTD7	Q96MP8	-0.452184677	BTB/POZ domain-containing protein KCTD7 OS=Homo sapiens OX=9606 GN=KCTD7 PE=1 SV=1	33.1	3	3	3	10	16.77313995	16.66622353	17.02106667	17.23257065	17.29670143	17.2877121
SLC25A4	P12235	-0.452010473	ADP/ATP translocase 1 OS=Homo sapiens OX=9606 GN=SLC25A4 PE=1 SV=4	33	4	71	16	62	19.06118202	19.04526901	19.17767334	19.44758415	19.49716568	19.69540596

ACOT1	Q86TX2	-0.451941172	Acyl-coenzyme A thioesterase 1 OS=Homo sapiens OX=9606 GN=ACOT1 PE=1 SV=1	46.2	12	36	12	42	21.03590584	20.87267494	21.10149384	21.43237114	21.416996	21.51653099
NOP-CHAP1	Q8N5I9	-0.451640447	NOP protein chaperone 1 OS=Homo sapiens OX=9606 GN=NOPCHAP1 PE=1 SV=2	20.1	3	5	3	15	16.8237648	16.77313995	17.15560913	17.39204979	17.32333565	17.39204979
GPT2	Q8TD30	-0.448780696	Alanine aminotransferase 2 OS=Homo sapiens OX=9606 GN=GPT2 PE=1 SV=1	57.9	5	15	7	18	19.17279816	18.83614922	19.14071083	19.45763779	19.49326134	19.54510117
SLC35A2	P78381	-0.44824028	UDP-galactose translocator OS=Homo sapiens OX=9606 GN=SLC35A2 PE=1 SV=1	41.3	2	3	2	5	18.42521667	18.13069153	18.53564072	18.8237648	18.83614922	18.77635574
PLEKHO2	Q8TD55	-0.44748497	Pleckstrin homology domain-containing family O member 2 OS=Homo sapiens OX=9606	53.3	12	37	12	43	20.62756157	20.35380173	20.59149361	20.95304871	20.9671917	20.99507141

			GN=PLEKHO2 PE=1 SV=1												
AKR1A1	P14550	-0.447254181	Aldo-keto reductase family 1 member A1 OS=Homo sapiens OX=9606 GN=AKR1A1 PE=1 SV=3	36.6	18	65	19	60	21.89873695	21.60964012	21.87267494	22.21252441	22.16729546	22.34299469
ALG2	Q9H553	-0.446726481	Alpha-1,3/1,6-mannosyltransferase ALG2 OS=Homo sapiens OX=9606 GN=ALG2 PE=1 SV=1	47.1	7	14	7	17	19.14320374	19.0743084	19.1382122	19.53183746	19.55262375	19.61144257
PRKAA2	P54646	-0.444802284	5'-AMP-activated protein kinase catalytic subunit alpha-2 OS=Homo sapiens OX=9606 GN=PRKAA2 PE=1 SV=2	62.3	2	7	3	6	14.62113571	14.58049393	14.81378078	15.11958981	15.00140858	15.22881889
PALLD	Q8WX93	-0.444272995	Palladin OS=Homo sapiens OX=9606 GN=PALLD PE=1 SV=3	150.5	21	57	22	22	22.0886116	21.72650337	22.07887459	22.44252968	22.3698616	22.41441727
FANCD2	Q9BXW9	-0.441989899	Fanconi anemia group D2 protein OS=Homo sapiens	164	31	61	31	23	21.42726326	21.10789108	21.50203133	21.75531769	21.73892403	21.86891365

			OX=9606 GN=FANCD2 PE=1 SV=2											
PTBP3	O95758	-0.441808065	Polypyrimidine tract-binding protein 3 OS=Homo sapiens OX=9606 GN=PTBP3 PE=1 SV=2	59.7	7	39	10	25	20.21845055	20.17035484	20.36452866	20.63644028	20.66275215	20.77956581
ARL2BP	Q9Y2Y0	-0.441334407	ADP-ribosylation factor-like protein 2-binding protein OS=Homo sapiens OX=9606 GN=ARL2BP PE=1 SV=1	18.8	5	16	5	39	18.55449867	18.39623642	18.73065567	19.01835251	18.97140884	19.01563263
GNB4	Q9HAV0	-0.441134771	Guanine nucleotide-binding protein subunit beta-4 OS=Homo sapiens OX=9606 GN=GNB4 PE=1 SV=3	37.5	4	52	9	31	20.95304871	21.02242279	21.0886116	21.416996	21.40665436	21.56383705
GPX8	Q8TED1	-0.439782461	Probable glutathione peroxidase 8 OS=Homo sapiens OX=9606 GN=GPX8 PE=1 SV=2	23.9	9	24	9	45	21.7875576	21.43237114	21.78356743	22.06907272	22.11743546	22.13633537

PNPT1	Q8TCS8	-0.439476649	Polyribonucleotide nucleotidyltransferase 1, mitochondrial OS=Homo sapiens OX=9606 GN=PNPT1 PE=1 SV=2	85.9	18	40	18	23	20.90976334	20.59149361	20.9671917	21.29333687	21.19460297	21.29893875
DNAJB4	Q9UDY4	-0.438515345	DnaJ homolog subfamily B member 4 OS=Homo sapiens OX=9606 GN=DNAJB4 PE=1 SV=1	37.8	13	55	16	48	21.7634449	21.52611732	21.71816444	22.08537483	22.10469627	22.1332016
CCDC97	Q96F63	-0.438140869	Coiled-coil domain-containing protein 97 OS=Homo sapiens OX=9606 GN=CCDC97 PE=1 SV=1	38.9	5	11	5	24	18.33647156	18.10021019	18.42521667	18.6971035	18.68688393	18.7923336
FAM174A	Q8TBP5	-0.43769296	Membrane protein FAM174A OS=Homo sapiens OX=9606 GN=FAM174A PE=1 SV=1	19.9	2	3	2	14	14.63820934	14.68825054	14.53867435	15.26956463	14.93387508	14.97477341
HIP1R	O75146	-0.437078476	Huntingtin-interacting protein 1-related protein	119.3	18	36	19	19	19.32553291	19.15807724	19.4913044	19.72234154	19.76830101	19.79550743

			OS=Homo sapiens OX=9606 GN=HIP1R PE=1 SV=2											
GCLC	P48506	-0.436051687	Glutamate--cysteine ligase catalytic subunit OS=Homo sapiens OX=9606 GN=GCLC PE=1 SV=2	72.7	13	34	14	24	21.15189934	20.95304871	21.2877121	21.49228287	21.55449867	21.65403366
IMPA1	P29218	-0.435389837	Inositol monophosphatase 1 OS=Homo sapiens OX=9606 GN=IMPA1 PE=1 SV=1	30.2	11	28	11	45	21.0625	20.688591	21.03590584	21.38574409	21.33756065	21.3698616
GLIPR2	Q9H4G4	-0.433678309	Golgi-associated plant pathogenesis-related protein 1 OS=Homo sapiens OX=9606 GN=GLIPR2 PE=1 SV=3	17.2	5	20	5	46	19.26270103	19.05059242	19.39414406	19.63289642	19.61862946	19.75694656
OSTM1	Q86WC4	-0.432118734	Osteopetrosis-associated transmembrane protein 1 OS=Homo sapiens OX=9606	37.2	2	3	2	8	15.91531944	15.91298294	16.00360489	16.35121536	16.36666298	16.41038513

			GN=OSTM1 PE=1 SV=1											
TSR3	Q9UJK0	-0.4318072	18S rRNA aminocarboxypropyl-transferase OS=Homo sapiens OX=9606 GN=TSR3 PE=1 SV=1	33.6	2	4	2	10	15.46403503	15.24004555	15.40872765	15.77828217	15.68002987	15.94991779
GEMIN2	O14893	-0.431404114	Gem-associated protein 2 OS=Homo sapiens OX=9606 GN=GEMIN2 PE=1 SV=1	31.6	3	7	3	17	17.98259163	17.77956581	18.08990479	18.35380173	18.39623642	18.39623642
HEMK2	Q9Y5N5	-0.431298574	Methyltransferase HEMK2 OS=Homo sapiens OX=9606 GN=HEMK2 PE=1 SV=4	22.9	3	5	3	15	17.05324745	17.04260063	17.32333565	17.51267815	17.68002892	17.52037239
MOSPD2	Q8NHP6	-0.431107839	Motile sperm domain-containing protein 2 OS=Homo sapiens OX=9606 GN=MOSPD2 PE=1 SV=1	59.7	14	40	14	29	20.416996	20.15807724	20.51653099	20.77152824	20.7634449	20.84995461
NDOR1	Q9UHB4	-0.430981954	NADPH-dependent diflavin oxidoreductase 1 OS=Homo sapiens	66.7	11	19	11	32	18.90536308	18.82065201	19.0664463	19.28996468	19.35595322	19.43948936

			OX=9606 GN=NDOR1 PE=1 SV=1											
SDAD1	Q9NVU7	-0.430867513	Protein SDA1 homolog OS=Homo sapiens OX=9606 GN=SDAD1 PE=1 SV=3	79.8	12	24	12	18	19.87417603	19.56197357	19.96013832	20.2877121	20.20657539	20.19460297
NDUFA4	O00483	-0.430863063	Cytochrome c oxidase subunit NDUFA4 OS=Homo sapiens OX=9606 GN=NDUFA4 PE=1 SV=1	9.4	5	14	5	56	19.32553291	19.54698563	19.41286659	19.76668358	20.06907272	19.74221802
PTPRE	P23469	-0.430563609	Receptor-type tyrosine-protein phosphatase epsilon OS=Homo sapiens OX=9606 GN=PTPRE PE=1 SV=1	80.6	4	5	4	6	16.8237648	16.53412056	16.7471447	17.10533524	17.14569283	17.14569283
PRKAB1	Q9Y478	-0.42922465	5'-AMP-activated protein kinase subunit beta-1 OS=Homo sapiens OX=9606	30.4	7	17	7	39	20.416996	20.14569283	20.53564072	20.77956581	20.77956581	20.82687187

			GN=PRKAB1 PE=1 SV=4											
GKAP1	Q5VSY0	-0.429153442	G kinase-anchoring protein 1 OS=Homo sapiens OX=9606 GN=GKAP1 PE=1 SV=2	42.1	3	7	3	8	16.46722412	16.27685165	16.54623222	16.919981	16.88464737	16.77313995
TMEM33	P57088	-0.428650538	Transmembrane protein 33 OS=Homo sapiens OX=9606 GN=TMEM33 PE=1 SV=2	28	6	20	6	23	20.50688171	20.63644028	20.45763779	20.98815155	20.9671917	20.93156815
CTU1	Q7Z7A3	-0.428527832	Cytoplasmic tRNA 2-thiolation protein 1 OS=Homo sapiens OX=9606 GN=CTU1 PE=1 SV=1	36.4	2	2	2	11	15.36837006	15.64948082	15.66899586	15.95900536	16.0297184	15.98370647
RTN1	Q16799	-0.427675247	Reticulon-1 OS=Homo sapiens OX=9606 GN=RTN1 PE=1 SV=1	83.6	21	69	22	40	22.60738373	22.31561852	22.63865089	22.96895027	22.93517113	22.94055748
PYGL	P06737	-0.426624298	Glycogen phosphorylase, liver form OS=Homo sapiens OX=9606 GN=PYGL PE=1 SV=4	97.1	36	169	43	54	24.72858238	24.53445244	24.72858238	25.07724571	25.06496811	25.12927628

RAB30	Q15771	-0.426382065	Ras-related protein Rab-30 OS=Homo sapiens OX=9606 GN=RAB30 PE=1 SV=2	23	4	19	5	26	15.64948082	15.69097996	15.85574436	16.01236343	16.11349869	16.34948921
CRELD1	Q96HD1	-0.425731023	Protein disulfide isomerase CRELD1 OS=Homo sapiens OX=9606 GN=CRELD1 PE=1 SV=3	45.4	3	10	3	9	18.47357941	18.22317123	18.50106049	18.78276825	18.79867363	18.89356232
GMFB	P60983	-0.425360998	Glia maturation factor beta OS=Homo sapiens OX=9606 GN=GMFB PE=1 SV=2	16.7	8	28	8	49	21.23608017	21.34299469	21.49716568	21.84995461	21.7634449	21.73892403
EIF2AK2	P19525	-0.423159281	Interferon-induced, double-stranded RNA-activated protein kinase OS=Homo sapiens OX=9606 GN=EIF2AK2 PE=1 SV=2	62.1	16	44	16	35	22.3698616	22.21252441	22.46014023	22.72858238	22.74304008	22.84038162
PEPD	P12955	-0.420321782	Xaa-Pro dipeptidase OS=Homo sapiens OX=9606	54.5	13	35	13	34	20.7471447	20.65403366	20.7634449	21.0886116	21.09506798	21.24190903

			GN=PEPD PE=1 SV=3											
SLC27A2	O14975	-0.420276642	Long-chain fatty acid transport protein 2 OS=Homo sapiens OX=9606 GN=SLC27A2 PE=1 SV=2	70.3	9	19	9	18	19.42726326	19.31229782	19.48345375	19.77152824	19.81284142	19.8994751
KLC2	Q9H0B6	-0.417620977	Kinesin light chain 2 OS=Homo sapiens OX=9606 GN=KLC2 PE=1 SV=1	68.9	20	87	30	54	20.97421265	20.6971035	20.93156815	21.25925636	21.25349617	21.34299469
GLMN	Q92990	-0.417256673	Glomulin OS=Homo sapiens OX=9606 GN=GLMN PE=1 SV=2	68.2	12	23	12	22	19.94592476	19.72733498	20.05589676	20.26499176	20.29893875	20.416996
LARP4	Q71RC2	-0.417178472	La-related protein 4 OS=Homo sapiens OX=9606 GN=LARP4 PE=1 SV=3	80.5	14	29	14	27	19.33429146	19.08990479	19.41905403	19.75205421	19.68346024	19.65927124
CBX6	O95503	-0.417176565	Chromobox protein homolog 6 OS=Homo sapiens OX=9606 GN=CBX6 PE=1 SV=1	43.9	5	8	5	17	18.16054153	17.98259163	18.36238861	18.59877968	18.51653099	18.6417408

UBIAD1	Q9Y5Z9	-0.416984558	UbiA prenyltransferase domain-containing protein 1 OS=Homo sapiens OX=9606 GN=UBIAD1 PE=1 SV=1	36.8	3	4	3	9	16.89652252	16.79867363	17.08472443	17.36666298	17.33210564	17.33210564
RNF166	Q96A37	-0.41655604	E3 ubiquitin-protein ligase RNF166 OS=Homo sapiens OX=9606 GN=RNF166 PE=1 SV=1	26.1	2	6	2	11	15.4893465	15.2474823	15.44151783	15.74976444	15.79360294	15.88464737
CLCC1	Q96S66	-0.415096283	Chloride channel CLIC-like protein 1 OS=Homo sapiens OX=9606 GN=CLCC1 PE=1 SV=1	62	17	41	17	39	21.16422844	20.94592476	21.24190903	21.54037857	21.52133179	21.53564072
ZNHIT6	Q9NWK9	-0.41493988	Box C/D snoRNA protein 1 OS=Homo sapiens OX=9606 GN=ZNHIT6 PE=1 SV=1	53.9	9	15	9	23	18.7634449	18.38784981	18.66622353	19.02106667	19.05589676	18.98537445
ZBTB17	Q13105	-0.414414088	Zinc finger and BTB domain-containing protein 17 OS=Homo sapiens	87.9	1	2	1	2	13.84352875	13.74188805	13.82376575	14.05324745	14.16741848	14.43175888

			OX=9606 GN=ZBTB17 PE=1 SV=3											
CYP2U1	Q7Z449	-0.412371953	Cytochrome P450 2U1 OS=Homo sapiens OX=9606 GN=CYP2U1 PE=1 SV=1	61.9	3	4	3	6	17.29670143	17.08472443	17.25118637	17.66622353	17.55824089	17.64526367
PARP10	Q53GL7	-0.411165873	Protein mono-ADP-ribosyltransferase PARP10 OS=Homo sapiens OX=9606 GN=PARP10 PE=1 SV=2	109.9	11	20	11	15	18.67658997	18.53183746	18.7038765	19.0664463	19.0664463	19.01290894
CHAC2	Q8WUX2	-0.410114606	Glutathione-specific gamma-glutamyl-cyclotransferase 2 OS=Homo sapiens OX=9606 GN=CHAC2 PE=1 SV=1	20.9	1	2	1	9	15.62399578	15.66899586	15.93387508	16.06381607	16.12565613	16.26773834
HTATIP2	Q9BUP3	-0.409985224	Protein HTATIP2 OS=Homo sapiens OX=9606 GN=HTATIP2 PE=1 SV=2	27	8	17	8	34	19.7536869	19.45161438	19.73231316	20.06907272	20.04260063	20.05589676

GPSM1	Q86YR5	- 0.4084008 53	G-protein-signaling modulator 1 OS=Homo sapiens OX=9606 GN=GPSM1 PE=1 SV=2	74.5	14	31	15	28	19.076919 56	18.829971 31	19.053247 45	19.392049 79	19.353801 73	19.439489 36
TMEM192	Q8IY95	- 0.4073168 44	Transmembrane protein 192 OS=Homo sapiens OX=9606 GN=TMEM192 PE=1 SV=1	30.9	3	8	3	14	19.204189 3	18.931568 15	19.145692 83	19.443542 48	19.533741	19.526117 32
HGH1	Q9BTY7	-0.4071757	Protein HGH1 homolog OS=Homo sapiens OX=9606 GN=HGH1 PE=1 SV=1	42.1	7	11	7	26	17.053247 45	17.165456 77	17.194602 97	17.497165 68	17.720672 61	17.416996
PROS1	P07225	- 0.4063618 98	Vitamin K-dependent protein S OS=Homo sapiens OX=9606 GN=PROS1 PE=1 SV=1	75.1	2	2	2	3	16.773139 95	16.462438 58	16.720672 61	17.105335 24	16.954469 68	17.115531 92
AZI2	Q9H6S1	- 0.4037783 94	5-azacytidine-induced protein 2 OS=Homo sapiens OX=9606 GN=AZI2 PE=1 SV=1	44.9	4	8	4	11	15.568668 37	15.368370 06	15.323336 6	15.798674 58	15.707251 55	15.965784 07

RABGAP1	Q9Y3P9	-0.402102788	Rab GTPase-activating protein 1 OS=Homo sapiens OX=9606 GN=RABGAP1 PE=1 SV=3	121.7	32	99	33	34	22.48983574	22.28489113	22.54980659	22.84995461	22.78954887	22.89133835
TPRN	Q4KMQ1	-0.401856422	Taperin OS=Homo sapiens OX=9606 GN=TPRN PE=1 SV=2	75.5	4	6	4	7	15.21371174	15.13570976	15.13570976	15.43175888	15.65508366	15.60385799
TMEM165	Q9HC07	-0.4013532	Putative divalent cation/proton antiporter TMEM165 OS=Homo sapiens OX=9606 GN=TMEM165 PE=1 SV=1	34.9	6	35	6	25	20.04260063	19.7875576	20.1332016	20.39623642	20.40665436	20.36452866
MTHFD1	P11586	-0.401343028	C-1-tetrahydrofolate synthase, cytoplasmic OS=Homo sapiens OX=9606 GN=MTHFD1 PE=1 SV=4	101.5	48	218	49	62	24.93606949	24.65948868	24.94948959	25.24988556	25.22066498	25.27852631
NPHP3	Q7Z494	-0.40080897	Nephrocystin-3 OS=Homo sapiens OX=9606	150.8	4	8	4	4	15.78084755	15.6325407	15.85574436	16.11553192	16.15758324	16.19844437

			GN=NPHP3 PE=1 SV=1											
MTHFD2	P13995	-0.400671005	Bifunctional methylenetetrahydrofolate dehydrogenase/cyclohydrolase, mitochondrial OS=Homo sapiens OX=9606 GN=MTHFD2 PE=1 SV=2	37.9	11	33	11	31	20.90242195	20.688591	20.86514091	21.20060158	21.16422844	21.29333687
TMEM106B	Q9NUM4	-0.399979909	Transmembrane protein 106B OS=Homo sapiens OX=9606 GN=TMEM106B PE=1 SV=2	31.1	3	7	3	12	17.54321289	17.37517548	17.47357941	17.92578697	17.82997131	17.83614922
FERMT2	Q96AC1	-0.398537954	Fermitin family homolog 2 OS=Homo sapiens OX=9606 GN=FERMT2 PE=1 SV=1	77.8	35	132	35	58	23.42981911	23.32388687	23.46762085	23.8190937	23.75938797	23.83845901
FRMD4A	Q9P2Q2	-0.397767385	FERM domain-containing protein 4A OS=Homo sapiens OX=9606	115.4	12	21	12	15	18.70049477	18.69029808	18.72400665	19.08472443	19.10277557	19.12060165

			GN=FRMD4A PE=1 SV=3											
DYNC2I2	Q96EX3	-0.396927516	Cytoplasmic dynein 2 intermediate chain 2 OS=Homo sapiens OX=9606 GN=DYNC2I2 PE=1 SV=2	57.8	9	16	9	23	18.48148346	18.22787857	18.42521667	18.69370461	18.83614922	18.79550743
SLC16A2	P36021	-0.396611532	Monocarboxylate transporter 8 OS=Homo sapiens OX=9606 GN=SLC16A2 PE=1 SV=2	59.5	3	16	3	9	16.0188961	16.1634922	16.28229141	16.6382103	16.41534615	16.60095787
NCS1	P62166	-0.395901362	Neuronal calcium sensor 1 OS=Homo sapiens OX=9606 GN=NCS1 PE=1 SV=2	21.9	7	18	7	45	19.96013832	19.74386215	20.06907272	20.25349617	20.33210564	20.37517548
SACM1L	Q9NTJ5	-0.395420074	Phosphatidylinositol-3-phosphatase SAC1 OS=Homo sapiens OX=9606 GN=SACM1L PE=1 SV=2	66.9	16	34	16	25	21.049263	20.8190937	21.07561493	21.39100075	21.34299469	21.39623642
SKIC8	Q9GZS3	-0.395353317	Superkiller complex protein 8 OS=Homo sapiens OX=9606	33.6	9	22	9	42	20.35380173	20.25349617	20.38574409	20.688591	20.67141724	20.8190937

			GN=SKIC8 PE=1 SV=1											
L2HGDH	Q9H9P8	-0.393247604	L-2-hydroxyglutarate dehydrogenase, mitochondrial OS=Homo sapiens OX=9606 GN=L2HGDH PE=1 SV=3	50.3	8	19	8	19	19.94592476	19.7038765	19.98815155	20.2877121	20.26499176	20.26499176
OGA	O60502	-0.392700195	Protein O-GlcNAcase OS=Homo sapiens OX=9606 GN=OGA PE=1 SV=2	102.8	25	72	25	39	22.05920219	22.01903152	22.1332016	22.49472618	22.47010612	22.4247036
MATN2	O00339	-0.392185211	Matrilin-2 OS=Homo sapiens OX=9606 GN=MATN2 PE=1 SV=4	106.8	3	8	3	4	16.97701073	16.89652252	16.98815155	17.39204979	17.29670143	17.34948921
RBCK1	Q9BYM8	-0.391093572	RanBP-type and C3HC4-type zinc finger-containing protein 1 OS=Homo sapiens OX=9606 GN=RBCK1 PE=1 SV=2	57.5	2	4	2	7	16.7602005	16.5386734	16.60964012	17.08472443	17.04260063	16.95446968

PIKFYVE	Q9Y2I7	-0.390373866	1-phosphatidylinositol 3-phosphate 5-kinase OS=Homo sapiens OX=9606 GN=PIKFYVE PE=1 SV=3	237	11	18	11	6	17.48148346	17.25118637	17.45763779	17.85452843	17.77956581	17.72733498
COA7	Q96BR5	-0.390347163	Cytochrome c oxidase assembly factor 7 OS=Homo sapiens OX=9606 GN=COA7 PE=1 SV=2	25.7	7	15	7	37	20.00195885	19.73562241	19.97421265	20.27639771	20.24190903	20.36452866
STK39	Q9UEW8	-0.390204112	STE20/SPS1-related proline-alanine-rich protein kinase OS=Homo sapiens OX=9606 GN=STK39 PE=1 SV=3	59.4	9	23	10	22	20.24190903	20.09506798	20.36452866	20.60059547	20.59149361	20.68002892
GTPBP2	Q9BX10	-0.388730367	GTP-binding protein 2 OS=Homo sapiens OX=9606 GN=GTPBP2 PE=1 SV=1	65.7	9	20	9	19	18.08472443	17.98815155	18.22787857	18.53183746	18.48148346	18.45362473
FSTL1	Q12841	-0.387565613	Follistatin-related protein 1 OS=Homo sapiens OX=9606	35	15	42	15	53	21.22434998	20.97421265	21.22434998	21.49716568	21.44758415	21.6408596

			GN=FSTL1 PE=1 SV=1											
PLAUR	Q03405	-0.38711675	Urokinase plasminogen activator surface receptor OS=Homo sapiens OX=9606 GN=PLAUR PE=1 SV=1	37	5	11	5	20	17.19460297	17.18495369	17.12565613	17.56569672	17.52037239	17.58049393
HSPE1	P61604	-0.386710485	10 kDa heat shock protein, mitochondrial OS=Homo sapiens OX=9606 GN=HSPE1 PE=1 SV=2	10.9	12	65	12	81	23.9583683	23.71816444	23.97596359	24.36452866	24.19460297	24.25349617
DOP1B	Q9Y3R5	-0.386678696	Protein DOP1B OS=Homo sapiens OX=9606 GN=DOP1B PE=1 SV=5	258.1	35	72	35	19	21.29333687	21.049263	21.31561852	21.63644028	21.54980659	21.6320076
RAL-GAPA1	Q6GYQ0	-0.386392593	Ral GTPase-activating protein subunit alpha-1 OS=Homo sapiens OX=9606 GN=RALGAPA1 PE=1 SV=1	229.7	15	21	15	9	18.20895767	18.16545677	18.33210564	18.66968727	18.55074692	18.64526367

SEC14L1	Q92503	-0.386181196	SEC14-like protein 1 OS=Homo sapiens OX=9606 GN=SEC14L1 PE=1 SV=2	81.2	6	13	6	11	15.8087635	15.95900536	15.97701168	16.3303566	16.22693825	16.34602928
DKK1	O94907	-0.385780334	Dickkopf-related protein 1 OS=Homo sapiens OX=9606 GN=DKK1 PE=1 SV=1	28.7	1	2	1	4	16.18301582	16.10123634	16.39372635	16.62399483	16.5731144	16.6382103
CD2AP	Q9Y5K6	-0.385778427	CD2-associated protein OS=Homo sapiens OX=9606 GN=CD2AP PE=1 SV=1	71.4	32	73	32	59	23.24916267	22.9583683	23.26785278	23.56383705	23.54037857	23.52850342
GNL3L	Q9NVN8	-0.385604858	Guanine nucleotide-binding protein-like 3-like protein OS=Homo sapiens OX=9606 GN=GNL3L PE=1 SV=1	65.5	12	22	13	22	19.38363647	19.15807724	19.48345375	19.73892403	19.67314339	19.76991463
RTCA	O00442	-0.385089874	RNA 3'-terminal phosphate cyclase OS=Homo sapiens OX=9606 GN=RTCA PE=1 SV=1	39.3	13	22	13	47	21.02242279	20.77956581	21.049263	21.34840775	21.29893875	21.35917473

UBFD1	O14562	-0.385016123	Ubiquitin domain-containing protein UBFD1 OS=Homo sapiens OX=9606 GN=UBFD1 PE=1 SV=2	33.4	6	18	6	28	20.7634449	20.56383705	20.83460617	21.10789108	21.08212852	21.12691689
GART	P22102	-0.383388519	Trifunctional purine biosynthetic protein adenosine-3 OS=Homo sapiens OX=9606 GN=GART PE=1 SV=1	107.7	44	167	44	56	24.65403366	24.46762085	24.72858238	24.99334526	24.96278763	25.04426956
LGALS3	P17931	-0.383099874	Galectin-3 OS=Homo sapiens OX=9606 GN=LGALS3 PE=1 SV=5	26.1	9	17	9	37	20.90976334	21.049263	20.98815155	21.38574409	21.49228287	21.21845055
PTPN9	P43378	-0.38250796	Tyrosine-protein phosphatase non-receptor type 9 OS=Homo sapiens OX=9606 GN=PTPN9 PE=1 SV=1	68	8	14	8	14	18.41286659	18.22787857	18.51653099	18.71061897	18.79550743	18.79867363
DOHH	Q9BU89	-0.382469813	Deoxyhypusine hydroxylase OS=Homo sapiens	32.9	4	11	4	28	16.98815155	16.97701073	17.18495369	17.48934555	17.44151688	17.36666298

			OX=9606 GN=DOHH PE=1 SV=1											
RHOBTB3	O94955	-0.382354101	Rho-related BTB domain-containing protein 3 OS=Homo sapiens OX=9606 GN=RHOBTB3 PE=1 SV=2	69.4	3	8	3	5	17.30563354	17.13570976	17.36666298	17.66622353	17.59514046	17.69370461
CTPS1	P17812	-0.379135132	CTP synthase 1 OS=Homo sapiens OX=9606 GN=CTPS1 PE=1 SV=2	66.6	19	69	23	38	22.75327873	22.54274178	22.75938797	23.0375824	23.00024033	23.15499115
GFPT1	Q06210	-0.377892176	Glutamine--fructose-6-phosphate aminotransferase [isomerizing] 1 OS=Homo sapiens OX=9606 GN=GFPT1 PE=1 SV=3	78.8	23	100	28	50	22.62086868	22.47506523	22.68217468	22.99334526	22.94948959	22.96895027
CEP97	Q8IW35	-0.376934052	Centrosomal protein of 97 kDa OS=Homo sapiens OX=9606 GN=CEP97 PE=1 SV=1	96.9	19	61	19	29	20.64526367	20.53564072	20.64526367	20.96013832	20.98119926	21.01563263

SCARB1	Q8WTV0	-0.375790914	Scavenger receptor class B member 1 OS=Homo sapiens OX=9606 GN=SCARB1 PE=1 SV=1	60.8	2	4	2	3	17.37517548	17.08472443	17.30563354	17.65927124	17.60964012	17.62399483
GINS3	Q9BRX5	-0.374401728	DNA replication complex GINS protein PSF3 OS=Homo sapiens OX=9606 GN=GINS3 PE=1 SV=1	24.5	8	14	8	48	19.79867363	19.56383705	19.79867363	20.12060165	20.10789108	20.05589676
OPHN1	O60890	-0.373929977	Oligophrenin-1 OS=Homo sapiens OX=9606 GN=OPHN1 PE=1 SV=1	91.6	8	13	8	10	18.27866745	18.06381607	18.2831974	18.58783531	18.53564072	18.62399483
NDUFA8	P51970	-0.373244603	NADH dehydrogenase [ubiquinone] 1 alpha subcomplex subunit 8 OS=Homo sapiens OX=9606 GN=NDUFA8 PE=1 SV=3	20.1	5	17	5	33	20.20657539	20.00195885	20.18252945	20.50688171	20.4873848	20.51653099
PRKD1	Q15139	-0.372811	Serine/threonine-protein kinase D1	101.6	8	24	12	19	19.43338966	19.25810623	19.47951126	19.77635574	19.72234154	19.79074287

			OS=Homo sapiens OX=9606 GN=PRKD1 PE=1 SV=2											
NR2C2AP	Q86WQ0	-0.372437159	Nuclear receptor 2C2-associated protein OS=Homo sapiens OX=9606 GN=NR2C2AP PE=1 SV=1	15.9	4	12	4	47	18.84536743	18.64877892	18.95730591	19.17035484	19.17523766	19.22317123
ATXN7L3B	Q96GX2	-0.372386297	Ataxin-7-like protein 3B OS=Homo sapiens OX=9606 GN=ATXN7L3B PE=1 SV=2	10.8	2	7	2	31	17.23257065	17.30563354	17.18495369	17.7471447	17.58049393	17.51267815
PASK	Q96RG2	-0.369568507	PAS domain-containing serine/threonine-protein kinase OS=Homo sapiens OX=9606 GN=PASK PE=1 SV=3	142.8	8	16	8	11	17.33210564	17.11553192	17.37517548	17.59514046	17.60240936	17.73396873
FUCA2	Q9BTY2	-0.369178772	Plasma alpha-L-fucosidase OS=Homo sapiens OX=9606 GN=FUCA2 PE=1 SV=2	54	8	14	8	16	19.65927124	19.4636364	19.60059547	19.88315582	19.94592476	20.00195885

WDR62	O43379	-0.368728638	WD repeat-containing protein 62 OS=Homo sapiens OX=9606 GN=WDR62 PE=1 SV=4	165.8	13	35	13	9	19.94592476	19.82531929	19.97421265	20.29893875	20.2877121	20.26499176
RAB27A	P51159	-0.366984049	Ras-related protein Rab-27A OS=Homo sapiens OX=9606 GN=RAB27A PE=1 SV=3	24.9	6	21	8	37	20.00195885	19.82997131	20.02917862	20.27639771	20.32113457	20.36452866
IFI27	P40305	-0.366901716	Interferon alpha-inducible protein 27, mitochondrial OS=Homo sapiens OX=9606 GN=IFI27 PE=1 SV=4	11.5	1	2	1	11	14.19075108	14.2877121	14.21371174	14.6325407	14.67727947	14.48305988
PYROXD2	Q8N2H3	-0.36603864	Pyridine nucleotide-disulfide oxidoreductase domain-containing protein 2 OS=Homo sapiens OX=9606 GN=PYROXD2 PE=1 SV=2	63	2	3	2	4	14.96578407	15.1032877	15.18688774	15.51730061	15.3442955	15.49247932
REXO1	Q8N1G1	-0.365536054	RNA exonuclease 1 homolog OS=Homo sapiens OX=9606	131.4	1	1	1	1	15.6855154	15.63820934	15.73131943	16.1316967	15.98593044	16.03402519

			GN=REXO1 PE=1 SV=3											
CCDC50	Q8IVM0	-0.365291595	Coiled-coil domain-containing protein 50 OS=Homo sapiens OX=9606 GN=CCDC50 PE=1 SV=1	35.8	14	44	14	49	21.0625	20.85756874	21.07561493	21.35380173	21.31561852	21.42213821
DPYSL5	Q9BPU6	-0.364667257	Dihydropyrimidinase-related protein 5 OS=Homo sapiens OX=9606 GN=DPYSL5 PE=1 SV=1	61.4	2	9	2	4	17.17523766	17.02106667	17.18495369	17.45763779	17.50494385	17.51267815
SPNS1	Q9H2V7	-0.363704046	Protein spinster homolog 1 OS=Homo sapiens OX=9606 GN=SPNS1 PE=1 SV=1	56.6	6	13	6	21	19.23022652	19.10021019	19.34516335	19.61503983	19.56383705	19.58783531
GOPC	Q9HD26	-0.363025665	Golgi-associated PDZ and coiled-coil motif-containing protein OS=Homo sapiens OX=9606 GN=GOPC PE=1 SV=1	50.5	20	65	20	56	21.86891365	21.91342163	21.95304871	22.247715	22.24190903	22.33483696

PRMT5	O14744	-0.361652374	Protein arginine N-methyltransferase 5 OS=Homo sapiens OX=9606 GN=PRMT5 PE=1 SV=4	72.6	20	55	20	46	22.57542419	22.42981911	22.62756157	22.88016891	22.90242195	22.93517113
HSPA2	P54652	-0.361312866	Heat shock-related 70 kDa protein 2 OS=Homo sapiens OX=9606 GN=HSPA2 PE=1 SV=1	70	9	182	22	46	20.14569283	19.87117195	20.05589676	20.36452866	20.37517548	20.416996
PCBD2	Q9H0N5	-0.361308098	Pterin-4-alpha-carbinolamine dehydratase 2 OS=Homo sapiens OX=9606 GN=PCBD2 PE=1 SV=4	14.4	1	2	1	12	15.61252308	15.60385799	15.7602005	16.01017761	15.96352863	16.08679962
MAP3K6	O95382	-0.36068217	Mitogen-activated protein kinase kinase kinase 6 OS=Homo sapiens OX=9606 GN=MAP3K6 PE=1 SV=3	142.5	6	9	6	6	17.2877121	17.02106667	17.18495369	17.55824089	17.52037239	17.49716568
MGST2	Q99735	-0.36034584	Microsomal glutathione S-transferase 2 OS=Homo sapiens	16.6	1	3	1	10	16.59950638	16.40540695	16.50649261	16.81127357	16.89652252	16.88464737

			OX=9606 GN=MGST2 PE=1 SV=1											
ATP6V1C1	P21283	-0.360245387	V-type proton ATPase subunit C 1 OS=Homo sapiens OX=9606 GN=ATP6V1C1 PE=1 SV=4	43.9	21	56	21	57	22.28489113	22.12060165	22.39362144	22.61638641	22.56848335	22.69498062
ZMP-STE24	O75844	-0.360127767	CAAX prenyl protease 1 homolog OS=Homo sapiens OX=9606 GN=ZMP-STE24 PE=1 SV=2	54.8	8	24	8	21	21.15807724	20.9671917	21.22434998	21.45763779	21.42726326	21.54510117
SLC5A3	P53794	-0.359380722	Sodium/myo-inositol cotransporter OS=Homo sapiens OX=9606 GN=SLC5A3 PE=1 SV=4	79.6	2	3	2	3	16.09712601	16.05324745	16.05536652	16.41864395	16.3649559	16.50028229
JAGN1	Q8N5M9	-0.356821696	Protein jagunal homolog 1 OS=Homo sapiens OX=9606 GN=JAGN1 PE=1 SV=1	21.1	2	9	2	13	20.35380173	20.35380173	20.52611732	20.73892403	20.83460617	20.73065567

ARL1	P40616	-0.356355031	ADP-ribosylation factor-like protein 1 OS=Homo sapiens OX=9606 GN=ARL1 PE=1 SV=1	20.4	6	20	6	40	21.94234848	21.96013832	22.04260063	22.32388687	22.30452156	22.38574409
OXNAD1	Q96HP4	-0.355584462	Oxidoreductase NAD-binding domain-containing protein 1 OS=Homo sapiens OX=9606 GN=OXNAD1 PE=1 SV=1	34.8	2	2	2	6	17.60240936	17.52037239	17.77313995	17.94306374	17.97701073	18.04260063
FAHD2A	Q96GK7	-0.355442047	Oxaloacetate tautomerase FAHD2A, mitochondrial OS=Homo sapiens OX=9606 GN=FAHD2A PE=1 SV=1	34.6	9	16	9	32	20.35380173	20.24190903	20.47753716	20.67141724	20.688591	20.77956581
TBC1D2	Q9BYX2	-0.354765574	TBC1 domain family member 2A OS=Homo sapiens OX=9606 GN=TBC1D2 PE=1 SV=3	105.3	19	49	19	26	20.73892403	20.51653099	20.7875576	21.00881195	21.04260063	21.05589676
TUFT1	Q9NNX1	-0.35364596	Tuftelin OS=Homo sapiens OX=9606	44.2	4	8	4	13	16.19075012	16.04473495	16.22128487	16.48306084	16.45121193	16.58343506

			GN=TUFT1 PE=1 SV=1												
ATP6V0D1	P61421	-0.350510279	V-type proton ATPase subunit d 1 OS=Homo sapiens OX=9606 GN=ATP6V0D1 PE=1 SV=1	40.3	10	27	10	36	20.60964012	20.60964012	20.66275215	20.98119926	21.04260063	20.90976334
STOM	P27105	-0.347773234	Stomatin OS=Homo sapiens OX=9606 GN=STOM PE=1 SV=3	31.7	9	30	9	46	20.96013832	20.86514091	21.02242279	21.28206635	21.29333687	21.31561852
PFKM	P08237	-0.346437454	ATP-dependent 6-phosphofructokinase, muscle type OS=Homo sapiens OX=9606 GN=PFKM PE=1 SV=2	85.1	16	43	18	28	21.75531769	21.60059547	21.88016891	22.08212852	22.08537483	22.10789108
CCSER2	Q9H7U1	-0.345225652	Serine-rich coiled-coil domain-containing protein 2 OS=Homo sapiens OX=9606 GN=CCSER2 PE=1 SV=2	93.5	3	7	3	5	14.68825054	14.74188805	14.73661327	15.15560913	14.9476366	15.09918308

RAB33B	Q9H082	-0.343824387	Ras-related protein Rab-33B OS=Homo sapiens OX=9606 GN=RAB33B PE=1 SV=1	25.7	2	20	3	13	17.45763779	17.31451225	17.44960022	17.74057198	17.71397781	17.79867363
ALG3	Q92685	-0.339314779	Dol-P-Man:Man(5)GlcNAc(2)-PP-Dol alpha-1,3-mannosyltransferase OS=Homo sapiens OX=9606 GN=ALG3 PE=1 SV=1	50.1	3	5	3	7	16.81127357	16.65228462	16.65228462	16.97701073	17.02106667	17.13570976
DSCC1	Q9BVC3	-0.33751742	Sister chromatid cohesion protein DCC1 OS=Homo sapiens OX=9606 GN=DSCC1 PE=1 SV=2	44.8	6	10	6	16	18.83922768	18.78915215	18.90242195	19.16054153	19.11553192	19.26728058
CD47	Q08722	-0.337490718	Leukocyte surface antigen CD47 OS=Homo sapiens OX=9606 GN=CD47 PE=1 SV=1	35.2	4	12	4	12	19.7055645	19.55824089	19.75694656	20.00195885	20.01563263	20.01563263
SP140L	Q9H930	-0.336846034	Nuclear body protein SP140-like protein OS=Homo	67	6	15	9	15	16.4783268	16.56420898	16.48934555	16.8237648	16.919981	16.79867363

			sapiens OX=9606 GN=SP140L PE=1 SV=3											
PLPP6	Q8IY26	-0.3367939	Polyisoprenoid diphosphate/phosphate phosphohydrolase PLPP6 OS=Homo sapiens OX=9606 GN=PLPP6 PE=1 SV=3	32.2	3	6	3	15	16.35466194	16.38363647	16.51576233	16.68002892	16.81127357	16.77313995
MYD88	Q99836	-0.333960851	Myeloid differentiation primary response protein MyD88 OS=Homo sapiens OX=9606 GN=MYD88 PE=1 SV=1	33.2	7	15	7	34	20.08212852	19.93156815	20.02917862	20.25349617	20.35380173	20.43745995
CALCOCO1	Q9P1Z2	-0.333869934	Calcium-binding and coiled-coil domain-containing protein 1 OS=Homo sapiens OX=9606 GN=CALCOCO1 PE=1 SV=2	77.3	12	16	12	19	18.60240936	18.50106049	18.56197357	18.93156815	18.90242195	18.83306313
TGFB2	P61812	-0.3326238	Transforming growth factor beta-2 proprotein	47.7	3	8	3	8	17.95446968	17.90829849	18.05854225	18.19940376	18.32772827	18.39204979

			OS=Homo sapiens OX=9606 GN=TGFB2 PE=1 SV=1											
RAB32	Q13637	-0.331428528	Ras-related protein Rab-32 OS=Homo sapiens OX=9606 GN=RAB32 PE=1 SV=3	25	9	36	10	48	21.90976334	21.76749229	21.8839016	22.2331562	22.16729546	22.15499115
STAT2	P52630	-0.33063825	Signal transducer and activator of transcription 2 OS=Homo sapiens OX=9606 GN=STAT2 PE=1 SV=1	97.9	13	26	13	20	20.04260063	19.86665154	20.09506798	20.32113457	20.34299469	20.33210564
PM20D2	Q8IYS1	-0.329659144	Xaa-Arg dipeptidase OS=Homo sapiens OX=9606 GN=PM20D2 PE=1 SV=2	47.7	11	40	12	35	21.9916153	21.81518936	22.0122261	22.24481392	22.24481392	22.31838036
DYNLRB1	Q9NP97	-0.329359055	Dynein light chain roadblock-type 1 OS=Homo sapiens OX=9606 GN=DYNLRB1 PE=1 SV=3	10.9	3	13	3	42	22.69922256	22.75531769	22.78156853	23.07071114	23.18101311	22.9724617

ENG	P17813	-0.326569875	Endoglin OS=Homo sapiens OX=9606 GN=ENG PE=1 SV=2	70.5	10	24	10	19	19.87717628	19.78915215	19.96013832	20.19460297	20.15807724	20.25349617
PPP1R37	O75864	-0.326485316	Protein phosphatase 1 regulatory subunit 37 OS=Homo sapiens OX=9606 GN=PPP1R37 PE=1 SV=4	74.7	8	17	8	14	18.43338966	18.27866745	18.31451225	18.59877968	18.67658997	18.73065567
ATP6V1E1	P36543	-0.324494044	V-type proton ATPase subunit E 1 OS=Homo sapiens OX=9606 GN=ATP6V1E1 PE=1 SV=1	26.1	10	37	10	38	21.78356743	21.57773209	21.77956581	22.02917862	22.03590584	22.049263
ARPC1A	Q92747	-0.324268341	Actin-related protein 2/3 complex subunit 1A OS=Homo sapiens OX=9606 GN=ARPC1A PE=1 SV=2	41.5	14	45	15	50	22.27355385	22.12060165	22.33210564	22.57080078	22.51412392	22.61414146
SCARF2	Q96GP6	-0.324244817	Scavenger receptor class F member 2 OS=Homo sapiens OX=9606	92.3	6	8	6	8	17.7072506	17.65228462	17.81127357	18.02647972	18.05324745	18.06381607

			GN=SCARF2 PE=1 SV=5											
PSRC1	Q6PGN9	-0.323160807	Proline/serine-rich coiled-coil protein 1 OS=Homo sapiens OX=9606 GN=PSRC1 PE=1 SV=1	38.8	5	12	5	16	17.05324745	17.01017761	17.10533524	17.46562958	17.31451225	17.35810089
RRS1	Q15050	-0.322447459	Ribosome biogenesis regulatory protein homolog OS=Homo sapiens OX=9606 GN=RRS1 PE=1 SV=2	41.2	17	40	17	41	21.81518936	21.75938797	21.85376549	22.13946152	22.09506798	22.1611557
NOCT	Q9UK39	-0.322187742	Nocturnin OS=Homo sapiens OX=9606 GN=NOCT PE=1 SV=2	48.2	1	3	1	3	14.97028542	15.04899788	14.9970026	15.36495495	15.38532352	15.23257065
CGAS	Q8N884	-0.321791331	Cyclic GMP-AMP synthase OS=Homo sapiens OX=9606 GN=CGAS PE=1 SV=2	58.8	13	33	13	32	19.9185257	19.93156815	20.00195885	20.26499176	20.25349617	20.29893875
IBTK	Q9P2D0	-0.32167689	Inhibitor of Bruton tyrosine kinase OS=Homo sapiens	150.4	11	19	11	11	17.85452843	17.88464737	17.91415215	18.14569283	18.2877121	18.18495369

			OX=9606 GN=IBTK PE=1 SV=3											
CLCN7	P51798	-0.318943024	H(+)/Cl(-) exchange transporter 7 OS=Homo sapiens OX=9606 GN=CLCN7 PE=1 SV=2	88.6	5	11	5	7	18.33210564	18.33647156	18.39204979	18.68688393	18.71732903	18.6132431
KIF7	Q2M1P5	-0.317581177	Kinesin-like protein KIF7 OS=Homo sapiens OX=9606 GN=KIF7 PE=1 SV=2	150.5	18	40	19	18	19.65752792	19.59877968	19.7536869	19.97421265	19.94592476	20.04260063
MAP1B	P46821	-0.317134221	Microtubule-associated protein 1B OS=Homo sapiens OX=9606 GN=MAP1B PE=1 SV=2	270.5	96	419	96	52	25.02326775	24.89966011	25.07316399	25.32388687	25.3066082	25.31699944
TBC1D24	Q9ULP9	-0.316740672	TBC1 domain family member 24 OS=Homo sapiens OX=9606 GN=TBC1D24 PE=1 SV=2	62.9	5	9	5	9	18.29670143	18.15560913	18.24190903	18.53183746	18.48148346	18.63112068
RAB43	Q86YS6	-0.315021515	Ras-related protein Rab-43 OS=Homo sapiens OX=9606	23.3	4	24	6	26	17.05324745	17.04260063	17.17523766	17.45763779	17.35810089	17.40041161

			GN=RAB43 PE=1 SV=1											
TGS1	Q96RS0	-0.311003367	Trimethylguanosine synthase OS=Homo sapiens OX=9606 GN=TGS1 PE=1 SV=3	96.6	4	8	4	7	16.17718506	16.07221603	16.04473495	16.35466194	16.40207863	16.47040558
C8orf82	Q6P1X6	-0.310002645	UPF0598 protein C8orf82 OS=Homo sapiens OX=9606 GN=C8orf82 PE=1 SV=2	23.9	4	6	4	24	17.90829849	17.85452843	17.93732834	18.17035484	18.19940376	18.26040459
MCL1	Q07820	-0.308101018	Induced myeloid leukemia cell differentiation protein Mcl-1 OS=Homo sapiens OX=9606 GN=MCL1 PE=1 SV=3	37.3	3	6	3	20	17.30563354	17.24190903	17.19460297	17.52037239	17.58783531	17.55824089
PRKAG1	P54619	-0.299621582	5'-AMP-activated protein kinase subunit gamma-1 OS=Homo sapiens OX=9606 GN=PRKAG1 PE=1 SV=1	37.6	8	24	9	32	21.32663155	21.23608017	21.31561852	21.54980659	21.57773209	21.6496563

ARF4	P18085	-0.297040304	ADP-ribosylation factor 4 OS=Homo sapiens OX=9606 GN=ARF4 PE=1 SV=3	20.5	5	103	9	73	20.37517548	20.34299469	20.38574409	20.60964012	20.67141724	20.71397781
FOXRED2	Q8IWF2	-0.292235057	FAD-dependent oxidoreductase domain-containing protein 2 OS=Homo sapiens OX=9606 GN=FOXRED2 PE=1 SV=1	77.7	3	4	3	5	15.45763779	15.54773808	15.52955246	15.74188805	15.84597969	15.82376575
LARP1B	Q659C4	-0.287970861	La-related protein 1B OS=Homo sapiens OX=9606 GN=LARP1B PE=1 SV=2	105.3	2	10	4	4	16.81127357	16.68002892	16.7072506	16.98815155	17.02106667	17.05324745
MAP2K7	O14733	-0.28705279	Dual specificity mitogen-activated protein kinase kinase 7 OS=Homo sapiens OX=9606 GN=MAP2K7 PE=1 SV=2	47.5	7	11	7	18	19.69879913	19.56383705	19.59514046	19.87117195	19.88762474	19.96013832
MCCC1	Q96RQ3	-0.285862605	Methylcrotonoyl-CoA carboxylase subunit alpha, mitochondrial	80.4	7	17	7	13	18.31451225	18.31007957	18.33210564	18.60602951	18.62042046	18.58783531

			OS=Homo sapiens OX=9606 GN=MCCC1 PE=1 SV=3											
CAD	P27708	-0.2852904	Multifunctional protein CAD OS=Homo sapiens OX=9606 GN=CAD PE=1 SV=3	242.8	55	168	59	37	23.85756874	23.74919128	23.85756874	24.08537483	24.10948563	24.12533951
CEP85	Q6P2H3	-0.275129954	Centrosomal protein of 85 kDa OS=Homo sapiens OX=9606 GN=CEP85 PE=1 SV=1	85.6	12	26	12	20	19.1801033	19.21608353	19.20895767	19.45161438	19.43948936	19.53943062
GYG1	P46976	-0.259875615	Glycogenin-1 OS=Homo sapiens OX=9606 GN=GYG1 PE=1 SV=4	39.4	7	23	7	22	21.90609741	21.88762474	21.8839016	22.18555832	22.09828377	22.17340851
ACAD11	Q709F0	-0.257143021	Acyl-CoA dehydrogenase family member 11 OS=Homo sapiens OX=9606 GN=ACAD11 PE=1 SV=3	87.2	3	7	3	5	17.49716568	17.46562958	17.46562958	17.77956581	17.7471447	17.67314339

PRKACB	P22694	-0.256607056	cAMP-dependent protein kinase catalytic subunit beta OS=Homo sapiens OX=9606 GN=PRKACB PE=1 SV=2	40.6	8	49	16	54	20.32113457	20.27639771	20.33210564	20.60059547	20.58233261	20.51653099
IRS2	Q9Y4H2	-0.255858103	Insulin receptor substrate 2 OS=Homo sapiens OX=9606 GN=IRS2 PE=1 SV=2	137.2	6	8	6	5	15.9246273	15.93617821	15.95900536	16.17328644	16.2041893	16.20990944
ITM2B	Q9Y287	-0.249528885	Integral membrane protein 2B OS=Homo sapiens OX=9606 GN=ITM2B PE=1 SV=1	30.3	6	10	6	29	17.44151688	17.44151688	17.48934555	17.67314339	17.74057198	17.7072506

Table S6. PANTHER signaling pathway enrichment analysis using overexpressed proteins in INT-SFT-H cells from mass spectrometry. Significantly enriched pathways ($p < 0.05$) derived from candidate overexpressed proteins in INT-SFT cells

PANTHER Pathways	Related Proteins
5HT1 type receptor mediated signaling pathway (P04373)	PRKAR2B
Alzheimer disease-amyloid secretase pathway (P00003)	KLC4
Angiogenesis (P00005)	SPHK1,PDG-FRB,PIK3C2A
Apoptosis signaling pathway (P00006)	MADD
Axon guidance mediated by netrin (P00009)	PIK3C2A
Beta1 adrenergic receptor signaling pathway (P04377)	PRKAR2B
Beta2 adrenergic receptor signaling pathway (P04378)	PRKAR2B
CCKR signaling map (P06959)	RPS6KB1, DNM1, TRAF6, ACAT1
Cobalamin biosynthesis (P02735)	TIGAR
Dopamine receptor mediated signaling pathway (P05912)	PRKAR2B, EPB41
EGF receptor signaling pathway (P00018)	PIK3C2A
Endothelin signaling pathway (P00019)	PRKAR2B, PIK3C2A
Enkephalin release (P05913)	PRKAR2B
FGF signaling pathway (P00021)	PIK3C2A
GABA-B receptor II signaling (P05731)	PRKAR2B
Gonadotropin-releasing hormone receptor pathway (P06664)	DNM1,ITGA1,SDF4
Hedgehog signaling pathway (P00025)	PRKAR2B
Heterotrimeric G-protein signaling pathway-Gi alpha and Gs alpha mediated pathway (P00026)	PRKAR2B,RGS19,GYS1
Heterotrimeric G-protein signaling pathway-Gq alpha and Go alpha mediated pathway (P00027)	RGS19
Histamine H2 receptor mediated signaling pathway (P04386)	PRKAR2B
Huntington disease (P00029)	ARFIP2
Hypoxia response via HIF activation (P00030)	PIK3C2A
Insulin/IGF pathway-mitogen activated protein kinase kinase/MAP kinase cascade (P00032)	RPS6KB1
Insulin/IGF pathway-protein kinase B signaling cascade (P00033)	PIK3C2A
Integrin signalling pathway (P00034)	ITGA1,LAMC1,PIK3C2A,COL18A1,ITGA3
Interferon-gamma signaling pathway (P00035)	PIAS3
Interleukin signaling pathway (P00036)	CDKN1A
JAK/STAT signaling pathway (P00038)	PIAS3
Metabotropic glutamate receptor group II pathway (P00040)	PRKAR2B

Metabotropic glutamate receptor group III pathway (P00039)	PRKAR2B
Muscarinic acetylcholine receptor 2 and 4 signaling pathway (P00043)	PRKAR2B
Nicotine pharmacodynamics pathway (P06587)	EPB41
Opioid prodynorphin pathway (P05916)	NEU1
Oxidative stress response (P00046)	DUS2
PDGF signaling pathway (P00047)	RPS6KB1, PDGFRB
PI3 kinase pathway (P00048)	RPS6KB1
Parkinson disease (P00049)	CUL2
Salvage pyrimidine ribonucleotides (P02775)	UPP1
TGF-beta signaling pathway (P00052)	TLL1,CERS1
Toll receptor signaling pathway (P00054)	TRAF6
Transcription regulation by bZIP transcription factor (P00055)	PRKAR2B
VEGF signaling pathway (P00056)	SPHK1,PIK3C2A
Vasopressin synthesis (P04395)	NEU1
p38 MAPK pathway (P05918)	TRAF6
p53 pathway by glucose deprivation (P04397)	RPS6KB1
p53 pathway feedback loops 2 (P04398)	CDKN1A,PIK3C2A
p53 pathway (P00059)	CDKN1A,DDB2,PIK3C2A,CDKN2A

Table S7. Common PANTHER signaling pathways identified from both RNA-seq and mass spectrometry assays. Column description: Common PANTHER Pathways: names and PANTHER accession numbers for signaling pathways identified in both RNA-seq and mass spectrometry assays; **Related Genes:** the specific differentially expressed genes for the candidate signaling pathway; **Related Proteins:** the specific differentially expressed proteins for the candidate signaling pathway; **Common Genes/Proteins:** the common genes and proteins from both RNA-seq and mass spectrometry assays.

Common PANTHER Pathways	Related Genes	Related Proteins	Common Genes/Proteins
Angiogenesis (P00005)	EPHA3, WNT2, FZD8, SFRP1, EPHB3, LPXN, PDGFA	SPHK1, PDGFRB, PIK3C2A	--
Alzheimer disease-amyloid secretase pathway (P00003)	BACE2, APBA2	KLC4	--
Gonadotropin-releasing hormone receptor pathway (P06664)	PBX1, NFATC1	DNM1, ITGA1, SDF4	--
Heterotrimeric G-protein signaling pathway-Gq alpha and Go alpha mediated pathway (P00027)	AC004754, RGS19	RGS19	RGS19
Heterotrimeric G-protein signaling pathway-Gi alpha and Gs alpha mediated pathway (P00026)	AC004754, RGS19	PRKAR2B, RGS19, GYS1	RGS19
Integrin signalling pathway (P00034)	LAMC1, COL11A1	ITGA1, LAMC1, PIK3C2A, COL18A1, ITGA3	LAMC1

Interleukin signaling pathway (P00036)	CDKN1A, RPS6KA2	CDKN1A	CDKN1A
Muscarinic acetylcholine receptor 2 and 4 signaling pathway (P00043)	ACHE, BCHE	PRKAR2B	--
PDGF signaling pathway (P00047)	RPS6KA2, STARD8, PDGFA	RPS6KB1, PDGFRB	--
TGF-beta signaling pathway (P00052)	GDF15, GDF5	TLL1, CERS1	--
p53 pathway (P00059)	CDKN1A, DDB2, IGFBP3, TNFRSF10D	CDKN1A, DDB2, PIK3C2A, CDKN2A	CDKN1A,DDB2
Metabotropic glutamate receptor group III pathway (P00039)	GRIA1	PRKAR2B	--
PI3 kinase pathway (P00048)	CCND2	RPS6KB1	--
Parkinson disease (P00049)	SNCAIP	CUL2	--
p53 pathway feedback loops 2 (P04398)	CDKN1A	CDKN1A,PIK3C2A	CDKN1A

Table S8. Upregulated genes in both INT-SFT-H vs. INT-SFT-L cells and Lf-4-2 vs. Lf-C2 from RNA-seq. Column description: gene_id: Ensembl gene ID number; **baseMean_INT-SFT:** The average of the normalized count values for the gene across all samples in INT-SFT-H and INT-SFT-L cells; **log2FoldChange_INT-SFT:** The estimated effect size between the two groups being compared in INT-SFT-H and INT-SFT-L cells; **pvalue_INT-SFT:** The unadjusted p-value associated with the Wald statistic in INT-SFT-H and INT-SFT-L cells; **padj_INT-SFT:** The p-value adjusted for multiple testing using the Benjamini-Hochberg (BH) procedure in INT-SFT-H and INT-SFT-L cells; **baseMean_Lf:** The average of the normalized count values for the gene across all samples in Lf-4-2 and Lf-C2 cells; **log2FoldChange_Lf:** The estimated effect size between the two groups being compared in Lf-4-2 and Lf-C2 cells; **pvalue_Lf:** The unadjusted p-value associated with the Wald statistic in Lf-4-2 and Lf-C2 cells; **padj_Lf:** The p-value adjusted for multiple testing using the Benjamini-Hochberg (BH) procedure in Lf-4-2 and Lf-C2 cells.

gene_id	base-Mean_INT-SFT	log2Fold-Change_INT-SFT	pvalue_INT-SFT	padj_INT-SFT	base-Mean_Lf	log2Fold-Change_Lf	pvalue_Lf	padj_Lf
ENSG00000129910|CDH15	13.25626136	7.242357444	0.000911933	0.011415489	12.05345714	3.707053604	0.002148378	0.006978265
ENSG00000125965|GDF5	57.81509608	4.306827237	1.08E-07	5.22E-06	173.9381088	3.053166284	9.91E-17	1.51E-15
ENSG00000018625|ATP1A2	254.8087497	3.894230253	0.000349892	0.005283497	21.57904241	2.886366384	0.00106425	0.003698688
ENSG00000187800|PEAR1	78.70647816	2.987307096	3.12E-11	3.08E-09	1586.533167	2.357641673	4.98E-92	1.26E-89
ENSG00000006042|TMEM98	32.06950486	1.921703741	0.006221176	0.049414889	110.5066855	1.242124525	0.000444367	0.001669752
ENSG00000105650|PDE4C	32.30799693	1.823702787	0.003816135	0.034145907	14.48853861	7.557684182	0.000229739	0.000917161
ENSG00000064692|SNCAIP	210.9559957	1.690796646	8.08E-12	9.05E-10	16.0493266	3.525155575	0.000298869	0.001166087
ENSG00000141934|PLPP2	100.8289738	1.526274007	5.59E-05	0.001182253	82.30068475	3.290882887	9.62E-14	1.15E-12
ENSG00000165821|SALL2	116.5508179	1.469539696	8.06E-06	0.00023281	333.5963878	2.49309668	1.56E-34	6.32E-33
ENSG00000196611|MMP1	170.9932671	1.453290433	1.40E-07	6.61E-06	24085.85136	2.310859718	0	0

ENSG00000100100|PIK3IP1	215.7112089	1.405079431	3.01E-08	1.68E-06	585.7328506	1.983251287	7.83E-37	3.54E-35
ENSG00000159761|C16orf86	75.81536045	1.328998796	0.004134298	0.036195823	84.47711738	1.796336305	4.22E-06	2.22E-05
ENSG00000167779|IGFBP6	1434.968464	1.245406605	2.58E-19	6.45E-17	13263.26567	1.704459817	2.61E-115	1.26E-112
ENSG00000139200|PIANP	728.6482625	1.212824696	1.13E-10	1.00E-08	413.8998863	1.440984079	1.37E-15	1.92E-14
ENSG00000129757|CDKN1C	130.9454216	1.205011069	0.000766802	0.009935188	72.96255838	1.934265757	1.78E-05	8.55E-05
ENSG00000164849|GPR146	137.2513454	1.184495356	0.00055586	0.007752313	17.7108419	2.922077451	0.00140334	0.00474558
ENSG00000159388|BTG2	249.9700272	1.156238143	8.61E-07	3.34E-05	200.1011526	1.212274891	8.36E-07	4.89E-06
ENSG00000186918|ZNF395	4325.680534	1.150682456	6.21E-32	3.87E-29	2136.587751	1.264303771	0.000936078	0.003285212
ENSG00000130513|GDF15	640.3689643	1.12338193	1.73E-09	1.21E-07	280.1952681	1.70945717	7.02E-12	7.08E-11
ENSG00000185522|LMNTD2	216.6526399	1.070431903	0.000584756	0.008046149	24.30604819	3.317582623	0.000629109	0.002288957
ENSG00000127561|SYNGR3	326.4666895	1.065235938	1.45E-07	6.87E-06	228.2597091	1.362414441	5.56E-09	4.24E-08
ENSG00000154310|TNIK	679.6618769	1.038504175	1.05E-10	9.40E-09	69.09653335	3.568783676	1.54E-14	1.97E-13

Table S9. Downregulated genes in both INT-SFT-H vs. INT-SFT-L cells and Lf-4-2 vs. Lf-C2 from RNA-seq. Column description: gene_id: Ensembl gene ID number; **baseMean_INT-SFT:** The average of the normalized count values for the gene across all samples in INT-SFT-H and INT-SFT-L cells; **log2FoldChange_INT-SFT:** The estimated effect size between the two groups being compared in INT-SFT-H and INT-SFT-L cells; **pvalue_INT-SFT:** The unadjusted p-value associated with the Wald statistic in INT-SFT-H and INT-SFT-L cells; **padj_INT-SFT:** The p-value adjusted for multiple testing using the Benjamini-Hochberg (BH) procedure in INT-SFT-H and INT-SFT-L cells; **baseMean_Lf:** The average of the normalized count values for the gene across all samples in Lf-4-2 and Lf-C2 cells; **log2FoldChange_Lf:** The estimated effect size between the two groups being compared in Lf-4-2 and Lf-C2 cells; **pvalue_Lf:** The unadjusted p-value associated with the Wald statistic in Lf-4-2 and Lf-C2 cells; **padj_Lf:** The p-value adjusted for multiple testing using the Benjamini-Hochberg (BH) procedure in Lf-4-2 and Lf-C2 cells.

gene_id	base-Mean_INT-SFT	log2Fold-Change_INT-SFT	pvalue_INT-SFT	padj_INT-SFT	base-Mean_Lf	log2Fold-Change_Lf	pvalue_Lf	padj_Lf
ENSG00000138944|KIAA1644	233.9163885	-1.768138792	4.62E-14	6.79E-12	203.3548228	-2.09060517	7.14E-16	1.02E-14
ENSG00000172159|FRMD3	90.80755009	-1.776954438	1.56E-05	0.000399612	5167.062822	-1.00596825	4.28E-38	2.04E-36
ENSG00000026559|KCNG1	42.3902757	-1.998547933	0.000159884	0.00277366	230.7421612	-1.60750565	1.21E-08	8.84E-08
ENSG00000163017|ACTG2	2278.625528	-2.323317462	3.01E-80	2.63E-76	1239.170284	-3.698868388	1.94E-167	2.30E-164

ENSG00000267886|CTD-2291D10.4	28.32336984	-3.120321122	0.000395472	0.005876597	17.33914695	-3.531945267	0.000948808	0.003326803

Table S10. List of primer sequences.

Sequence (5′ → 3′)
CAGTACGAATTCCTCGAGACTAGTGCCACCATGGATTACAAAG
CAGTACACGCGTTTAACCTCCTCCACCTCTGTGCCCCAGTTTGCTAGG
CAGTACGCATGCGAGGGCCTATTTCCCATGATTCCTTCATATT
GTACGCATGCCAACTTTTCTATACAAAGTTGTCTAGAAAAAAC-CTAAGGTTAAGTCGCCCTCGCTCGAGCGAGGGCGACTTAACCTTAGGCCGGTGTTTCGTCCTTTCCACAAGATATA
CAGTACATCGATGAGTAATTCATACAAAAGGACTCGC
CAGTACTCTAGAAAAAAGCTCCTGACAGAACCAGAAATCTCGAGAT-TTCTGGTTCTGTCAGGAGCCCGGTGTTTCGTCCTTTCCACAAGATATATA
AATCCCATCACCATCTTCCA
TGGACTCCACGACGTACTCA
AACCTTTCTCCTCCGCTTCA
TGGCTGGATGTTCTCTATCTGT
CGAAGCCACCTCTCGCAG
CTTGTAGTGGCTCCGGAAAG
CTGTGAGGTCAACCACTTTGGG
AGCCTTCTCTGCATTCACAGCG
